# Redox-neutral manganese-catalyzed synthesis of 1-pyrrolines†

**DOI:** 10.1039/d2sc00015f

**Published:** 2022-02-09

**Authors:** Tingting Feng, Canxiang Liu, Zhen Wu, Xinxin Wu, Chen Zhu

**Affiliations:** Key Laboratory of Organic Synthesis of Jiangsu Province, College of Chemistry, Chemical Engineering and Materials Science, Soochow University 199 Ren-Ai Road Suzhou Jiangsu 215123 People's Republic of China chzhu@suda.edu.cn xxwu99@suda.edu.cn; Frontiers Science Center for Transformative Molecules, Shanghai Jiao Tong University 800 Dongchuan Road Shanghai 200240 People's Republic of China

## Abstract

This report describes a manganese-catalyzed radical [3 + 2] cyclization of cyclopropanols and oxime ethers, leading to valuable multi-functional 1-pyrrolines. In this redox-neutral process, the oxime ethers function as internal oxidants and H-donors. The reaction involves sequential rupture of C–C, C–H and N–O bonds and proceeds under mild conditions. This intermolecular protocol provides an efficient approach for the synthesis of structurally diverse 1-pyrrolines.

## Abstract 2

Described herein is a novel manganese-catalyzed radical [3 + 2] cyclization of cyclopropanols and oxime ethers, leading to valuable multi-functionalized 1-pyrrolines.

## Graphical abstract

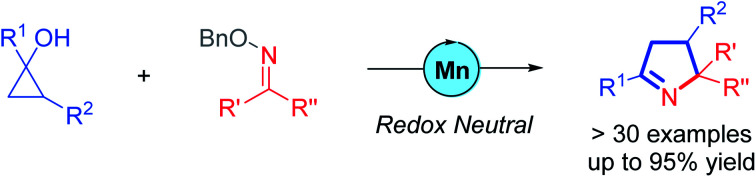

Pyrroline and its derivatives appear frequently as the core of the structure of natural products and biologically active molecules (Fig. 1A).^1^ Such compounds also serve as versatile feedstocks in various transformations, such as 1,3-dipolar cyclization, ring opening, reduction and oxidation, leading to diverse and valuable compounds.^2–4^ Over the past few decades, great effort has been devoted to the preparation of pyrrolines. This has resulted in several elegant approaches that rely on photoredox catalysis (Fig. 1B).^5^ The groups of Studer,^5*a*,*b*^ Leonori,^5*c*^ and Loh^5*d*–*f*^ disclosed intramolecular addition of the intermediate iminyl radical to alkenes to construct pyrrolines. Generally, the synthetic value of a method can be further improved by using an intermolecular reaction pattern. For example, Alemán *et al.* recently reported a radical-polar cascade reaction involving the addition to ketimines of alkyl radicals formed in hydrogen atom transfer (HAT) reactions.^5*g*^ That the existence of benzylic C–H bonds in the substrates is requisite for the HAT, compromises the substrate scope. Despite the appealing photochemical processes, development of new redox approaches to enrich the product diversity of pyrrolines, especially with inexpensive transition-metal catalysts, is still in demand.

**Fig. 1:**
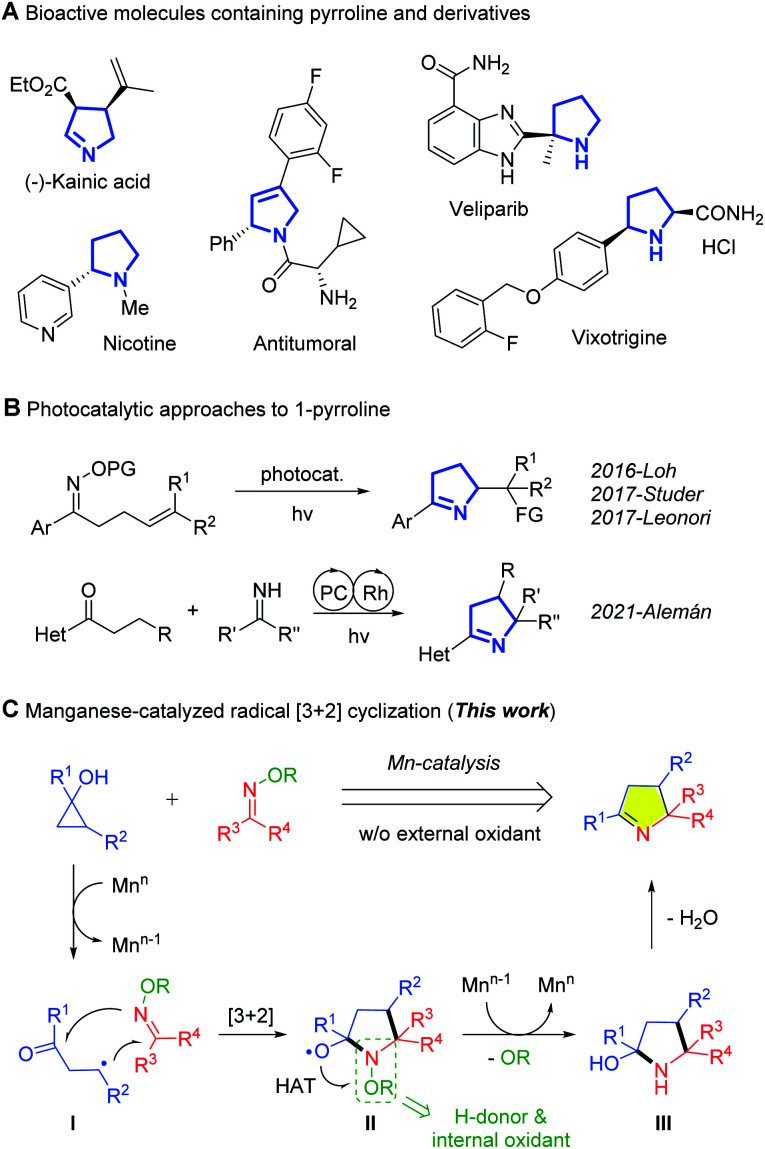
(A) Importance of pyrrolines, and (B and C) synthetic approaches to pyrrolines.

Prompted by extensive applications of cyclopropanols in synthesis^6^ and our achievements in manganese-catalyzed ring-opening reactions,^7^ we conceived a radical [3 + 2] cyclization using cyclopropanol as a C3 synthon and oxime ethers as a nitrogen source (Fig. 1C). Hypothetically, single-electron oxidation of cyclopropanol by Mn^*n*^ generates the β-keto radical (I), which undergoes a radical [3 + 2] cascade reaction with an oxime ether to give the alkoxy radical species (II). Conversion of II to the intermediate (III), the pyrroline precursor, requires an extra H-donor to support a HAT process and an oxidant for recovery of Mn^*n*^ to perpetuate the catalytic cycle. In this scenario, the strategic inclusion of oxime ether is crucial to the overall transformation. The oxime ether is not only an internal oxidant and H-donor, but should also be subject to *in situ* deprotection by cleaving the N–O bond during the reaction. The choice of a proper Mn^*n*^/Mn^*n*−1^ pair with suitable redox potentials is also vital to the catalytic cycle.

Herein, we provide proof-of-principle studies for this hypothesis. The desired radical [3 + 2] cyclization of cyclopropanols and *O*-benzyl oxime ethers is accomplished with manganese catalysis. This redox-neutral process involves sequential rupture of C–C, C–H, and N–O bonds under mild conditions. The intermolecular protocol provides an ingenious approach to the synthesis of multi-functionalized 1-pyrrolines.

With these considerations in mind, phenylcyclopropanol (1a) and oxime ether (2a) were initially chosen as model substrates to evaluate reaction parameters in the presence of manganese salt (Table 1). With the use of 1.7 equiv. of manganic acetylacetonate (Mn(acac)_3_) and acetic acid, the pyrroline product (3a) was readily obtained at room temperature, albeit in low yield (entry 1). Acetic acid is crucial to the transformation in this case, presumably serves to activate the C=N bond of 2a (entry 2). The optimization of organic solvents was then conducted (entries 3–8), and it was found that the use of fluorinated alcohols, such as trifluoroethanol (TFE) and hexafluoroisopropanol (HFIP) as solvents provided excellent yields (entries 7 and 8). Decreasing the amount of Mn(acac)_3_ to 1.2 equiv. gave a comparable yield (entry 9), but further reducing the amount compromised the yield (entry 10). Replacing Mn(acac)_3_ with Mn(OAc)_3_ or MnCl_2_ significantly decreased the reaction yield (entries 11 and 12). However, the use of Mn(acac)_2_ gave a similar yield to Mn(acac)_3_ (entries 13 *vs.* 9). The above results prompted us to think over the counteranion effect that the acetylacetone (acac) anion may be requisite to the reaction. Indeed, the synergistic use of stoichiometric MnCl_2_ and acetylacetone led to a good yield of the desired product (entry 14). More importantly, a comparable yield was obtained with only 0.2 equiv. of MnCl_2_ and added acetylacetone, realizing this reaction under a catalytic amount of Mn salts (entry 15). Given that the low solubility of the Mn salt may lead to poor efficiency, a reaction with 0.067 M concentration was carried out and gave a 89% yield (entry 16). Further reducing the amount of acetylacetone to 1.0 equiv. had no influence on the outcome of the reaction (entry 17), but the reaction efficiency slightly decreased when 0.6 equiv. of acetylacetone was used as the additive (entry 18). Use of a decreased amount (1.0 equiv.) of acetic acid led to the best yield (91%, entry 19), whereas the reaction in the presence of 0.5 equiv. acetic acid (entry 20) or without acetic acid (entry 21) also gave high yields. It is noted that acetic acid is not crucial to the reaction using MnCl_2_ as catalyst, as the reaction could generate cat. HCl *in situ*. The reaction with substoichiometric amount (0.6 equiv.) of acac gave a decreased but also good yield (entry 22). Reducing the catalytic loading of MnCl_2_ to 10 mol% slightly compromised the yield (entry 23).

**Table 1:** Optimization of the synthesis of 1-pyrrolines

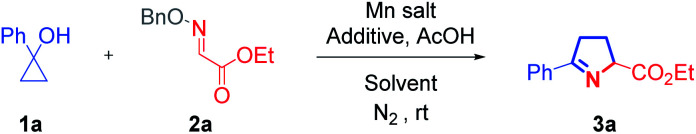				
Entry^a^	Mn salt (equiv.)	Additive (equiv.)	Solvent	Yield (%)
1	Mn(acac)_3_ (1.7)	None	CH_3_CN	33
2^b^	Mn(acac)_3_ (1.7)	None	CH_3_CN	Trace
3	Mn(acac)_3_ (1.7)	None	DCM	31
4	Mn(acac)_3_ (1.7)	None	Acetone	25
5	Mn(acac)_3_ (1.7)	None	DMSO	Trace
6	Mn(acac)_3_ (1.7)	None	DMF	Trace
7	Mn(acac)_3_ (1.7)	None	TFE	80
8	Mn(acac)_3_ (1.7)	None	HFIP	82
9	Mn(acac)_3_ (1.2)	None	HFIP	83
10	Mn(acac)_3_ (0.9)	None	HFIP	55
11	Mn(OAc)_3_·2H_2_O (1.2)	None	HFIP	36
12	MnCl_2_ (1.2)	None	HFIP	Trace
13	Mn(acac)_2_ (1.2)	None	HFIP	88
14	MnCl_2_ (1.2)	acac (3.6)	HFIP	80
15	MnCl_2_ (0.2)	acac (3.6)	HFIP	81
16^c^	MnCl_2_ (0.2)	acac (3.6)	HFIP	89
17^c^	MnCl_2_ (0.2)	acac (1.0)	HFIP	89
18^c^	MnCl_2_ (0.2)	acac (0.6)	HFIP	83
19^c^^,^^d^	MnCl_2_ (0.2)	acac (1.0)	HFIP	91
20^c^^,^^e^	MnCl_2_ (0.2)	acac (1.0)	HFIP	83
21^c^^,^^b^	MnCl_2_ (0.2)	acac (1.0)	HFIP	80
22^c^^,^^d^	MnCl_2_ (0.2)	acac (0.6)	HFIP	82
23^c^^,^^d^	MnCl_2_ (0.1)	acac (1.0)	HFIP	81

a Reaction conditions: 1a (0.45 mmol), 2a (0.3 mmol), AcOH (2.0 equiv.), and Mn salt (as shown) in solvent (2.0 mL), at room temperature (rt) under N_2_, for 16 h.

b Without AcOH.

c 0.067 M reaction.

d 1.0 equiv. AcOH.

e 0.5 equiv. AcOH. acac = acetylacetone.

With the optimized conditions in hand for the synthesis of 1-pyrrolines, the compatibility of various cyclopropanols was inspected (Scheme 1). Common functional groups on the phenyl ring, including halides (3b–3d), ester (3f), ether (3j), were compatible under the reaction conditions. Regardless of the presence of electron-withdrawing or -donating substituents at the *para*-position of this phenyl ring, the reactions readily proceeded with generally high yields (3b–3j). The cyclopropanol (1k) with an *ortho*-methyl substituent underwent a cyclization reaction with excellent yield, demonstrating that steric effects had little effect on product of the reaction (3k). By replacing the phenyl group with a naphthyl or thienyl group, the corresponding products (3l and 3m) were produced with slightly lower yields. When 2-substituted cyclopropanols were utilized, these reactions gave rise to a portfolio of trisubstituted 1-pyrrolines (3n–3u).The relative configuration of 3u was determined by comparison with a reported structure.^8^ Remarkably, this protocol provided a convenient method for the construction of an N-containing spiro skeleton (3t). The reaction with alkyl cyclopropanols could also furnish the desired products (3v–3x) smoothly and with good yields.

**Scheme 1:**
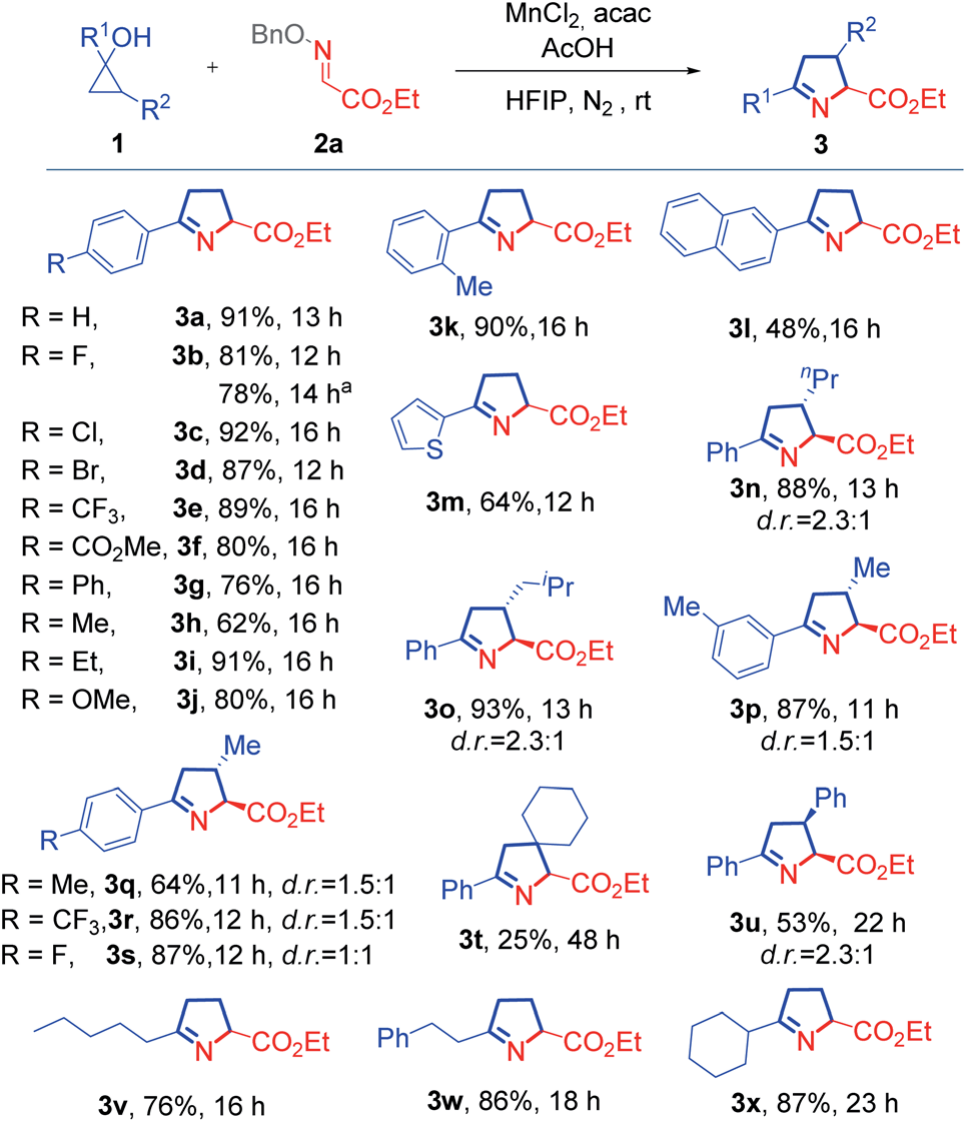
Scope of cyclopropanols. Reaction conditions: 1 (0.3 mmol), 2a (0.2 mmol), AcOH (0.2 mmol), MnCl_2_ (0.04 mmol), and acac (0.2 mmol) in HFIP (3.0 mL), at rt under N_2_. The d.r*.* values were determined by ^1^H NMR analysis with crude reaction mixture, and major isomers are shown with relative configurations. ^a^The reaction is scaled up for 10 times.

Next, we studied the scope of oxime ethers (Scheme 2). Steric hindrance from the ester moiety in the oxime ethers appeared not to influence the reaction outcome. Oxime ethers bearing various esters, such as phenyl (3y), biphenyl (3z and 3ab), 2-naphthyl (3aa), 2,4-di-*tert*-butylphenyl (3ac and 3ad), and 2,6-dimethylphenyl (3ae) esters all reacted smoothly. In addition, the substrate with *tert*-butyl ester also readily underwent cyclization to afford the desired product 3af with excellent yield. Remarkably, the trifluoromethyl-substituted pyrroline (3ag) was afforded almost quantitatively from the corresponding ketoxime ether. However, if the trifluoromethyl group was replaced by a methyl or phenyl group, the reaction failed to give rise to the desired product (3ah or 3ai), and this might be attributed to poorer electrophilic nature of the methyl or phenyl substituted substrate.

**Scheme 2:**
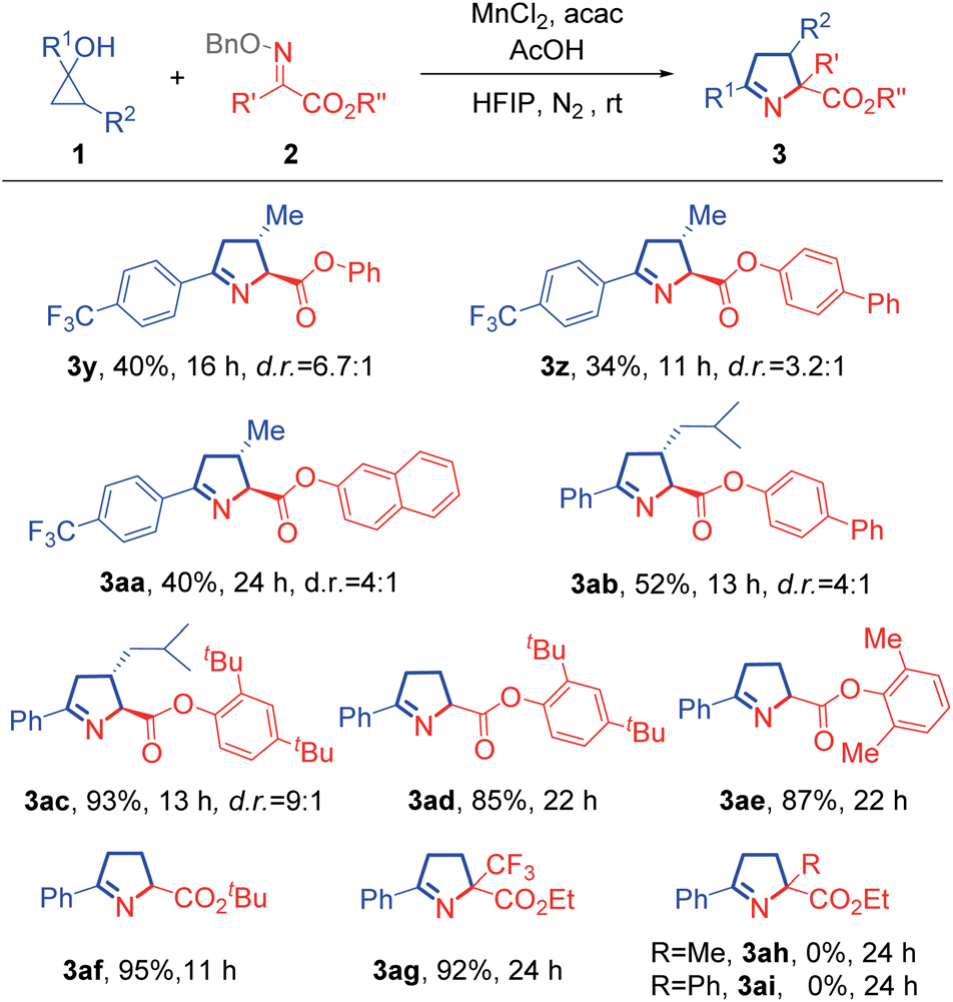
Scope of oxime ethers. Reaction conditions: 1 (0.3 mmol), 2 (0.2 mmol), AcOH (0.2 mmol), MnCl_2_ (0.04 mmol), and acac (0.2 mmol) in HFIP (3.0 mL), at rt under N_2_. The d.r. values were determined by ^1^H NMR analysis with crude reaction mixture, and major isomers are shown with relative configurations.

To illustrate the utility of this protocol, we carried out a set of synthetic applications using 1-pyrroline (3a) (Scheme 3). Upon treatment with acetyl chloride and pyridine at 42 °C, 1-pyrroline (3a) could be readily converted into the acyclic amino acid derivative (4). The reaction between 3a and LiAlH_4_ gave rise smoothly to the corresponding alcohol (5). In the presence of 2,3-dichloro-5,6-dicyano-1,4-benzoquin-4-one (DDQ) and triethylamine, the 2,5-disubstituted pyrrole (6) was obtained. Moreover, treatment of 3a with MeOTf and NaBH_4_ delivered the *N*-methyl proline derivative (7).^9^

**Scheme 3:**
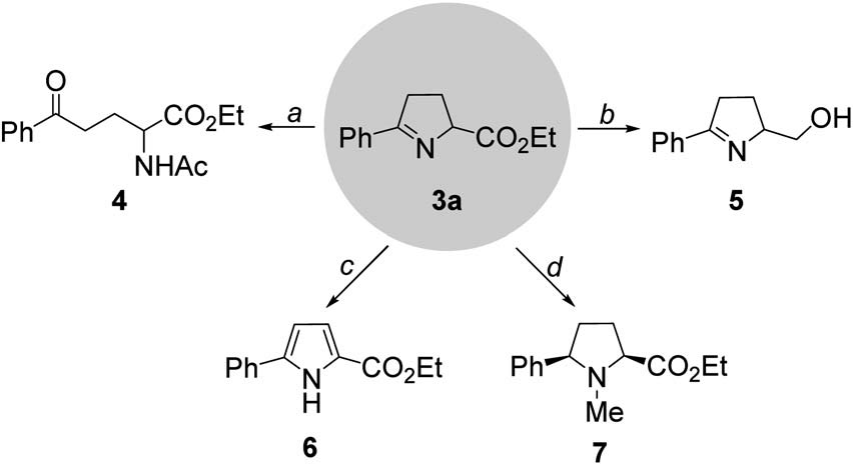
Synthetic applications. Reaction conditions: (a) AcCl, pyridine, dry DCE, 42 °C, 63% yield; (b) LiAlH_4_, THF, reflux, 90% yield; (c) DDQ, Et_3_N, DCM, rt, 53% yield; (d) MeOTf, DCM, and then NaBH_4_, THF, 40% yield, *cis* : *trans* = 6.6 : 1.

To probe the mechanistic pathways, we performed a radical trapping experiment in the presence of 2.0 equiv. of radical scavenger TEMPO. The radical trapping product (8) was detected by high-resolution mass spectrometry (HRMS) (Scheme 4A, top). In addition, the reaction was obviously suppressed when 1,1-diphenylethylene was added under standard condition (Scheme 4A, bottom). These results suggested that this process engaged in a radical pathway. Kinetic studies illustrated that the reaction immediately started with 20 mol% Mn(acac)_2_ but an approximate 15 min of induction period was appeared by using Mn(acac)_3_, which probably indicated that the reaction was initiated with Mn(ii) rather than Mn(iii), and the Mn(ii)/Mn(i) cycle might be involved in the transformation (Scheme 4B, for details see ESI†).

**Scheme 4:**
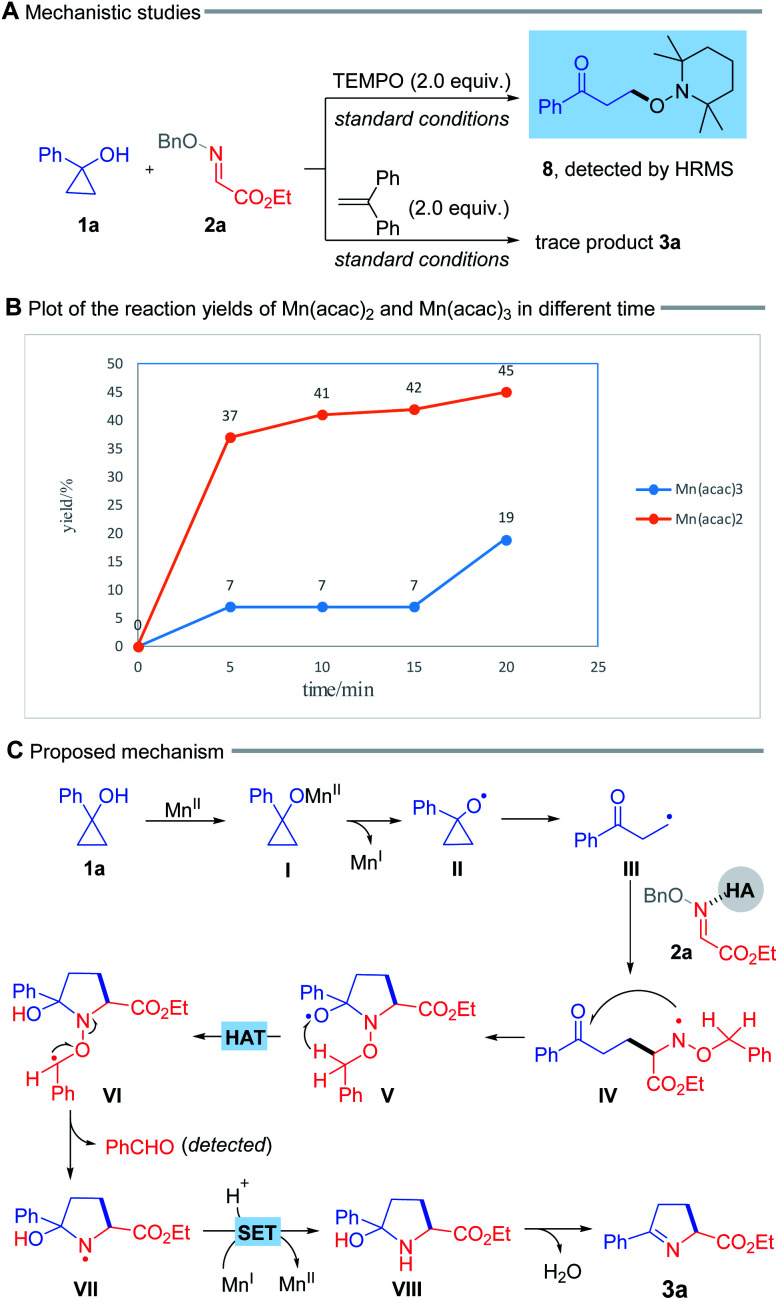
(A and B) Mechanistic studies, and (C) proposed mechanism.

On the basis of these results, a plausible mechanism for this radical process was proposed in Scheme 4C. Initially, the interaction between cyclopropanol (1a) and Mn(ii) salt gives rise to the alkoxy manganese species (I), which undergoes a ligand-to-metal charge transfer (LMCT) process, leading to the alkoxy radical (II).^5*f*^ Subsequent ring-opening of the alkoxyl radical (II) provides the alkyl radical (III). The addition of intermediate (III) to the oxime ether, possibly activated by HFIP or HOAc, furnishes the N-centered radical (IV), which intramolecularly attacks the ketone to afford a new alkoxy radical (V).^10^ The subsequent 1,5-hydrogen atom transfer (HAT) process delivers the alkyl radical (VI) at the α-position adjacent to the O atom, thus driving N–O bond cleavage to generate the N-centered radical (VII),^5*b*,11^ and benzaldehyde which was detected by TLC. This radical intermediate (VII) undergoes a single electron transfer (SET) mediated by the reduced-state Mn(i) species, and protonation to yield the cyclic pyrrolidine (VIII). Finally, dehydration of this intermediate produces 1-pyrroline (3a).

## Conclusions

In conclusion, we have developed a novel Mn-catalyzed redox-neutral reaction, producing 1-pyrrolines under mild conditions. This method features a controlled radical cascade, good tolerance of functional groups, and a broad scope of cyclopropanols and oxime ethers. Mechanistic studies have implied the presence of alkyl radicals from ring-opening of cyclopropanols and a process involving HAT and N–O bond cleavage. The role of the oxime ether as an internal oxidant and H-donor is vital to the redox-neutral reaction. This protocol provides a versatile platform to further synthesis of N-containing heterocycles based on a strategy of combining manganese catalysis and ring-opening of cycloalkanols.

## Data availability

Data for this work, including experimental procedures and characterization data for all new compounds are provided in the ESI.†

## Author contributions

C. Z. and X. W. conceived of and directed the project, T. F., C. L., and Z. W. conducted the experiments and collected and analyzed the data, C. Z. and X. W. wrote the manuscript.

## Conflicts of interest

There are no conflicts to declare.

## Supplementary Material

SC-013-D2SC00015F-s001
