# Supplementary material for: Redox-neutral manganese-catalyzed synthesis of 1-pyrrolines
Source: Chem Sci. 2022 Feb 9;13(9):2669–73. doi: 10.1039/d2sc00015f (PMC8890122; doi:10.1039/d2sc00015f)
Supplement: SC-013-D2SC00015F-s001 [file SC-013-D2SC00015F-s001.pdf]

Electronic Supplementary Material (ESI) for Chemical Science.
This journal is © The Royal Society of Chemistry 2022

Supporting Information

Redox-neutral Manganese-Catalyzed Synthesis of 1-Pyrrolines

Tingting Feng,[a] Canxiang Liu,[a] Zhen Wu,[a] Xinxin Wu,*[a] and Chen Zhu*[ab]

[a]Key Laboratory of Organic Synthesis of Jiangsu Province, College of Chemistry, Chemical Engineering and Materials Science, Soochow University, 199 Ren-Ai Road, Suzhou, Jiangsu 215123, People's Republic of China

[b] Frontiers Science Center for Transformative Molecules, Shanghai Jiao Tong University, 800 Dongchuan Road, Shanghai 200240, People's Republic of China

Table of Contents

1. General experimental details S2
2. General procedure for synthesis of cyclopropanols S2
3. General procedure for synthesis of oxime ethers S3
4. General procedure for synthesis of pyrroline products S5
5. Gram-scale reaction S15
6. Transformation of compound **3a** S15
7. Mechanistic studies S18
8. References S21
9. ^{1}H, ^{13}C, and ^{19}F NMR Spectra S22

1. General experimental details

All reactions were maintained under a nitrogen atmosphere unless otherwise stated. Commercially available reagents were used without further purification. Infrared (FT-IR) spectra were recorded on a BRUKER VERTEX 70, νmax in cm^{-1}. ^{1}H-NMR spectra were recorded on a BRUKER AVANCE III HD (400 MHz) spectrometer. Chemical shifts are reported in ppm from tetramethylsilane with the solvent resonance as internal standard ($CDCl_3$: δ 7.26). Data are reported as follows: chemical shift, multiplicity (s = singlet, d = doublet, t = triplet, q = quadruplet, br = broad, m = multiplet), coupling constants (Hz) and integration. ^{13}C-NMR spectra were recorded on a BRUKER AVANCE III HD (100 MHz) spectrometer with complete proton decoupling. Chemical shifts are reported in ppm from tetramethylsilane with the solvent resonance as the internal standard ($CDCl_3$: δ 77.16). ^{19}F-NMR spectra were recorded on a BRUKER AVANCE III HD (376 MHz) spectrometer. Mass spectra were measured with an Agilent Technologies 6120 Quadrupole LC/MS. High resolution mass spectrometry (HRMS) were measured with a GCT Premier™ and BRUKER micrOTF-Q III. Melting points were measured using INESA WRR and values are uncorrected.

2. General procedure for synthesis of cyclopropanols

The starting material cyclopropanols were prepared according to the reported procedure[1]. Cyclopropanols **1a-n**, **1p**, **1q**, **1s**, and **1u-1v** are known compounds. The spectrum data of cyclopropanols **1a-b**, **1f-h**, **1l**, **1n**, **1p-1q**, **1s**, and **1v** can be found in Ref. 1a. The spectrum data of **1e**, **1j**, and **1m** can be found in Ref. 1b. The spectrum data of **1I** can be found in Ref. 1c. The spectrum data of **1k** can be found in Ref. 1d. The spectrum data of **1u** can be found in Ref. 1e. The spectrum data of **1w and 1x** can be found in Ref. 1f.

Other cyclopropanols were prepared according to the following procedures:

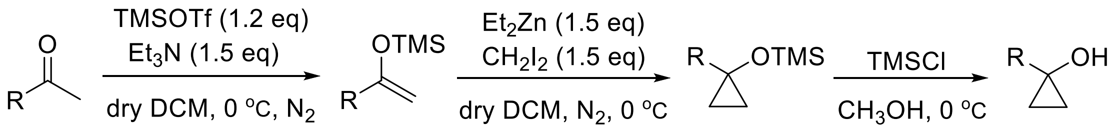

Step one: Ketone (5 mmol, 1.0 equiv.) was added to a 100 mL flame-dried round-bottomed flask at 0 °C under nitrogen. Anhydrous DCM (20 mL) was added to the flask, followed by adding Et_3N (7.5 mmol, 1.5 equiv.). Then TMSOTf (6 mmol, 1.2 equiv.) was added dropwise via syringe over 10 min. The reaction was stirred overnight, which was monitored by TLC. Upon completion, the reaction was quenched with sat. $NaHCO_3$, diluted with DCM. The aqueous layer was extracted with DCM (3 x 15 ml). The combined organic layer was washed with brine, dried over Na_2SO_4, and concentrated in vacuum. The enol ether was used in the next step without further purification.

Step two: The enol ether was transferred to a 100 mL flame-dried round-bottomed flask under nitrogen. The flask was charged with anhydrous DCM (30 mL) and diiodomethane (7.5 mmol, 1.5 equiv.). The mixture was cooled to 0 °C, then neat diethyl zinc (7.5 mmol, 1M in toluene, 1.5 equiv.) was added dropwise to the reaction solution via syringe. The mixture was warmed to room temperature and stirred for 16 h. The reaction was quenched with a sat. NH_4Cl at 0 °C. The layers were separated and the aqueous layer was extracted with DCM (3 x 15 ml). The combined organic layer was washed with brine, dried over Na_2SO_4, and concentrated in vacuum. The crude TMS

ether was also used in the next step without further purification.

Step three: The crude TMS ether was transferred to a 100 mL flame-dried round-bottomed flask under nitrogen. The flask was charged with methanol (30 mL). A single drop of chlorotrimethylsilane was added via syringe at 0 °C, and the reaction was monitored by TLC. The reaction was completed in about 1 h, then the mixture was concentrated to dryness in vacuum and the residue was purified by flash column chromatography on silica gel. The following cyclopropanol products were synthesized using the procedure described above:

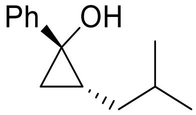

1o: white solid, m.p. 78-79 °C. Purification by flash column chromatography on silica gel (eluent: EtOAc/Petroleum ether = 1/20). ^{1}H NMR (400 MHz, $CDCl_3$) δ 7.36-7.28 (m, 4H), 7.24-7.18 (m, 1H), 2.11 (br, 1H), 1.80-1.69 (m, 1H), 1.64-1.49 (m, 2H), 1.27-1.14 (m, 2H), 0.98 (d, *J* = 6.8 Hz, 3H), 0.96 (d, *J* = 6.4 Hz, 3H), 0.88-0.84 (m, 1H); ^{13}C NMR (100 MHz, $CDCl_3$) δ 145.5, 128.3, 126.1, 124.0, 59.2, 36.8, 28.7, 27.6, 23.0, 22.7, 22.5. FT-IR: ν (cm^{-1}) 2953, 2868, 1602, 1496, 1467, 1448, 1382. HRMS [ESI] calcd for $C_{13}H_{18}ONa$ $[M+Na]^+$ 213.1250, found 213.1256.

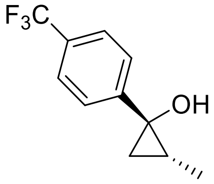

1r: white solid, m.p. 79-80 °C. Purification by flash column chromatography on silica gel (eluent: EtOAc/Petroleum ether = 1/20). ^{1}H NMR (400 MHz, $CDCl_3$) δ 7.59-7.54 (m, 2H), 7.38-7.33 (m, 2H), 2.15 (br, 1H), 1.36-1.32 (m, 3H), 1.31-1.25 (m, 2H), 0.96-0.89 (m, 1H); ^{13}C NMR (100 MHz, $CDCl_3$) δ 149.9, 128.1 (q, J_{C-F} = 32.1 Hz), 125.2 (q, J_{C-F} = 3.7 Hz), 124.3 (q, J_{C-F} = 270.0 Hz), 123.7, 58.7, 25.3, 24.5, 12.5; ^{19}F NMR (376 MHz, $CDCl_3$) δ -62.3 (s). FT-IR: ν (cm^{-1}) 3341, 1621, 1455, 1410, 1329, 1305, 1243. HRMS [EI] calcd for $C_{11}H_{11}F_3O$ $[M]^+$ 216.0757, found 216.0759.

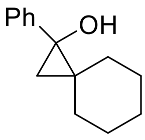

1t: white solid, m.p. 53-54 °C. Purification by flash column chromatography on silica gel (eluent: EtOAc/Petroleum ether = 1/20). ^{1}H NMR (400 MHz, $CDCl_3$) δ 7.43-7.38 (m, 2H), 7.36-7.31 (m, 2H), 7.28-7.23 (m, 1H), 2.26 (br, 1H), 1.86-1.57 (m, 4H), 1.53-1.39 (m, 2H), 1.34-1.24 (m, 2H), 1.07 (d, *J* = 5.6 Hz, 1H), 1.08-0.98 (m, 1H), 0.93-0.85 (m, 1H), 0.70 (d, *J* = 5.6 Hz, 1H); ^{13}C NMR (100 MHz, $CDCl_3$) δ 141.4, 128.2, 128.0, 127.1, 65.2, 32.6, 30.8, 30.3, 26.4, 26.1, 24.9, 23.0. FT-IR: ν (cm^{-1}) 3423, 2927, 2849, 1655, 1602, 1496, 1448. HRMS [EI] calcd for $C_{14}H_{18}O$ $[M]^+$ 202.1352, found 202.1362.

3. General procedure for synthesis of oxime ethers

Method A

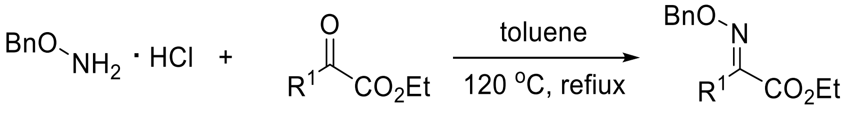

O-Benzylhydroxylamine hydrochloride (1.0 equiv.) was dissolved in toluene, glyoxylic acid ester (1.0 equiv.) was then added. The mixture was heated at refluxing temperature, until the material was consumed. The crude mixture was quenched by sat. $NaHCO_3$. The resulting aqueous phase was extracted with CH_2Cl_2. The combined organic extracts were washed with brine, dried over $MgSO_4$, filtered, and concentrated in vacuum. The residue was purified by column

chromatography on silica gel.

Method B

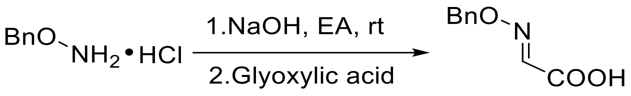

First step: O-Benzylhydroxylamine hydrochloride (1.0 equiv.) and NaOH (1.0 equiv.) was dissolved in EtOAc, glyoxylic acid (1.0 equiv.) was then added. The solution was allowed to warm to room temperature over 3 h. The reaction progress was monitored by TLC. The crude mixture was washed with brine, dried over $MgSO_4$, filtered, and concentrated in vacuum. The residue was used in the next step without further purification.

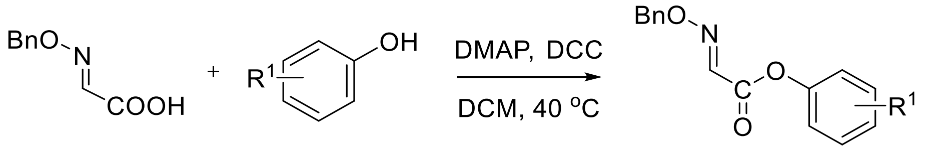

Second step: To a stirred solution of *trans*-2-((benzyloxy)imino)acetic acid (2.4 equiv.) and DCC (dicyclohexylcarbodiimide) (2 equiv.) in DCM (15 mL) were added DMAP (4-dimethylaminepyridine) (0.15 equiv.) and phenol (1.0 equiv.). The reaction mixture was stirred at 40 ^{o}C until the material was consumed. After filtration, the filtrate was concentrated in vacuum and the residue was purified by flash column chromatography on silica gel to afford the product.

Oxime ethers **2b-f** were synthesized according to **Method B**. Oxime ether **2g** was synthesized according to **Method A.** Oxime ethers **2a** and others are known compounds, the spectrum data can be found in Ref. 2.

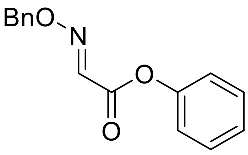

2b: white solid, m.p. 60-61 ^{o}C. Purification by flash column chromatography on silica gel (eluent: EtOAc/Petroleum ether = 1/20). ^{1}H NMR (400 MHz, $CDCl_3$) δ 7.72 (s, 1H), 7.44-7.36 (m, 7H), 7.29-7.23 (m, 1H), 7.18-7.14 (m, 2H), 5.36 (s, 2H); ^{13}C NMR (100 MHz, $CDCl_3$) δ 160.4, 150.2, 140.6, 135.8, 129.6, 128.7, 128.7, 128.6, 126.3, 121.4, 78.5. FT-IR: ν (cm^{-1}) 2963, 1763, 1600, 1589, 1484, 1455, 1363. HRMS [ESI] calcd for $C_{15}H_{14}NO_3$ $[M+H]^+$ 256.0968, found 256.0967.

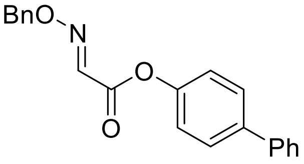

2c: yellow solid, m.p. 77-78 ^{o}C. Purification by flash column chromatography on silica gel (eluent: EtOAc/Petroleum ether = 1/20). ^{1}H NMR (400 MHz, $CDCl_3$) δ 7.78 (s, 1H), 7.66-7.58 (m, 4H), 7.50-7.36 (m, 8H), 7.30-7.25 (m, 2H), 5.41 (s, 2H); ^{13}C NMR (100 MHz, $CDCl_3$) δ 160.5, 149.6, 140.6, 140.2, 139.5, 135.8, 128.8, 128.7, 128.7, 128.6, 128.3, 127.5, 127.1, 121.7, 78.5. FT-IR: ν (cm^{-1}) 2970, 1746, 1597, 1518, 1487, 1454, 1370. HRMS [ESI] calcd for $C_{21}H_{18}NO_3$ $[M+H]^+$ 332.1281, found 332.1278.

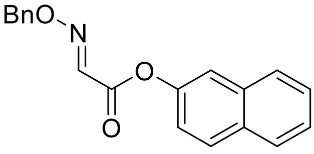

2d: yellow oil. Purification by flash column chromatography on silica gel (eluent: EtOAc/Petroleum ether = 1/20). ^{1}H NMR (400

MHz, $CDCl_3$) δ 7.96-7.89 (m, 2H), 7.89 (s, 1H), 7.80 (d, *J* = 8.4 Hz, 1H), 7.57-7.52 (m, 2H), 7.51-7.37 (m, 7H), 5.44 (s, 2H); ^{13}C NMR (100 MHz, $CDCl_3$) δ 160.5, 146.1, 140.5, 135.9, 134.7, 128.9, 128.7, 128.1, 126.7, 126.7, 126.6, 126.5, 125.4, 121.2, 118.0, 78.6. FT-IR: ν (cm^{-1}) 2970, 1751, 1595, 1509, 1495, 1453, 1391. HRMS [ESI] calcd for $C_{19}H_{16}NO_3$ $[M+H]^+$ 306.1125, found 306.1117.

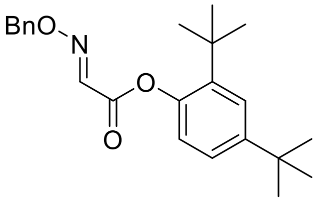

2e: white solid, m.p. 60-61 °C. Purification by flash column chromatography on silica gel (eluent: EtOAc/Petroleum ether = 1/20). ^1H NMR (400 MHz, $CDCl_3$) δ 7.73 (s, 1H), 7.44-7.35 (m, 6H), 7.24 (dd, *J* = 8.4, 2.4 Hz, 1H), 7.01 (d, *J* = 8.8 Hz, 1H), 5.35 (s, 2H), 1.35 (s, 9H), 1.32 (s, 9H); ^{13}C NMR (100 MHz, $CDCl_3$) δ 160.7, 148.6, 146.2, 140.8, 140.1, 135.9, 128.9, 128.6, 128.6, 124.2, 123.8, 122.8, 78.4, 34.7, 31.5, 30.2. FT-IR: ν (cm^{-1}) 2961, 1732, 1594, 1495, 1455, 1397, 1364. HRMS [EI] calcd for $C_{23}H_{29}NO_3$ $[M]^+$ 367.2142, found 367.2148.

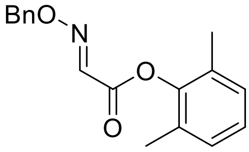

2f: white solid, m.p. 90-91 °C. Purification by flash column chromatography on silica gel (eluent: EtOAc/Petroleum ether = 1/20). ^1H NMR (400 MHz, $CDCl_3$) δ 7.77 (s, 1H), 7.46-7.34 (m, 5H), 7.08 (br, 3H), 5.38 (s, 2H), 2.18 (s, 6H); ^{13}C NMR (100 MHz, $CDCl_3$) δ 159.7, 147.7, 140.2, 135.7, 130.1, 128.8, 128.7, 128.6, 126.3, 78.5, 16.3. FT-IR: ν (cm^{-1}) 2967, 1792, 1594, 1497, 1475, 1454, 1373. HRMS [ESI] calcd for $C_{17}H_{17}NO_3Na$ $[M+Na]^+$ 306.1101, found 306.1106.

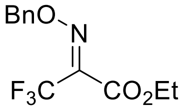

2g: Z/E ratio = 1:2.3, yellow oil. Purification by flash column chromatography on silica gel (eluent: EtOAc/Petroleum ether = 1/20). ^1H NMR (400 MHz, $CDCl_3$) δ 7.42-7.32 (m, 5H, two isomers), 5.39 (s, 0.6H, one isomer), 5.30 (s, 1.4H, one isomer), 4.40-4.32 (m, 2H, two isomers), 1.39-1.30 (m, 3H, two isomers); ^{13}C NMR (100 MHz, $CDCl_3$) δ 157.3 (overlap, two isomers), 140.2 (q, J_{C-F} = 35.4 Hz) (overlap, two isomers), 135.4 (overlap, two isomers), 128.6 (overlap, two isomers), 128.6 (overlap, two isomers), 128.4 (overlap, two isomers), 118.9 (q, J_{C-F} = 272.4 Hz) (overlap, two isomers), 79.1 (overlap, two isomers), 62.8 (overlap, two isomers), 13.9 (overlap, two isomers); ^{19}F NMR (376 MHz, $CDCl_3$) δ -63.6 (s) & -65.8 (s) (two isomers). FT-IR: ν (cm^{-1}) 2988, 1746, 1629, 1499, 1456, 1371, 1344, 1258. HRMS [EI] calcd for $C_{12}H_{12}F_3NO_3$ $[M]^+$ 275.0764, found 275.0761.

4. General procedure for synthesis of products

Cyclopropanol **1** (0.3 mmol, 1.5 equiv.), oxime ether **2** (0.2 mmol, 1.0 equiv.), and $MnCl_2$ (0.04 mmol, 20 mol %) were loaded in a flame-dried reaction vial, which was subjected to evacuation/ flushing with nitrogen three times. HFIP (3.0 mL) was added to the mixture via syringe. Then acetylacetone (acac, 0.2 mmol, 1.0 equiv.) and AcOH (0.2 mmol, 1.0 equiv.) were added to the mixture, which was stirred at room temperature until the starting material had been consumed as determined by TLC. The mixture was quenched with H_2O. The aqueous layer was extracted with DCM. The organic layer was washed with brine, dried over Na_2SO_4, concentrated in vacuum, and purified by flash column chromatography on silica gel (eluent: EtOAc/Petroleum ether) to give the corresponding products.

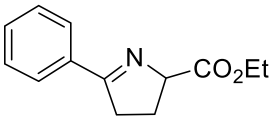

3a: 39.5 mg, 91% yield, yellow oil. Purification by flash column chromatography on silica gel (eluent: EtOAc/Petroleum ether = 1/8). ^{1}H NMR (400 MHz, $CDCl_3$) δ 7.89-7.84 (m, 2H), 7.47-7.36 (m, 3H), 4.92-4.85 (m, 1H), 4.23 (q, J = 7.2 Hz, 2H), 3.20-3.09 (m, 1H), 3.02-2.91 (m, 1H), 2.39-2.27 (m, 1H), 2.27-2.16 (m, 1H), 1.30 (t, J = 7.2 Hz, 3H); ^{13}C NMR (100 MHz, $CDCl_3$) δ 176.1, 173.0, 133.9, 130.9, 128.4, 128.1, 74.7, 61.1, 35.5, 26.5, 14.2. FT-IR: ν (cm^{-1}) 2979, 2159, 1968, 1732, 1653, 1613, 1575, 1507, 1496, 1448. HRMS [ESI] calcd for $C_{13}H_{16}NO_2$ $[M+H]^+$ 218.1176, found 218.1175.

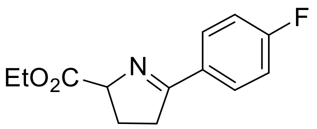

3b: 38.1 mg, 81% yield, yellow oil. Purification by flash column chromatography on silica gel (eluent: EtOAc/Petroleum ether = 1/8). ^{1}H NMR (400 MHz, $CDCl_3$) δ 7.91-7.84 (m, 2H), 7.13-7.04 (m, 2H), 4.92-4.85 (m, 1H), 4.23 (q, J = 7.2 Hz, 2H), 3.18-3.06 (m, 1H), 3.01-2.89 (m, 1H), 2.40-2.29 (m, 1H), 2.29-4.2.17 (m, 1H), 1.30 (t, J = 7.2 Hz, 3H); ^{13}C NMR (100 MHz, $CDCl_3$) δ 174.9, 172.9, 164.5 (d, $J_{C\text{-}F}$ = 249.8 Hz), 130.2 (d, $J_{C\text{-}F}$ = 8.5 Hz), 130.2 (d, $J_{C\text{-}F}$ = 4.1 Hz), 115.5 (d, $J_{C\text{-}F}$ = 21.6 Hz), 74.6, 61.2, 35.5, 26.5, 14.2; ^{19}F NMR (376 MHz, $CDCl_3$) δ -109.1 (s). FT-IR: ν (cm^{-1}) 2974, 2893, 2159, 1976, 1735, 1653, 1636, 1603, 1513, 1457. HRMS [ESI] calcd for $C_{13}H_{14}FNO_2Na$ $[M+Na]^+$ 258.0901, found 258.0903.

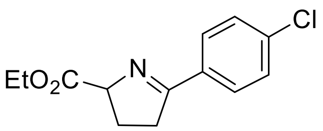

3c: 46.2 mg, 92% yield, yellow oil. Purification by flash column chromatography on silica gel (eluent: EtOAc/Petroleum ether = 1/8). ^{1}H NMR (400 MHz, $CDCl_3$) δ 7.84-7.78 (m, 2H), 7.40-7.35 (m, 2H), 4.92-4.85 (m, 1H), 4.24 (q, J = 7.2 Hz, 2H), 3.17-3.06 (m, 1H), 3.00-2.89 (m, 1H), 2.41-2.29 (m, 1H), 2.29-2.18 (m, 1H), 1.31 (t, J = 7.2 Hz, 3H); ^{13}C NMR (100 MHz, $CDCl_3$) δ 174.9, 172.8, 137.1, 132.4, 129.4, 128.7, 74.7, 61.2, 35.4, 26.5, 14.2. FT-IR: ν (cm^{-1}) 2975, 2897, 2007, 1713, 1616, 1597, 1568, 1492, 1456, 1403. HRMS [ESI] calcd for $C_{13}H_{14}ClNO_2Na$ $[M+Na]^+$ 274.0605, found 274.0594.

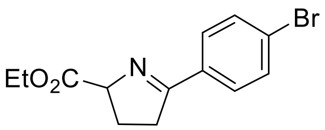

3d: 51.3 mg, 87% yield, brown oil. Purification by flash column chromatography on silica gel (eluent: EtOAc/Petroleum ether = 1/8). ^{1}H NMR (400 MHz, $CDCl_3$) δ 7.76-7.70 (m, 2H), 7.56-7.50 (m, 2H), 4.91-4.84 (m, 1H), 4.23 (q, J = 7.2 Hz, 2H), 3.16-3.06 (m, 1H), 3.00-2.89 (m, 1H), 2.40-2.29 (m, 1H), 2.29-2.17 (m, 1H), 1.30 (t, J = 7.2 Hz, 3H); ^{13}C NMR (100 MHz, $CDCl_3$) δ 175.1, 172.8, 132.7, 131.7, 129.6, 125.6, 74.7, 61.2, 35.4, 26.5, 14.2. FT-IR: ν (cm^{-1}) 2974, 2883, 2159, 1971, 1735, 1653, 1636, 1591, 1488, 1457. HRMS [ESI] calcd for $C_{13}H_{14}BrNO_2Na$ $[M+Na]^+$ 318.0100, found 318.0094.

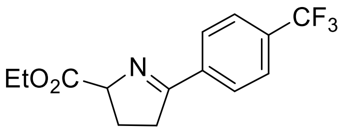

3e: 50.7 mg, 89% yield, yellow solid, m.p. 77-78 ^{o}C. Purification by flash column chromatography on silica gel (eluent: EtOAc/Petroleum ether = 1/8). ^{1}H NMR (400 MHz, $CDCl_3$) δ 8.01-7.95 (m, 2H), 7.68-7.63 (m, 2H), 4.96-4.89 (m, 1H), 4.24 (q, J = 7.2 Hz, 2H), 3.21-3.11 (m, 1H), 3.05-2.94 (m, 1H), 2.43-2.32 (m, 1H), 2.32-2.21 (m, 1H), 1.31 (t, J = 7.2 Hz, 3H); ^{13}C NMR (100 MHz, $CDCl_3$) δ 175.0, 172.6, 137.0, 132.5 (q, $J_{C\text{-}F}$ = 32.1 Hz),

128.4, 125.4 (q, $J_{C\text{-}F}$ = 3.5 Hz), 123.9 (q, $J_{C\text{-}F}$ = 271.0 Hz), 74.8, 61.3, 35.5, 26.4, 14.2; ^{19}F NMR (376 MHz, $CDCl_3$) δ -62.9 (s). FT-IR: ν (cm^{-1}) 2975, 2883, 2159, 2009, 1731, 1647, 1618, 1575, 1519, 1455. HRMS [ESI] calcd for $C_{14}H_{14}F_3NO_2Na$ $[M+Na]^+$ 308.0869, found 308.0869.

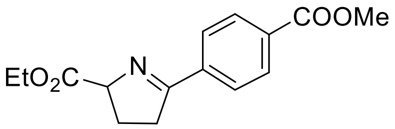

3f: 44.0 mg, 80% yield, yellow solid, m.p. 89-90 ^{o}C. Purification by flash column chromatography on silica gel (eluent: EtOAc/Petroleum ether = 1/8). ^{1}H NMR (400 MHz, $CDCl_3$) δ 8.09-8.04 (m, 2H), 7.96-7.91 (m, 2H), 4.95-4.89 (m, 1H), 4.24 (q, *J* = 7.2 Hz, 2H), 3.92 (s, 3H), 3.22-3.11 (m, 1H), 3.05-2.94 (m, 1H), 2.42-2.31 (m, 1H), 2.31-2.20 (m, 1H), 1.31 (t, *J* = 7.2 Hz, 3H); ^{13}C NMR (100 MHz, $CDCl_3$) δ 175.4, 172.7, 166.6, 137.8, 132.1, 129.7, 128.0, 74.9, 61.3, 52.3, 35.6, 26.5, 14.2. FT-IR: ν (cm^{-1}) 2985, 2949, 2850, 2159, 2030, 1735, 1653, 1636, 1507, 1441. HRMS [ESI] calcd for $C_{15}H_{17}NO_4Na$ $[M+Na]^+$ 298.1050, found 298.1044.

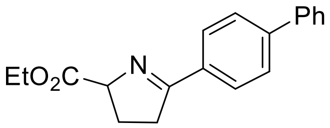

3g: 44.6 mg, 76% yield, yellow solid, m.p. 109-110 ^{o}C. Purification by flash column chromatography on silica gel (eluent: EtOAc/Petroleum ether = 1/8). ^{1}H NMR (400 MHz, $CDCl_3$) δ 7.98-7.92 (m, 2H), 7.67-7.59 (m, 4H), 7.48-7.42 (m, 2H), 7.40-7.34 (m, 1H), 4.96-4.89 (m, 1H), 4.25 (q, *J* = 7.2 Hz, 2H), 3.24-3.13 (m, 1H), 3.06-2.96 (m, 1H), 2.43-2.33 (m, 1H), 2.33-2.20 (m, 1H), 1.32 (t, *J* = 7.2 Hz, 3H); ^{13}C NMR (100 MHz, $CDCl_3$) δ 175.7, 173.0, 143.6, 140.3, 132.8, 128.9, 128.6, 127.8, 127.1, 127.1, 74.7, 61.2, 35.5, 26.5, 14.3. FT-IR: ν (cm^{-1}) 3034, 2977, 2931, 2159, 1725, 1604, 1580, 1487, 1459, 1405. HRMS [ESI] calcd for $C_{19}H_{20}NO_2$ $[M+H]^+$ 294.1489, found 294.1477.

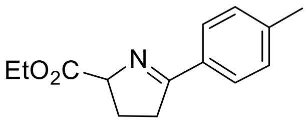

3h: 28.6 mg, 62% yield, yellow oil. Purification by flash column chromatography on silica gel (eluent: EtOAc/Petroleum ether = 1/8). ^{1}H NMR (400 MHz, $CDCl_3$) δ 7.80-7.74 (m, 2H), 7.24-7.18 (m, 2H), 4.91-4.85 (m, 1H), 4.23 (q, *J* = 7.2 Hz, 2H), 3.19-3.08 (m, 1H), 3.01-2.91 (m, 1H), 2.38 (s, 3H), 2.37-2.28 (m, 1H), 2.27-2.16 (m, 1H), 1.31 (t, *J* = 7.2 Hz, 3H); ^{13}C NMR (100 MHz, $CDCl_3$) δ 175.9, 173.1, 141.2, 131.2, 129.1, 128.0, 74.6, 61.1, 35.4, 26.5, 21.5, 14.2. FT-IR: ν (cm^{-1}) 2982, 2919, 2156, 2022, 1747, 1713, 1614, 1564, 1509, 1445, 1427. HRMS [ESI] calcd for $C_{14}H_{18}NO_2$ $[M+H]^+$ 232.1332, found 232.1322.

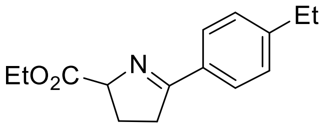

3i: 44.6 mg, 91% yield, yellow oil. Purification by flash column chromatography on silica gel (eluent: EtOAc/Petroleum ether = 1/8). ^{1}H NMR (400 MHz, $CDCl_3$) δ 7.82-7.76 (m, 2H), 7.25-7.20 (m, 2H), 4.91-4.84 (m, 1H), 4.22 (q, *J* = 7.2 Hz, 2H), 3.18-3.08 (m, 1H), 3.01-2.90 (m, 1H), 2.67 (q, *J* = 7.6 Hz, 2H), 2.38-2.26 (m, 1H), 2.26-2.15 (m, 1H), 1.30 (t, *J* = 7.2 Hz, 3H), 1.23 (t, *J* = 7.6 Hz, 3H); ^{13}C NMR (100 MHz, $CDCl_3$) δ 175.9, 173.1, 147.5, 131.4, 128.2, 127.9, 74.6, 61.1, 35.4, 28.8, 26.5, 15.4, 14.2. FT-IR: ν (cm^{-1}) 2970, 2934, 2874, 2159, 2024, 1734, 1653, 1610, 1509, 1457. HRMS [ESI] calcd for $C_{15}H_{19}NO_2Na$ $[M+Na]^+$ 268.1308, found 268.1299.

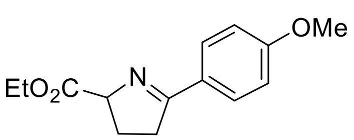

3j: 39.5 mg, 80% yield, yellow solid, m.p. 40-41 °C. Purification by flash column chromatography on silica gel (eluent: EtOAc/Petroleum ether = 1/8). ^{1}H NMR (400 MHz, $CDCl_3$) δ 7.85-7.80 (m, 2H), 6.93-6.88 (m, 2H), 4.89-4.82 (m, 1H), 4.22 (q, *J* = 7.2 Hz, 2H), 3.83 (s, 3H), 3.16-3.06 (m, 1H), 2.99-2.89 (m, 1H), 2.38-2.27 (m, 1H), 2.25-2.14 (m, 1H), 1.30 (t, *J* = 7.2 Hz, 3H); ^{13}C NMR (100 MHz, $CDCl_3$) δ 175.4, 173.2, 161.8, 129.8, 126.6, 113.7, 74.4, 61.1, 55.3, 35.4, 26.5, 14.2. FT-IR: ν (cm^{-1}) 3021, 2975, 2927, 2159, 2040, 1736, 1653, 1636, 1514, 1456. HRMS [ESI] calcd for $C_{14}H_{17}NO_3Na$ $[M+Na]^+$ 270.1101, found 270.1090.

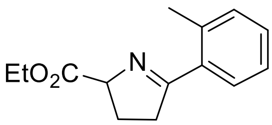

3k: 41.6 mg, 90% yield, yellow oil. Purification by flash column chromatography on silica gel (eluent: EtOAc/Petroleum ether = 1/8). ^{1}H NMR (400 MHz, $CDCl_3$) δ 7.43 (d, *J* = 7.6 Hz, 1H), 7.30-7.17 (m, 3H), 4.97-4.90 (m, 1H), 4.23 (q, *J* = 7.2 Hz, 2H), 3.12-2.92 (m, 2H), 2.52 (s, 3H), 2.37-2.25 (m, 1H), 2.24-2.13 (m, 1H), 1.30 (t, *J* = 7.2 Hz, 3H); ^{13}C NMR (100 MHz, $CDCl_3$) δ 178.1, 173.0, 137.5, 134.3, 131.3, 129.4, 128.9, 125.5, 75.1, 61.0, 38.8, 26.5, 21.4, 14.2. FT-IR: ν (cm^{-1}) 2975, 2929, 2893, 2159, 1976, 1735, 1653, 1636, 1507, 1457. HRMS [ESI] calcd for $C_{14}H_{18}NO_2$ $[M+H]^+$ 232.1332, found 232.1322.

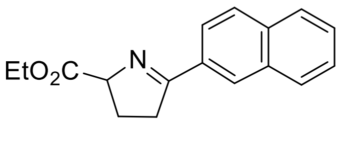

3l: 25.6 mg, 48% yield, yellow solid, m.p. 86-87 °C. Purification by flash column chromatography (eluent: EtOAc/Petroleum ether = 1/8). ^{1}H NMR (400 MHz, $CDCl_3$) δ 8.22 (s, 1H), 8.12 (dd, *J* = 8.4, 1.6 Hz, 1H), 7.91-7.83 (m, 3H), 7.56-7.48 (m, 2H), 4.99-4.93 (m, 1H), 4.26 (q, *J* = 7.2 Hz, 2H), 3.33-3.23 (m, 1H), 3.15-3.05 (m, 1H), 2.45-2.34 (m, 1H), 2.34-2.23 (m, 1H), 1.33 (t, *J* = 7.2 Hz, 3H); ^{13}C NMR (100 MHz, $CDCl_3$) δ 176.0, 173.0, 134.6, 132.9, 131.4, 128.8, 128.8, 128.2, 127.8, 127.3, 126.5, 124.8, 74.8, 61.2, 35.5, 26.6, 14.3. FT-IR: ν (cm^{-1}) 2979, 2935, 2159, 1969, 1733, 1653, 1609, 1572, 1487, 1455. HRMS [ESI] calcd for $C_{17}H_{17}NO_2Na$ $[M+Na]^+$ 290.1151, found 290.1160.

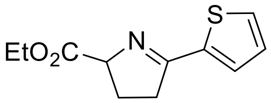

3m: 28.6 mg, 64% yield, yellow oil, Purification by flash column chromatography on silica gel (eluent: EtOAc/Petroleum ether = 1/8). ^{1}H NMR (400 MHz, $CDCl_3$) δ 7.46 (d, *J* = 5.2 Hz, 1H), 7.40 (d, *J* = 3.2 Hz, 1H), 7.12-7.05 (m, 1H), 4.91-4.83 (m, 1H), 4.23 (q, *J* = 7.2 Hz, 2H), 3.20-3.09 (m, 1H), 3.05-2.93 (m, 1H), 2.41-2.19 (m, 2H), 1.30 (t, *J* = 7.2 Hz, 3H); ^{13}C NMR (100 MHz, $CDCl_3$) δ 172.8, 170.4, 138.6, 130.2, 130.0, 127.5, 74.4, 61.1, 36.1, 26.9, 14.2. FT-IR: ν (cm^{-1}) 3068, 2978, 2920, 2850, 1734, 1660, 1632, 1608, 1522, 1469, 1454. HRMS [ESI] calcd for $C_{11}H_{14}NO_2S$ $[M+H]^+$ 224.0740, found 224.0742.

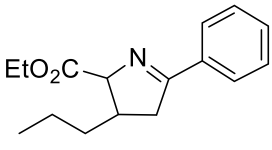

3n: *d.r.* = 1:2.3, 45.6 mg, 88% yield, yellow oil. Purification by flash column chromatography on silica gel (eluent: EtOAc/Petroleum ether = 1/8). ^{1}H NMR (400 MHz, $CDCl_3$) δ 7.90-7.84 (m, 2H, two isomers), 7.48-7.37 (m, 3H, two isomers), 4.93 (d, *J* = 8.0 Hz, 0.3H, one isomer), 4.53-4.49 (m, 0.7H, one isomer), 4.27-4.19 (m, 2H, two isomers), 3.35-3.25 (m, 0.7H, one isomer), 3.15-3.05 (m, 0.3H, one isomer), 2.84-2.56 (m, 2H, two isomers), 1.68-1.58 (m, 1H, two isomers),

1.54-1.34 (m, 3H, two isomers), 1.34-1.26 (m, 3H, two isomers), 0.98-0.90 (m, 3H, two isomers); ^{13}C NMR (100 MHz, $CDCl_3$) δ 177.0 & 175.6 (two isomers), 172.9 & 171.2 (two isomers), 134.1 & 134.0 (two isomers), 131.0 & 130.9 (two isomers), 128.5 & 128.4 (two isomers), 128.0 (overlap, two isomers), 80.0 & 76.9 (two isomers), 61.1 & 60.7 (two isomers), 41.8 & 40.5 (two isomers), 41.2 & 41.2 (two isomers), 37.2 & 32.5 (two isomers), 21.9 & 21.1 (two isomers), 14.3 & 14.2 (two isomers). 14.1 & 14.0 (two isomers). FT-IR: ν (cm^{-1}) 2974, 2929, 2160, 1977, 1735, 1653, 1636, 1577, 1507, 1457. HRMS [ESI] calcd for $C_{16}H_{21}NO_2Na$ $[M+Na]^+$ 282.1465, found 282.1454.

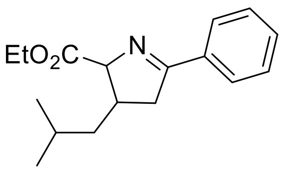

3o: *d.r.* = 1:2.3, 50.8 mg, 93% yield, yellow oil. Purification by flash column chromatography on silica gel (eluent: EtOAc/Petroleum ether = 1/8). ^{1}H NMR (400 MHz, $CDCl_3$) δ 7.89-7.84 (m, 2H, two isomers), 7.49-7.37 (m, 3H, two isomers), 4.95-4.89 (m, 0.3H, one isomer), 4.52-4.46 (m, 0.7H, one isomer), 4.29-4.16 (m, 2H, two isomers), 3.36-3.24 (m, 0.7H, one isomer), 3.15-3.04 (m, 0.3H, one isomer), 2.84-2.59 (m, 2H, two isomers), 1.74-1.60 (m, 1.3H, two isomers), 1.56-1.47 (m, 0.7H, one isomer), 1.41-1.33 (m, 1H, two isomers), 1.31 (t, J = 7.2 Hz, 3H, two isomers), 0.97-0.90 (m, 6H, two isomers); ^{13}C NMR (100 MHz, $CDCl_3$) δ 177.1 & 175.5 (two isomers), 172.9 & 171.2 (two isomers), 134.2 & 134.0 (two isomers), 130.9 & 130.9 (two isomers), 128.4 & 128.4 (two isomers), 128.0 & 127.9 (two isomers), 81.3 & 77.1 (two isomers), 61.0 & 60.7 (two isomers), 44.4 & 42.1 (two isomers), 40.6 & 39.5 (two isomers), 39.3 & 39.3 (two isomers), 27.1 & 26.6 (two isomers), 23.4 & 22.9 (two isomers), 22.3 & 21.9 (two isomers), 14.3 & 14.2 (two isomers). FT-IR: ν (cm^{-1}) 2974, 2928, 2893, 2159, 1969, 1735, 1653, 1636, 1512, 1507, 1457. HRMS [ESI] calcd for $C_{17}H_{23}NO_2Na$ $[M+Na]^+$ 296.1621, found 296.1624.

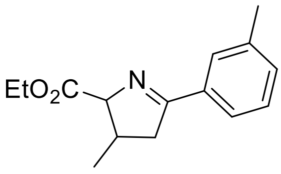

3p: *d.r.* = 1:1.5, 42.6 mg, 87% yield, yellow oil. Purification by flash column chromatography on silica gel (eluent: EtOAc/Petroleum ether = 1/8). ^{1}H NMR (400 MHz, $CDCl_3$) δ 7.78-7.73 (m, 1H, two isomers), 7.64-7.58 (m, 1H, two isomers), 7.33-7.24 (m, 2H, two isomers), 4.86 (d, J = 8.0 Hz, 0.6H, one isomer), 4.45-4.41 (m, 0.4H, one isomer), 4.32-4.15 (m, 2H, two isomers), 3.36-3.27 (m, 0.4H, one isomer), 3.17-3.08 (m, 0.6H, one isomer), 2.92-2.82 (m, 0.6H, one isomer), 2.80-2.72 (m, 0.6H, one isomer), 2.72-2.57 (m, 0.8H, two isomers), 2.38 (s, 3H, two isomers), 1.34-1.27 (m, 3H, two isomers), 1.24 (d, J = 6.4 Hz, 1.2H, one isomer), 1.05 (d, J = 6.8 Hz, 1.8H, one isomer); ^{13}C NMR (100 MHz, $CDCl_3$) δ 176.5 & 175.8 (two isomers), 172.8 & 171.3 (two isomers), 138.1 (overlap, two isomers), 134.0 & 133.9 (two isomers), 131.7 (overlap, two isomers), 128.4 & 128.4 (two isomers), 128.3 (overlap, two isomers), 125.2 & 125.2 (two isomers), 81.4 (overlap, two isomers), 61.0 & 60.7 (two isomers), 43.7 & 43.1 (two isomers), 36.2 & 35.0 (two isomers), 21.3 & 20.0 (two isomers), 15.8 (overlap, two isomers), 14.4 & 14.3 (two isomers). FT-IR: ν (cm^{-1}) 2974, 2929, 2893, 2159, 1976, 1735, 1653, 1636, 1617, 1507, 1457. HRMS [ESI] calcd for $C_{15}H_{19}NO_2Na$ $[M+Na]^+$ 268.1308, found 268.1310.

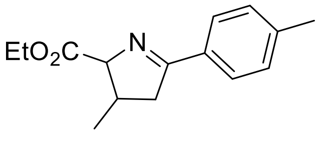

3q: *d.r.* = 1:1.5, 31.3 mg, 64% yield, yellow oil. Purification by flash column chromatography on silica gel (eluent: EtOAc/Petroleum ether = 1/8). ^{1}H NMR (400 MHz, $CDCl_3$) δ

7.78-7.72 (m, 2H, two isomers), 7.23-7.18 (m, 2H, two isomers), 4.88-4.83 (m, 0.4H, one isomer), 4.44-4.40 (m, 0.6H, one isomer), 4.32-4.15 (m, 2H, two isomers), 3.30 (ddd, J = 16.4, 8.0, 1.6 Hz, 0.6H, one isomer), 3.11 (ddd, J = 16.0, 8.0, 1.2 Hz, 0.4H, one isomer), 2.91-2.79 (m, 0.4H, one isomer), 2.78-2.70 (m, 0.4H, one isomer), 2.70-2.63 (m, 0.6H, one isomer), 2.63-2.55 (m, 0.6H, one isomer), 2.38 (s, 3H, two isomers), 1.30 (t, J = 7.2 Hz, 1.8H, one isomer), 1.29 (t, J = 7.2 Hz, 1.2H, one isomer), 1.23 (d, J = 6.8 Hz, 1.8H, one isomer), 1.05 (d, J = 6.8 Hz, 1.2H, one isomer); ^{13}C NMR (100 MHz, $CDCl_3$) δ 176.1 & 175.4 (two isomers), 172.8 & 171.4 (two isomers), 141.2 (overlap, two isomers), 131.5 & 131.4 (two isomers), 129.1 (overlap, two isomers), 128.0 & 127.9 (two isomers), 81.3 & 77.4 (two isomers), 61.0 & 60.6 (two isomers), 43.6 & 43.1 (two isomers), 36.2 & 35.0 (two isomers), 21.5 & 20.0 (two isomers), 15.8 (overlap, two isomers), 14.4 & 14.2 (two isomers). FT-IR: ν (cm^{-1}) 2974, 2928, 2893, 2159, 1969, 1735, 1653, 1636, 1512, 1507, 1457. HRMS [ESI] calcd for $C_{15}H_{19}NO_2Na$ $[M+Na]^+$ 268.1308, found 268.1304.

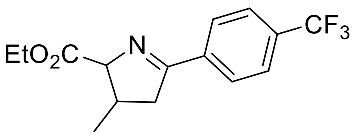

3r: *d.r.* = 1:1.5, 51.4 mg, 86% yield, yellow oil. Purification by flash column chromatography on silica gel (eluent: EtOAc/Petroleum ether = 1/8). ^{1}H NMR (400 MHz, $CDCl_3$) δ 8.00-7.95 (m, 2H, two isomers), 7.70-7.64 (m, 2H, two isomers), 4.93-4.89 (m, 0.4H, one isomer), 4.49-4.45 (m, 0.6H, one isomer), 4.32-4.17 (m, 2H, two isomers), 3.33 (ddd, J = 16.8, 8.8, 2.0 Hz, 0.6H, one isomer), 3.15 (ddd, J = 16.8, 8.4, 1.6 Hz, 0.4H, one isomer), 2.97-2.85 (m, 0.4H, one isomer), 2.81-2.74 (m, 0.4H, one isomer), 2.74-2.67 (m, 0.6H, one isomer), 2.67-2.59 (m, 0.6H, one isomer), 1.32 (t, J = 7.2 Hz, 1.8H, one isomer), 1.31 (t, J = 7.2 Hz, 1.2H, one isomer), 1.26 (d, J = 6.8 Hz, 1.8H, one isomer), 1.07 (d, J = 7.2 Hz, 1.2H, one isomer); ^{13}C NMR (100 MHz, $CDCl_3$) δ 175.2 & 174.4 (two isomers), 172.4 & 170.9 (two isomers), 137.2 & 137.2 (two isomers), 132.5 (q, J_{C-F} = 32.2 Hz) (overlap, two isomers), 128.3 & 128.3 (two isomers), 125.4 (q, J_{C-F} = 3.6 Hz) (overlap, two isomers), 123.9 (q, J_{C-F} = 270.7 Hz) (overlap, two isomers), 81.5 & 77.6 (two isomers), 61.2 & 60.8 (two isomers), 43.7 & 43.1 (two isomers), 36.3 & 35.1 (two isomers), 19.9 & 15.7 (two isomers), 14.3 & 14.2 (two isomers); ^{19}F NMR (376 MHz, $CDCl_3$) δ -62.9 (s) (overlap, two isomers). FT-IR: ν (cm^{-1}) 2973, 2927, 2881, 2161, 2023, 1743, 1713, 1618, 1413, 1374. HRMS [ESI] calcd for $C_{15}H_{17}F_3NO_2$ $[M+H]^+$ 300.1206, found 300.1207.

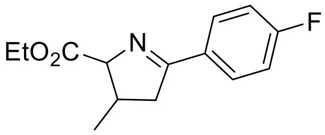

3s: *d.r.* = 1:1, 43.3 mg, 87% yield, yellow oil. Purification by flash column chromatography on silica gel (eluent: EtOAc/Petroleum ether = 1/8). ^{1}H NMR (400 MHz, $CDCl_3$) δ 7.90-7.82 (m, 2H, two isomers), 7.12-7.04 (m, 2H, two isomers), 4.88-4.83 (m, 0.5H, one isomer), 4.44-4.39 (m, 0.5H, one isomer), 4.30-4.15 (m, 2H, two isomers), 3.29 (ddd, J = 16.8, 8.8, 2.0 Hz, 0.5H, one isomer), 3.10 (ddd, J = 16.4, 8.4, 1.2 Hz, 0.5H, one isomer), 2.92-2.80 (m, 0.5H, one isomer), 2.77-2.62 (m, 1H, two isomers), 2.62-2.54 (m, 0.5H, one isomer), 1.30 (t, J = 7.2 Hz, 1.5H, one isomer), 1.29 (t, J = 7.2 Hz, 1.5H, one isomer), 1.24 (d, J = 6.8 Hz, 1.5H, one isomer), 1.05 (d, J = 6.8 Hz, 1.5H, one isomer); ^{13}C NMR (100 MHz, $CDCl_3$) δ 175.0 & 174.3 (two isomers), 172.7 & 171.2 (two isomers), 164.5 (d, J_{C-F} = 249.5 Hz) & 164.5 (d, J_{C-F} = 249.9 Hz) (two isomers), 130.4 (d, J_{C-F} = 3.1 Hz) & 130.3 (d, J_{C-F} = 3.2 Hz) (two isomers), 130.1 (d, J_{C-F} = 8.5 Hz) & 130.0 (d, J_{C-F} = 8.7 Hz) (two isomers), 115.5 (d, J_{C-F} = 21.7 Hz) (overlap, two isomers), 81.3 & 77.4 (two isomers), 61.1 & 60.7 (two isomers), 43.6 & 43.1 (two isomers), 36.4 & 35.2

(two isomers), 19.9 & 15.8 (two isomers), 14.4 & 14.2 (two isomers); ^{19}F NMR (376 MHz, $CDCl_3$) δ -109.2 (s) & -109.2 (s) (two isomers). FT-IR: ν (cm^{-1}) 2976, 2936, 2159, 1975, 1774, 1653, 1636, 1603, 1510, 1457. HRMS [EI] calcd for $C_{14}H_{16}FNO_2$ $[M]^+$ 249.1160, found 249.1159.

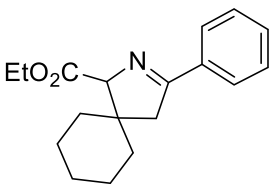

3t: 14.2 mg, 25% yield, yellow oil. Purification by flash column chromatography on silica gel (eluent: EtOAc/Petroleum ether = 1/8). ^{1}H NMR (400 MHz, $CDCl_3$) δ 7.91-7.85 (m, 2H), 7.48-7.38 (m, 3H), 4.55 (s, 1H), 4.29-4.15 (m, 2H), 2.99 (d, *J* = 16.8 Hz, 1H), 2.86 (d, *J* = 16.8 Hz, 1H), 1.72-1.47 (m, 7H), 1.46-1.35 (m, 3H), 1.30 (t, *J* = 7.2 Hz, 3H); ^{13}C NMR (100 MHz, $CDCl_3$) δ 175.7, 171.2, 134.4, 130.9, 128.4, 127.9, 83.5, 60.7, 46.7, 45.7, 37.5, 32.5, 25.8, 23.7, 23.4, 14.4. FT-IR: ν (cm^{-1}) 2970, 2929, 2894, 2159, 1976, 1734, 1653, 1636, 1576, 1507, 1456. HRMS [ESI] calcd for $C_{18}H_{23}NO_2Na$ $[M+Na]^+$ 308.1621, found 308.1628.

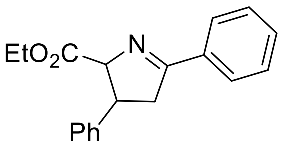

3u: *d.r.* = 1:2.3, 31.1 mg, 53% yield, yellow oil. Purification by flash column chromatography on silica gel (eluent: EtOAc/Petroleum ether = 1/8). ^{1}H NMR (400 MHz, $CDCl_3$) δ 7.99-7.95 (m, 1.4H, one isomer), 7.94-7.90 (m, 0.6H, one isomer), 7.52-7.42 (m, 3H, two isomers), 7.35-7.29 (m, 1H, two isomers), 7.25-7.22 (m, 2H, two isomers), 7.21-7.17 (m, 2H, two isomers), 5.25-5.20 (m, 0.7H, one isomer), 4.96-4.92 (m, 0.3H, one isomer), 4.29-4.21 (m, 0.6H, one isomer), 4.02 (q, *J* = 8.4 Hz, 0.7H, one isomer), 3.92-3.77 (m, 1H, two isomers), 3.74-3.62 (m, 1H, two isomers), 3.46-3.41 (m, 1.4H, one isomer), 3.18 (ddd, *J* = 17.2, 6.4, 1.2 Hz, 0.3H, one isomer), 1.30 (t, *J* = 7.2 Hz, 0.9H, one isomer), 0.85 (t, *J* = 7.2 Hz, 2.1H, one isomer); ^{13}C NMR (75 MHz, $CDCl_3$) δ 176.3 & 175.0 (two isomers), 172.2 & 170.4 (overlap, two isomers), 143.3 & 139.9 (two isomers), 133.8 & 133.6 (two isomers), 131.2 & 131.2 (two isomers), 128.9 & 128.6 (two isomers), 128.5 &128.3 (two isomers), 128.1 (overlap, two isomers), 127.8 & 127.1 (two isomers), 127.1 & 126.9 (two isomers), 82.7 & 79.2 (two isomers), 61.3 & 60.5 (two isomers), 46.6 & 46.4 (two isomers), 44.7 & 42.1 (two isomers), 14.2 & 13.7 (two isomers). FT-IR: ν (cm^{-1}) 3058, 2973, 2929, 2159, 1977, 1734, 1653, 1636, 1576, 1507, 1456. HRMS [ESI] calcd for $C_{19}H_{19}NO_2Na$ $[M+Na]^+$ 316.1308, found 316.1304.

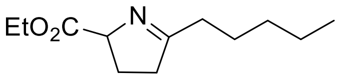

3v: 32.1 mg, 76% yield, yellow oil. Purification by flash column chromatography on silica gel (eluent: EtOAc/Petroleum ether = 1/8). ^{1}H NMR (400 MHz, $CDCl_3$) δ 4.66-4.59 (m, 1H), 4.18 (q, *J* = 7.2 Hz, 2H), 2.68-2.58 (m, 1H), 2.54-2.44 (m, 1H), 2.40-2.34 (m, 2H), 2.20-2.10 (m, 1H), 2.07-1.97 (m, 1H), 1.63-1.54 (m, 2H), 1.33-1.24 (m, 4H), 1.26 (t, *J* = 7.2 Hz, 3H), 0.89-0.84 (m, 3H); ^{13}C NMR (100 MHz, $CDCl_3$) δ 182.1, 173.2, 74.0, 60.9, 37.5, 33.8, 31.6, 26.4, 26.1, 22.4, 14.2, 14.0. FT-IR: ν (cm^{-1}) 2973, 2931, 2159, 2029, 1737, 1670, 1638, 1559, 1507, 1457. HRMS [ESI] calcd for $C_{12}H_{21}NO_2Na$ $[M+Na]^+$ 234.1465, found 234.1475.

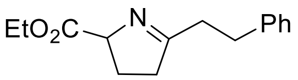

3w: 42.1 mg, 86% yield, yellow oil. Purification by flash column chromatography on silica gel (eluent: EtOAc/Petroleum ether = 1/1). ^{1}H NMR (400 MHz, $CDCl_3$) δ 7.31-7.25 (m, 2H), 7.23-7.16 (m, 3H), 4.72-4.63 (m, 1H), 4.21 (q, *J* = 7.2 Hz, 2H), 3.01-2.91 (m, 2H), 2.76-2.58 (m, 3H), 2.56-2.43 (m,

1H), 2.24-2.11 (m, 1H), 2.10-1.99 (m, 1H), 1.29 (t, *J* = 7.2 Hz, 3H); ^{13}C NMR (100 MHz, $CDCl_3$) δ 181.4, 173.0, 141.1, 128.5, 128.3, 126.1, 73.9, 61.0, 38.1, 35.3, 32.6, 26.3, 14.2. FT-IR: ν (cm^{-1}) 3027, 2980, 2957, 2930, 2872, 1734, 1640, 1604, 1454. HRMS [EI] calcd for $C_{15}H_{19}NO_2$ $[M]^+$ 245.1410, found 245.1412.

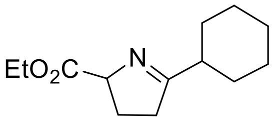

3x: 38.8 mg, 87% yield, yellow oil. Purification by flash column chromatography on silica gel (eluent: EtOAc/Petroleum ether = 1/2). ^1H NMR (400 MHz, $CDCl_3$) δ 4.66-4.58 (m, 1H), 4.17 (q, *J* = 7.2 Hz, 2H), 2.70-2.58 (m, 1H), 2.56-2.32 (m, 2H), 2.19-2.06 (m, 1H), 2.05-1.93 (m, 1H), 1.90-1.62 (m, 5H), 1.42-1.22 (m, 5H), 1.26 (t, *J* = 7.2 Hz, 3H); ^{13}C NMR (100 MHz, $CDCl_3$) δ 185.6, 173.2, 73.7, 60.9, 42.5, 35.3, 30.4, 30.4, 26.1, 26.0, 25.9, 14.2. FT-IR: ν (cm^{-1}) 2979, 2928, 2854, 1735, 1633, 1449, 1429. HRMS [EI] calcd for $C_{13}H_{21}NO_2$ $[M]^+$ 223.1567, found 223.1568.

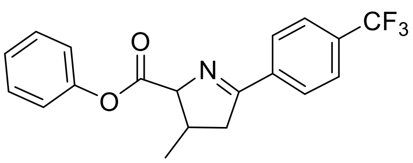

3y: *d.r.* = 1:6.7, 27.8 mg, 40% yield, yellow oil. Purification by flash column chromatography on silica gel (eluent: Acetone/Petroleum ether = 1/5). ^1H NMR (400 MHz, $CDCl_3$) δ 8.05-7.97 (m, 2H, two isomers), 7.74-7.66 (m, 2H, two isomers), 7.44-7.34 (m, 2H, two isomers), 7.25-7.21 (m, 1H, two isomers), 7.19-7.12 (m, 2H, two isomers), 5.17 (d, *J* = 8.4 Hz, 0.13H, one isomer), 4.74 (d, 0.87H, *J* = 6.0 Hz, one isomer), 3.47-3.36 (m, 0.87H, one isomer), 3.29-3.20 (m, 0.13H, one isomer), 3.13-3.01 (m, 0.13H, one isomer), 2.96-2.82 (m, 1H, two isomers), 2.78-2.68 (m, 0.87H, one isomer), 1.37 (d, *J* = 6.8 Hz, 2.61H, one isomer), 1.26 (d, *J* = 6.8 Hz, 0.39H, one isomer); ^{13}C NMR (100 MHz, $CDCl_3$) δ 175.7 & 175.0 (two isomers), 170.8 & 169.4 (two isomers), 150.6 & 150.5 (two isomers), 137.1 & 137.1 (two isomers), 132.7 (q, J_{C-F} = 32.2 Hz) (overlap, two isomers), 129.5 & 129.5 (two isomers), 128.4 & 128.3 (two isomers), 126.0 (overlap, two isomers), 125.5 (q, J_{C-F} = 3.6 Hz) (overlap, two isomers), 123.9 (q, J_{C-F} = 270.8 Hz) (overlap, two isomers), 121.5 & 121.5 (two isomers), 81.4 & 77.5 (two isomers), 43.8 & 43.3 (two isomers), 36.6 & 35.5 (two isomers), 20.0 & 16.0 (two isomers); ^{19}F NMR (376 MHz, $CDCl_3$) δ -62.9 (s) (overlap, two isomers). FT-IR: ν (cm^{-1}) 2965, 1760, 1695, 1618, 1593, 1575, 1493, 1458, 1432, 1411. HRMS [EI] calcd for $C_{19}H_{16}F_3NO_2$ $[M]^+$ 347.1128, found 347.1134.

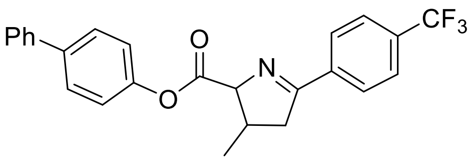

3z: *d.r.* = 1:3.2, 28.8 mg, 34% yield, yellow oil. Purification by flash column chromatography on silica gel (eluent: Acetone/Petroleum ether = 1/5). ^1H NMR (400 MHz, $CDCl_3$) δ 8.05-8.00 (m, 2H, two isomers), 7.74-7.68 (m, 2H, two isomers), 7.63-7.54 (m, 4H, two isomers), 7.47-7.41 (m, 2H, two isomers), 7.39-7.32 (m, 1H, two isomers), 7.26-7.20 (m, 2H, two isomers), 5.20 (d, *J* = 8.0 Hz, 0.24H, one isomer), 4.76 (d, *J* = 5.6 Hz, 0.76H, one isomer), 3.48-3.38 (m, 0.76H, one isomer), 3.31-3.22 (m, 0.24H, one isomer), 3.14-3.05 (m, 0.24H, one isomer), 2.97-2.83 (m, 1H, two isomers), 2.79-2.70 (m, 0.76H, one isomer), 1.39 (d, *J* = 6.8 Hz, 2.28H, one isomer), 1.29 (d, *J* = 6.8 Hz, 0.72H, one isomer); ^{13}C NMR (100 MHz, $CDCl_3$) δ 175.7 & 175.1 (two isomers), 170.9 & 169.5 (two isomers), 150.1 & 149.9 (two isomers), 140.3 (overlap, two isomers), 139.2 & 139.2 (two isomers), 137.1 & 137.0 (two isomers), 132.7 (q, J_{C-F}

= 32.1 Hz) (overlap, two isomers), 128.8 (overlap, two isomers), 128.4 & 128.3 (two isomers), 128.2 & 128.2 (two isomers), 127.4 (overlap, two isomers), 127.1 (overlap, two isomers), 125.5 (q, J_{C-F} = 3.6 Hz) (overlap, two isomers), 123.9 (q, J_{C-F} = 270.7 Hz) (overlap, two isomers), 121.8 & 121.7 (two isomers), 81.4 & 77.5 (two isomers), 43.8 & 43.3 (two isomers), 36.7 & 35.5 (two isomers), 20.0 & 16.1 (two isomers); ^{19}F NMR (376 MHz, $CDCl_3$) δ -62.3 (s) & -62.9 (s) (two isomers). FT-IR: ν (cm^{-1}) 3263, 3077, 2972, 2893, 1982, 1733, 1676, 1646, 1597, 1450. HRMS [EI] calcd for $C_{25}H_{20}F_3NO_2$ $[M]^+$ 423.1441, found 423.1440.

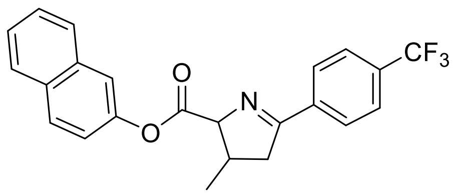

3aa: *d.r.* = 1:4, 31.8 mg, 40% yield, yellow oil. Purification by flash column chromatography on silica gel (eluent: Acetone/Petroleum ether = 1/5). ^{1}H NMR (400 MHz, $CDCl_3$) δ 8.10-8.03 (m, 2H, two isomers), 7.99-7.94 (m, 1H, two isomers), 7.91-7.84 (m, 1H, two isomers), 7.78-7.68 (m, 3H, two isomers), 7.57-7.49 (m, 3H, two isomers), 7.36-7.31 (m, 1H, two isomers), 5.35 (d, *J* = 8.0 Hz, 0.2H, one isomer), 4.93 (d, *J* = 5.6 Hz, 0.8H, one isomer), 3.53-3.42 (m, 0.8H, one isomer), 3.35-3.25 (m, 0.2H, one isomer), 3.21-3.12 (m, 0.2H, one isomer), 3.11-3.01 (m, 0.8H, one isomer), 2.97-2.88 (m, 0.2H, one isomer), 2.84-2.74 (m, 0.8H, one isomer), 1.44 (d, *J* = 6.8 Hz, 2.4H, one isomer), 1.36 (d, *J* = 7.6 Hz, 0.6H, one isomer); ^{13}C NMR (75 MHz, $CDCl_3$) δ 175.9 & 175.1 (two isomers), 170.8 & 169.4 (two isomers), 146.5 & 146.4 (two isomers), 137.2 & 137.1 (two isomers), 134.7 (overlap, two isomers), 132.7 (q, J_{C-F} = 32.5 Hz) (overlap, two isomers), 128.4 & 128.4 (two isomers), 128.1 & 128.0 (two isomers), 126.7 & 126.7 (two isomers), 126.6 (overlap, two isomers), 126.5 (overlap, two isomers), 126.2 & 126.1 (two isomers), 125.5 (q, J_{C-F} = 3.7 Hz) (overlap, two isomers), 125.4 & 125.3 (two isomers), 123.9 (q, J_{C-F} = 272.4 Hz) (overlap, two isomers), 121.3 & 121.1 (two isomers), 118.0 & 117.9 (two isomers), 81.7 & 77.8 (two isomers), 43.9 & 43.4 (two isomers), 36.5 & 35.6 (two isomers), 20.1 & 16.1 (two isomers); ^{19}F NMR (376 MHz, $CDCl_3$) δ -62.9 (s) & -63.2 (s) (two isomers). FT-IR: ν (cm^{-1}) 3065, 2964, 2930, 1760, 1617, 1600, 1575, 1509, 1460, 1410. HRMS [EI] calcd for $C_{23}H_{18}F_3NO_2$ $[M]^+$ 397.1284, found 397.1296.

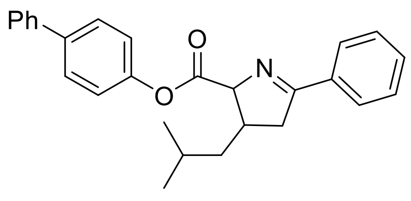

3ab: *d.r.* = 1:4, 41.3 mg, 52% yield, yellow oil. Purification by flash column chromatography on silica gel (eluent: EtOAc/Petroleum ether = 1/6). ^{1}H NMR (400 MHz, $CDCl_3$) δ 7.93-7.88 (m, 2H, two isomers), 7.61-7.53 (m, 4H, two isomers), 7.46-7.39 (m, 5H, two isomers), 7.36-7.31 (m, 1H, two isomers), 7.25-7.20 (m, 2H, two isomers), 5.21-5.17 (m, 0.2H, one isomer), 4.79-4.75 (m, 0.8H, one isomer), 3.39 (ddd, *J* = 16.8, 8.8, 1.6 Hz, 0.8H, one isomer), 3.22-3.15 (m, 0.2H, one isomer), 2.94-2.84 (m, 1H, two isomers), 2.78-2.59 (m, 1H, two isomers), 1.82-1.73 (m, 1H, two isomers), 1.69-1.60 (m, 1H, two isomers), 1.54-1.45 (m, 1H, two isomers), 1.03-0.93 (m, 6H, two isomers); ^{13}C NMR (100 MHz, $CDCl_3$) δ 177.8 & 176.2 (two isomers), 171.5 & 169.8 (two isomers), 150.2 & 150.0 (two isomers), 140.4 & 140.3 (two isomers), 139.1 & 139.1 (two isomers), 133.8 & 133.5 (two isomers), 131.2 & 131.1 (two isomers), 128.8 & 128.7 (two isomers), 128.5 & 128.4 (two isomers), 128.2 & 128.2 (two isomers), 128.1 & 128.0 (two isomers), 128.0 (overlap, two isomers), 127.4 & 127.1 (two isomers), 121.8 & 121.8 (two isomers), 80.1 & 77.0 (two isomers), 44.5 (overlap, two isomers), 42.2 & 40.7 (two isomers), 39.8

& 39.6 (two isomers), 27.1 & 26.7 (two isomers), 23.5 & 22.9 (two isomers), 22.4 & 21.9 (two isomers). FT-IR: ν (cm-1) 3060, 2955, 2870, 1759, 1683, 1613, 1576, 1516, 1449, 1428. HRMS [ESI] calcd for $C_{27}H_{28}NO_2$ $[M+H]^+$ 398.2115, found 398.2115.

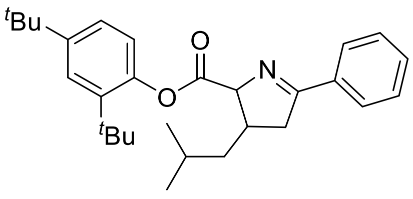

3ac: *d.r.* = 1:9, 80.6 mg, 93% yield, yellow oil. Purification by flash column chromatography on silica gel (eluent: EtOAc/Petroleum ether = 1/6). ^1H NMR (400 MHz, $CDCl_3$) δ 7.95-7.89 (m, 2H, two isomers), 7.49-7.40 (m, 4H, two isomers), 7.26-7.22 (m, 1H, two isomers), 7.03-6.95 (m, 1H, two isomers), 5.19 (d, *J* = 8.0 Hz, 0.1H, one isomer), 4.80 (d, *J* = 5.6 Hz, 0.9H, one isomer), 3.42-3.32 (m, 0.9H, one isomer), 3.25 (dd, *J* = 15.2, 7.6 Hz, 0.1H, one isomer), 2.99-2.88 (m, 1H, two isomers), 2.87-2.83 (m, 0.1H, one isomer), 2.77 (dd, *J* = 16.8, 6.0 Hz, 0.9H, one isomer), 1.82-1.72 (m, 1H, two isomers), 1.69-1.60 (m, 1H, two isomers), 1.52-1.43 (m, 1H, two isomers), 1.39 (s, 8.1H, one isomer), 1.33 (s, 9H, two isomer), 1.32 (s, 0.9H, one isomer), 1.03-0.96 (m, 6H, two isomers); ^{13}C NMR (100 MHz, $CDCl_3$) δ 177.5 & 175.8 (two isomers), 171.5 & 169.9 (two isomers), 148.1 (overlap, two isomers), 147.0 & 146.7 (two isomers), 140.4 & 140.1 (two isomers), 134.1 & 134.0 (two isomers), 131.0 (overlap, two isomers), 128.5 & 128.4 (two isomers), 128.0 & 128.0 (two isomers), 124.2 & 124.1 (two isomers), 123.8 & 123.6 (two isomers), 122.9 & 122.7 (two isomers), 80.9 & 77.8 (two isomers), 44.7 (overlap, two isomers), 42.1 & 40.8 (two isomers), 39.8 & 39.3 (two isomers), 34.8 (overlap, two isomers), 34.7 & 34.7 (two isomers), 31.5 (overlap, two isomers), 30.2 & 30.1 (two isomers), 27.3 & 26.7 (two isomers), 23.5 & 23.0 (two isomers), 22.3 & 21.9 (two isomers). FT-IR: ν (cm^{-1}) 2956, 2905, 2870, 1753, 1615, 1576, 1540, 1494, 1449, 1398. HRMS [ESI] calcd for $C_{29}H_{40}NO_2$ $[M+H]^+$ 434.3054, found 434.3051.

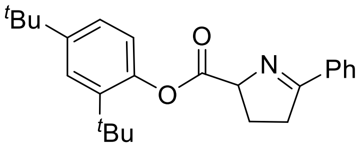

3ad: 64.1 mg, 85% yield, yellow solid, m.p. 88-89 °C. Purification by flash column chromatography on silica gel (eluent: EtOAc/Petroleum ether = 1/8). ^1H NMR (400 MHz, $CDCl_3$) δ 7.96-7.91 (m, 2H), 7.48-7.41 (m, 3H), 7.40 (d, *J* = 2.4, 1H), 7.23 (dd, *J* = 8.4, 2.4 Hz, 1H), 6.98 (d, *J* = 8.4 Hz, 1H), 5.24-5.16 (m, 1H), 3.26-3.05 (m, 2H), 2.57-2.39 (m, 2H), 1.36 (s, 9H), 1.32 (s, 9H); ^{13}C NMR (100 MHz, $CDCl_3$) δ 176.4, 171.5, 148.2, 146.9, 140.1, 133.8, 131.1, 128.5, 128.1, 124.1, 123.8, 122.9, 75.1, 35.5, 34.7, 34.7, 31.5, 30.2, 26.5. FT-IR: ν (cm^{-1}) 2997, 2964, 2870, 2159, 1976, 1718, 1653, 1636, 1609, 1507, 1445. HRMS [ESI] calcd for $C_{25}H_{32}NO_2$ $[M+H]^+$ 378.2428, found 378.2419.

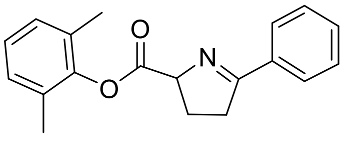

3ae: 51.0 mg, 87% yield, yellow solid, m.p. 64-65 °C. Purification by flash column chromatography on silica gel (eluent: EtOAc/Petroleum ether = 1/8). ^1H NMR (400 MHz, $CDCl_3$) δ 7.96-7.91 (m, 2H), 7.51-7.41 (m, 3H), 7.09-7.01 (m, 3H), 5.27-5.20 (m, 1H), 3.29-3.06 (m, 2H), 2.57-2.42 (m, 2H), 2.19 (s, 6H); ^{13}C NMR (100 MHz, $CDCl_3$) δ 176.4, 170.5, 148.1, 133.8, 131.1, 130.2, 128.6, 128.5, 128.1, 125.9, 74.7, 35.6, 26.5, 16.4. FT-IR: ν (cm^{-1}) 3026, 2921, 2852, 1981, 1747, 1618, 1574, 1494, 1471, 1450. HRMS [ESI] calcd for $C_{19}H_{19}NO_2Na$ $[M+Na]^+$ 316.1308, found 316.1294.

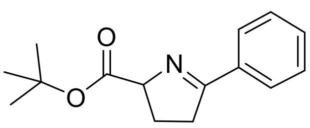

3af: 46.6 mg, 95% yield, yellow solid, m.p. 42-43 °C. Purification by flash column chromatography on silica gel (eluent: EtOAc/Petroleum ether = 1/8). ^{1}H NMR (400 MHz, $CDCl_3$) δ 7.90-7.85 (m, 2H), 7.44-7.37 (m, 3H), 4.84-4.78 (m, 1H), 3.17-3.06 (m, 1H), 3.02-2.91 (m, 1H), 2.37-2.26 (m, 1H), 2.21-2.10 (m, 1H), 1.49 (s, 9H); ^{13}C NMR (100 MHz, $CDCl_3$) δ 175.9, 172.3, 134.0, 130.8, 128.4, 128.0, 81.2, 75.3, 35.4, 28.1, 26.7. FT-IR: ν (cm^{-1}) 2980, 2928, 2159, 2031, 1727, 1653, 1636, 1604, 1507, 1447. HRMS [ESI] calcd for $C_{15}H_{19}NO_2Na$ $[M+Na]^+$ 268.1308, found 268.1300.

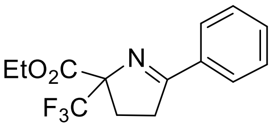

3ag: 52.5 mg, 92% yield, yellow oil. Purification by flash column chromatography on silica gel (eluent: EtOAc/Petroleum ether = 1/8). ^{1}H NMR (400 MHz, $CDCl_3$) δ 7.95-7.91 (m, 2H), 7.54-7.48 (m, 1H), 7.47-7.41 (m, 2H), 4.29 (q, J = 7.2 Hz, 2H), 3.21 (t, J = 8.0 Hz, 2H), 2.64-2.54 (m, 1H), 2.51-2.41 (m, 1H), 1.31 (t, J = 7.2 Hz, 3H); ^{13}C NMR (100 MHz, $CDCl_3$) δ 179.9, 167.9, 132.9, 131.8, 128.6, 128.5, 124.6 (q, $J_{C\text{-}F}$ = 280.8 Hz), 85.3 (q, $J_{C\text{-}F}$ = 26.9 Hz), 62.4, 36.0, 28.0 (q, $J_{C\text{-}F}$ = 1.1 Hz), 14.0; ^{19}F NMR (376 MHz, $CDCl_3$) δ -74.7 (s). FT-IR: ν (cm^{-1}) 3006, 2921, 2849, 2158, 1965, 1714, 1658, 1613, 1604, 1502, 1424. HRMS [EI] calcd for $C_{14}H_{14}F_3NO_2$ $[M]^+$ 285.0971, found 285.0976.

5. Gram-scale reaction

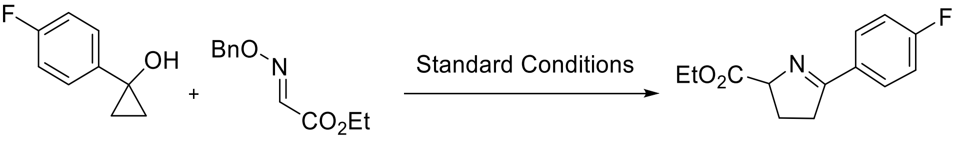

1b **2a** **3b**

Cyclopropanol **1b** (3.0 mmol, 1.5 equiv.), oxime ether **2a** (2.0 mmol, 1.0 equiv.), and $MnCl_2$ (0.4 mmol, 20 mol %) were loaded in a flame-dried reaction vial, which was subjected to evacuation/ flushing with nitrogen three times. HFIP (40 mL) was added to the mixture via syringe. Then acetylacetone (acac, 2.0 mmol, 1.0 equiv.) and AcOH (2.0 mmol, 1.0 equiv.) were added to the mixture, which was stirred at room temperature for 14 h. The mixture was quenched with H_2O. The aqueous layer was extracted with DCM. The organic layer was washed with brine, dried over Na_2SO_4, concentrated in vacuum, and purified by flash column chromatography on silica gel (eluent: EtOAc/Petroleum ether) to give the corresponding product **3b** (0.376 g, 78% yield).

6. Transformation of compound 3a

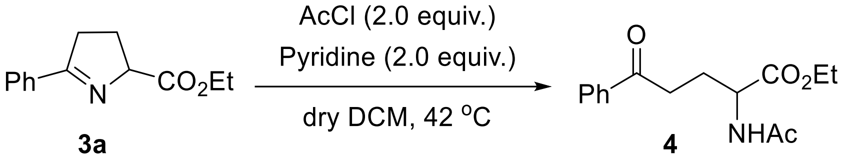

Under argon atmosphere, to a stirred solution of **3a** (33.0 mg, 0.15 mmol, 1.0 equiv.) in dry DCM (1.5 ml) was added fresh acetyl chloride (0.45 mmol, 35.3 mg, 32.0 μl, 3.0 equiv.) and pyridine (0.3 mmol, 23.7 mg, 24.2 μl, 2.0 equiv.) dropwise, The reaction mixture was stirred at 42

°C. Upon reaction completion, the mixture was concentrated in vacuo and purified by flash column chromatography on silica gel to afford the compound **4**.

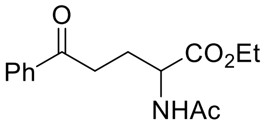

4: 26.2 mg, 63% yield, yellow solid, m.p. 100-101 °C. Purification by flash column chromatography on silica gel (eluent: EtOAc/Petroleum ether = 1/5). ^{1}H NMR (400 MHz, $CDCl_3$) δ 7.96-7.90 (m, 2H), 7.60-7.53 (m, 1H), 7.49-7.42 (m, 2H), 6.31 (d, *J* = 7.6 Hz, 1H), 4.68-4.60 (m, 1H), 4.25-4.14 (m, 2H), 3.19-2.99 (m, 2H), 2.37-2.26 (m, 1H), 2.20-2.08 (m, 1H), 1.99 (s, 3H), 1.26 (t, *J* = 7.2 Hz, 3H); ^{13}C NMR (100 MHz, $CDCl_3$) δ 199.2, 172.2, 170.1, 136.6, 133.3, 128.7, 128.0, 61.7, 52.0, 34.7, 26.7, 23.2, 14.1. FT-IR: ν (cm^{-1}) 3265, 3078, 2972, 2941, 2893, 1732, 1677, 1646, 1597, 1450. HRMS [ESI] calcd for $C_{15}H_{19}NO_4Na$ $[M+Na]^+$ 300.1206, found 300.1213.

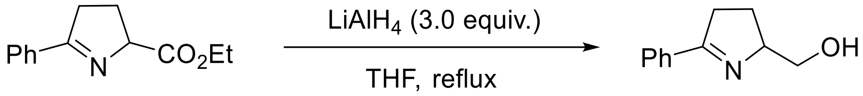

3a **5**

A screw-cap vial under N_2 was charged with **3a** (0.15 mmol, 1.0 equiv.) and dry THF (2.0 mL). At 0 °C, the solution of $LiAlH_4$ (0.45 mmol, 3.0 equiv.) in dry THF (2.0 mL) was added to the vial in small portions and the mixture was allowed to react at refluxing temperature. After stirring for 4 h, the reaction mixture was cooled to r.t. and quenched by saturated aq. NH_4Cl, the mixture was extracted with EtOAc for three times. Then the organic phase was combined and dried over anhydrous $MgSO_4$. The solvent was removed under vacuum and the residue was purified by flash column chromatography on silica gel to provide the product **5**.

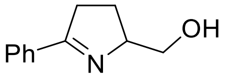

5: 23.6 mg, 90% yield, brown oil. Purification by flash column chromatography on silica gel (eluent: EtOAc/Petroleum ether = 1/1). ^{1}H NMR (400 MHz, $CDCl_3$) δ 7.81-7.75 (m, 2H), 7.47-7.35 (m, 3H), 4.40-4.31 (m, 1H), 4.02 (dd, *J* = 11.2, 3.6 Hz, 1H), 3.68-3.60 (m, 1H), 3.09-2.98 (m, 1H), 2.95-2.83 (m, 1H), 2.83 (br, 1H), 2.19-2.07 (m, 1H), 1.90-1.75 (m, 1H); ^{13}C NMR (100 MHz, $CDCl_3$) δ 174.8, 133.9, 130.8, 128.5, 127.9, 74.6, 66.0, 35.7, 24.5. FT-IR: ν (cm^{-1}) 3394, 3132, 3055, 2960, 2920, 2849, 1683, 1646, 1615, 1452, 1088. HRMS [ESI] calcd for $C_{11}H_{13}NONa$ $[M+Na]^+$ 198.0889, found 198.0879.

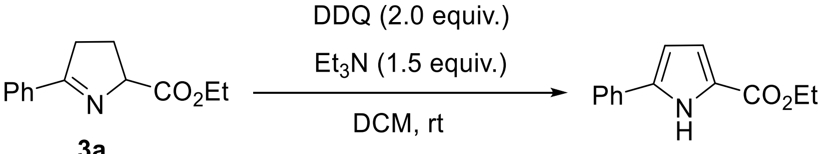

3a **6**

Triethylamine (0.18 mmol, 18.2 mg, 25.0 μl, 1.5 equiv.) was added to the solution of **3a** (29.3 mg, 0.12 mmol) and 2,3-dichloro-5,6-dicyano-1,4-benzoquinone (DDQ, 54.9 mg, 0.24 mmol, 2.0 equiv.) in CH_2Cl_2 (2 mL) under N_2, the reaction mixture was stirred at r.t. After stirring for 3 h, the solvent was removed under vacuum and the residue was purified by flash column chromatography on silica gel to provide the product **6**.

6: 20.5 mg, 53% yield, white solid, m.p. 120-121 °C. Purification by flash column chromatography on silica gel (eluent: EtOAc/Petroleum ether = 1/6). ^{1}H NMR (400 MHz, $CDCl_3$) δ 9.54 (s, 1H), 7.64-7.55 (m, 2H), 7.45-7.37 (m, 2H), 7.34-7.27 (m, 1H), 6.97 (s, 1H), 6.54 (s, 1H), 4.35 (q, J = 7.2 Hz, 2H), 1.38 (t, J = 7.2 Hz, 3H); ^{13}C NMR (100 MHz, $CDCl_3$) δ 161.4, 136.8, 131.4, 129.0, 127.7, 124.8, 123.4, 116.7, 107.9, 60.5, 14.5. FT-IR: ν (cm^{-1}) 3316, 3278, 2993, 2921, 2850, 1682, 1603, 1583, 1510, 1461. HRMS [ESI] calcd for $C_{13}H_{13}NO_2Na$ $[M+Na]^+$ 238.0838, found 238.0830.

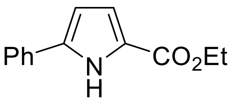

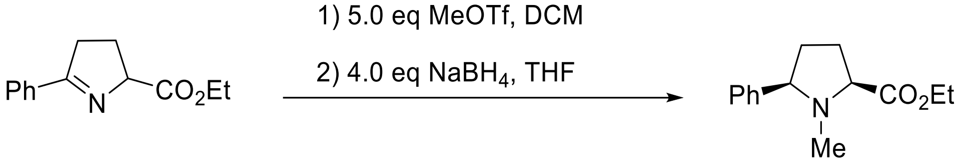

3a **7**

3a (0.2 mmol, 1.0 equiv.), activated 4 Å powdered molecular sieves (300 mg), and anhydrous CH_2Cl_2 (3 mL) was stirred at r.t. for 15 min, and then MeOTf (1.0 mmol, 5.0 equiv.) was added in twice. The suspension was stirred at r.t. for 4 h and then concentrated to dryness without filtering off the molecular sieves. To a cooled (0 °C), stirred suspension of the crude salt in anhydrous THF (3 mL) was added $NaBH_4$ (0.8 mmol, 4.0 equiv.). The mixture was stirred at rt. until the starting material had been consumed as determined by TLC. The crude mixture was quenched by NH_4Cl. The resulting aqueous phase was extracted with Et_2O. The combined organic extracts were washed with brine, dried over $MgSO_4$, filtered and concentrated in vacuo. The residue was eluted from a short column of silica gel (EtOAc/petroleum ether =1:3) to afford **7**.

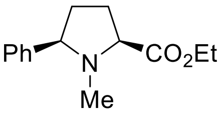

7-*cis*: 18.6 mg, 40% yield, yellow oil. Purification by flash column chromatography on silica gel (eluent: EtOAc/Petroleum ether = 1/3). ^{1}H NMR (400 MHz, $CDCl_3$) δ 7.45-7.39 (m, 2H), 7.35-7.29 (m, 2H), 7.26-7.21 (m, 1H), 4.23 (q, J = 7.2 Hz, 2H), 3.40-3.33 (m, 1H), 3.21-3.14 (m, 1H), 2.24 (s, 3H), 2.18-2.02 (m, 3H), 1.90-1.78 (m, 1H), 1.31 (t, J = 7.2 Hz, 3H); ^{13}C NMR (100 MHz, $CDCl_3$) δ 174.0, 142.8, 128.4, 127.5, 127.2, 71.9, 68.1, 60.6, 39.8, 34.5, 27.8, 14.3. FT-IR: ν (cm^{-1}) 2973, 2882, 1734, 1653, 1455, 1380, 1322, 1274. HRMS [ESI] calcd for $C_{14}H_{19}NO_2Na$ $[M+Na]^+$ 256.1308, found 256.1300.

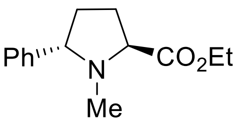

7-*trans*: 2.8 mg, 6% yield, yellow oil. ^{1}H NMR (400 MHz, $CDCl_3$) δ 7.59-7.54 (m, 2H), 7.42-7.36 (m, 3H), 4.49 (dd, J = 12.0, 7.6 Hz ,1H), 4.32-4.19 (m, 2H), 4.14 (t, J = 8.4 Hz, 1H), 2.65-2.52 (m, 1H), 2.45-2.35 (m, 1H), 2.32 (s, 3H), 2.31-2.19 (m, 2H), 1.33 (t, J = 7.2 Hz, 3H); ^{13}C NMR (100 MHz, $CDCl_3$) δ 169.3, 132.4, 131.2, 129.5, 128.1, 76.1, 72.4, 61.6, 39.6, 27.3, 25.6, 14.1. FT-IR: ν (cm^{-1}) 2973, 2882, 1734, 1653, 1455, 1380, 1322, 1274. HRMS [ESI] calcd for $C_{14}H_{19}NO_2Na$ $[M+Na]^+$ 256.1308, found 256.1300.

7. Mechanistic studies

7.1 Verification of Radical mechanism

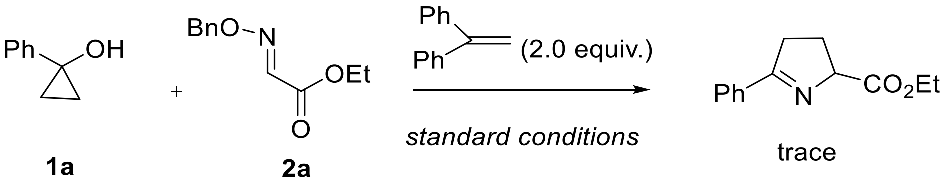

When ethene-1,1-diyldibenzene (2.0 equiv.) was introduced into the model reaction, only trace products were detected according to TLC analysis.

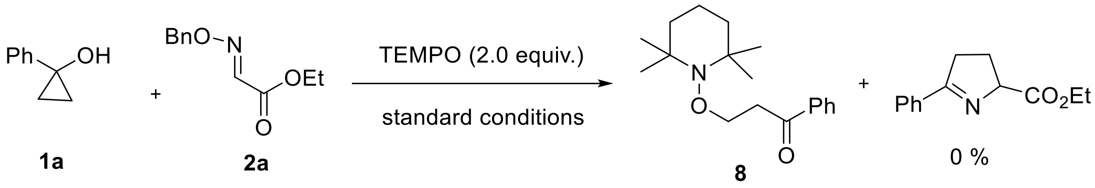

Following the standard procedures, additional 2.0 equiv. TEMPO was added. **8** was detected by HRMS. HRMS [ESI] calcd for $C_{18}H_{27}NO_2$ $[M]^+$ 289.2036, found 289.2038.

7.2 Reaction ratio plot of 20 mol% Mn(acac)$_3$ and Mn(acac)$_2$

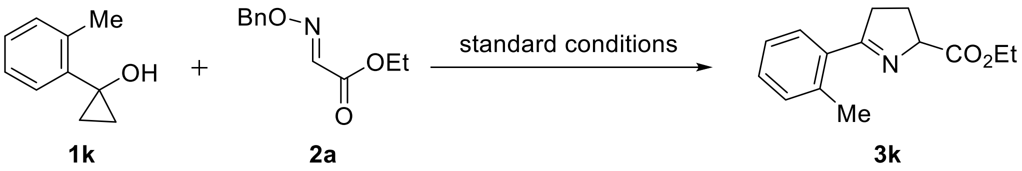

Table 1. The yields of reaction with **1k** and 20 mol% Mn(acac)$_3$ at different time interval

Time (min)	5	10	15	20
Yield/%	7	7	7	19

Table 2. The yields of reaction with **1k** and 20 mol% Mn(acac)$_2$ at different time interval

Time (min)	5	10	15	20
Yield/%	37	41	42	45

Figure 1. Plot of the reaction yields of Mn(acac)$_2$ and Mn(acac)$_3$ in different time

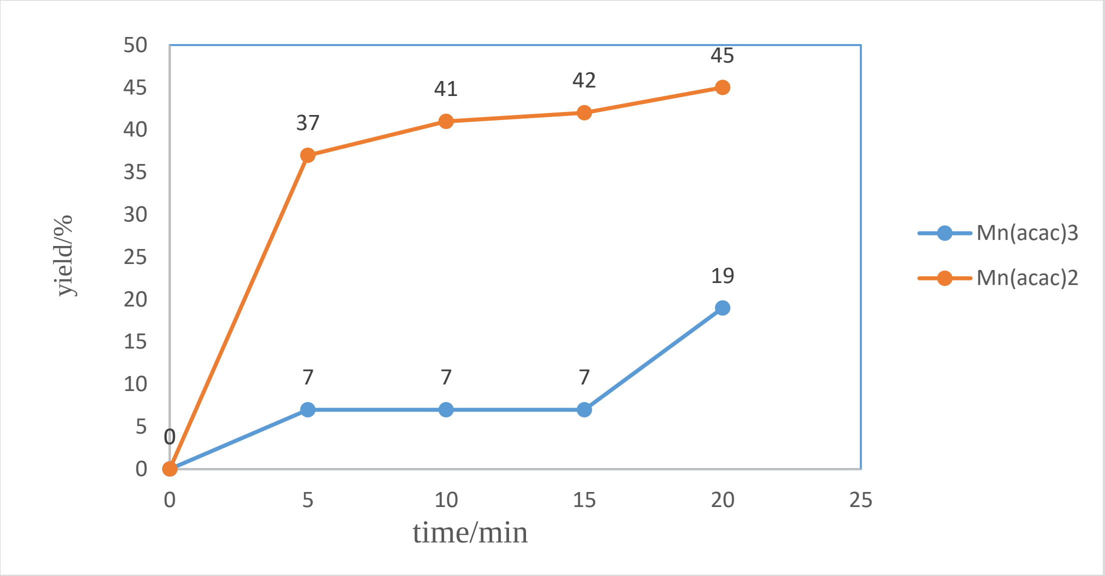

7.3 ^{1}H NMR experiments to verify the formation of Mn-O species from $Mn(acac)_2$

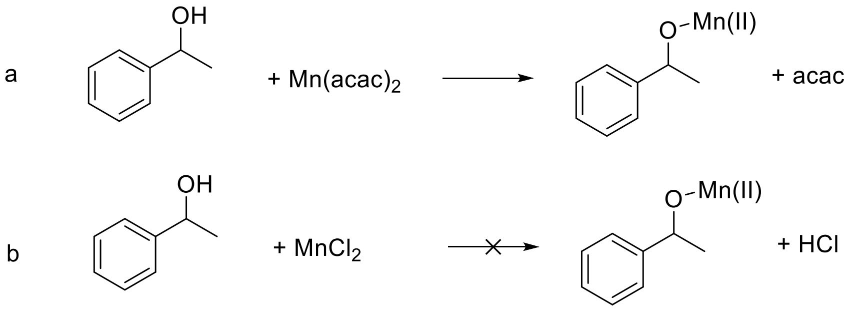

^{1}H NMR of 1-phenylethan-1-ol

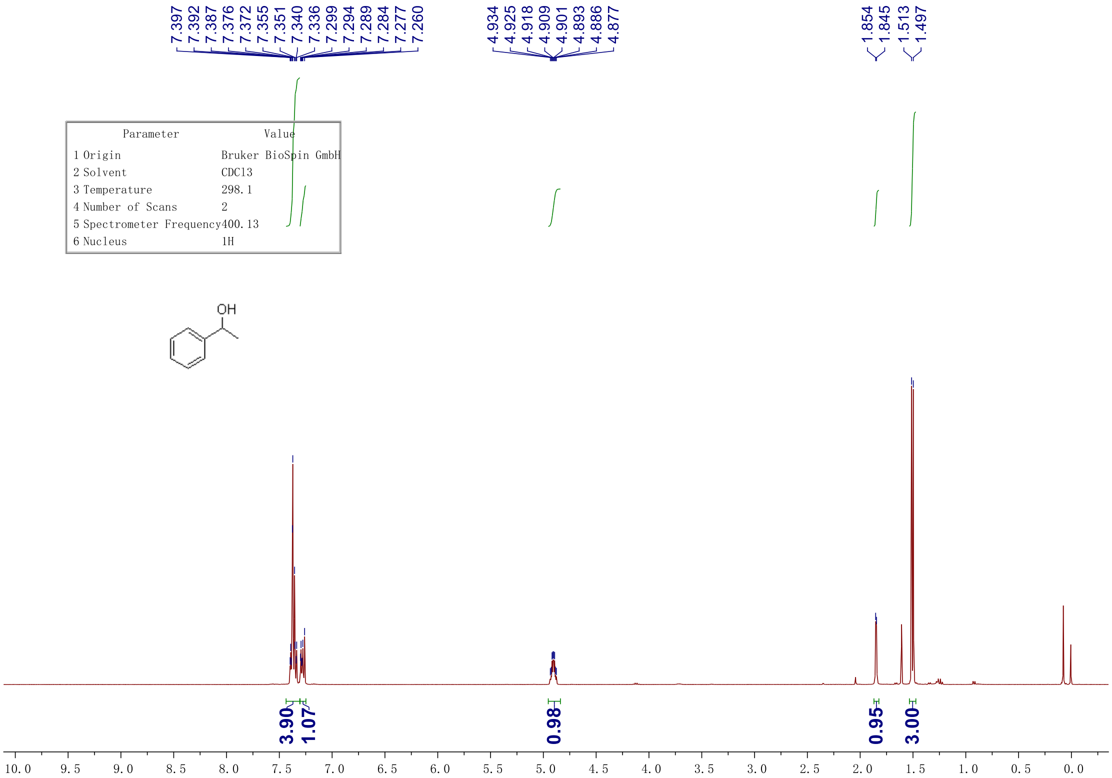

^{1}H NMR of 1-phenylethan-1-ol and $MnCl_2$ (*nothing changes in the spectrum*)

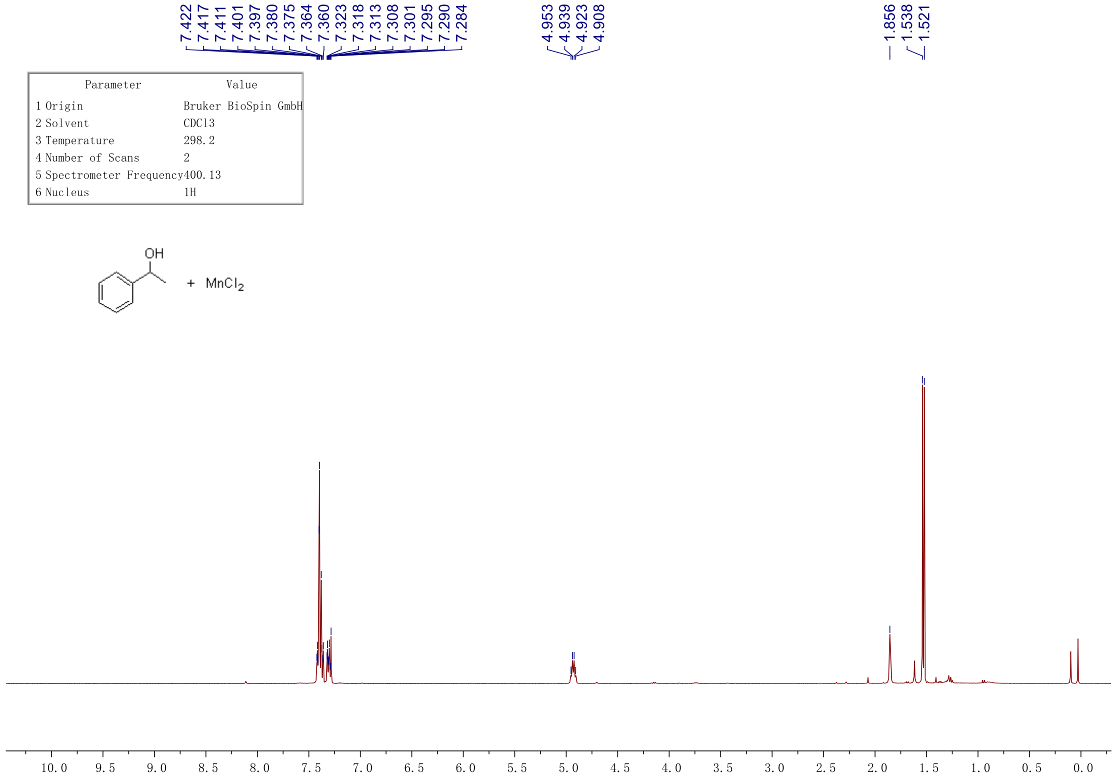

^{1}H NMR of 1-phenylethan-1-ol and $Mn(acac)_2$

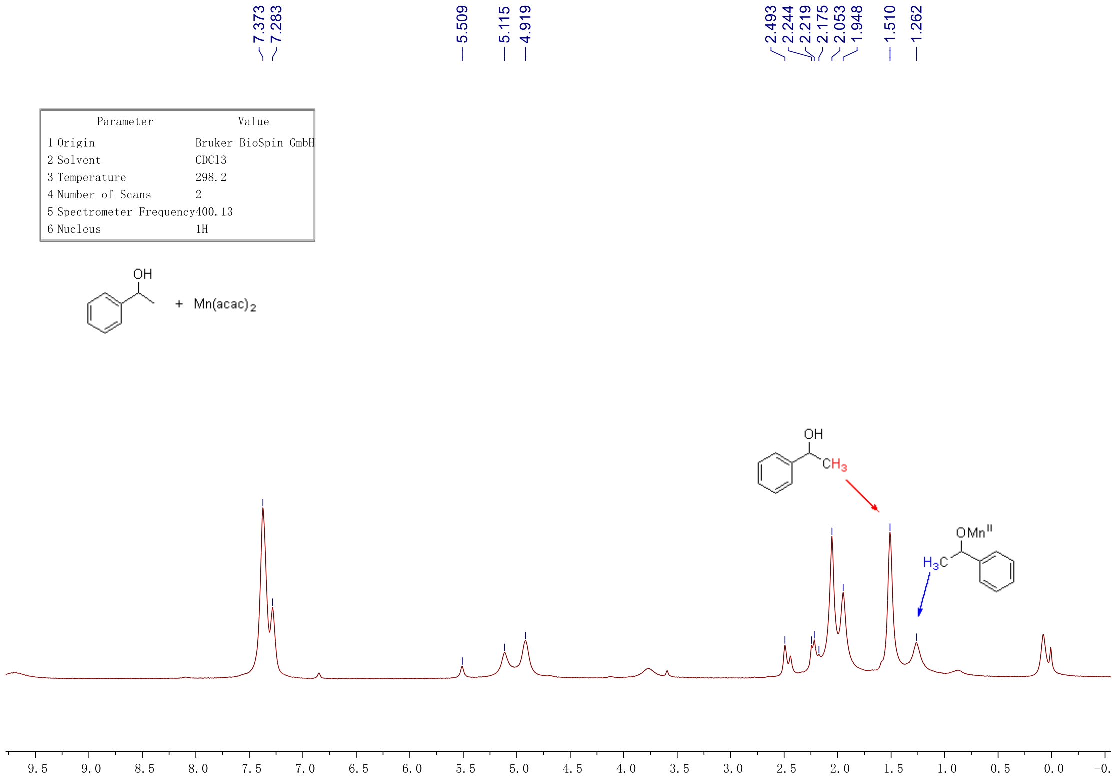

^{1}H NMR of acac

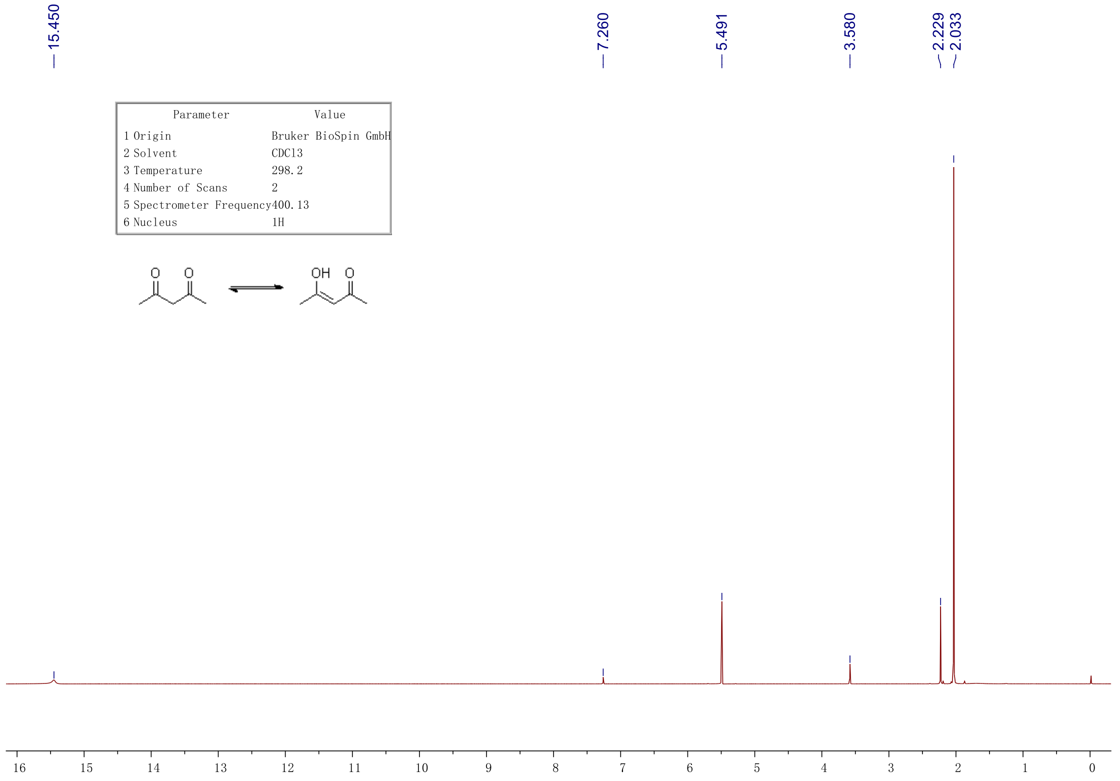

8. References

1. (a) M. Ji, Z. Wu, C. Zhu, *Chem. Commun.*, **2019**, 55, 2368-2371. (b) W. He, Y. Shu, J. Dai, W. Zhang, Y.-S. Feng, H. Xu, *Org. Biomol. Chem.*, **2015**, 13, 7159-7163. (c) D. Chen, Y. Fu, X. Cao, J. Luo, F. Wang, S. Huang, *Org. Lett.* **2019**, 21, 5600-5605. (d) R. Rivera, Y. Jang, C. Poteat, V. N. G. Lindsay, *Org. Lett.* **2020**, 22, 6510-6515. (e) M. Rubina, M. Rubin, *Chem. Heterocycl Comp.* **2012**, 48, 807-821. (f) G. Z. Elek, V. Borovkov, M. Lopp, D. G. Kananovich, *Org. Lett.* **2017**, 19, 3544-3547.
2. G. Wu, J. Wang, C. Liu, M. Sun, L. Zhang, Y. Ma, R. Cheng, J. Ye, *Org. Chem. Front*, **2019**, 6, 2245.

9. ^{1}H, ^{13}C, and ^{19}F NMR spectra

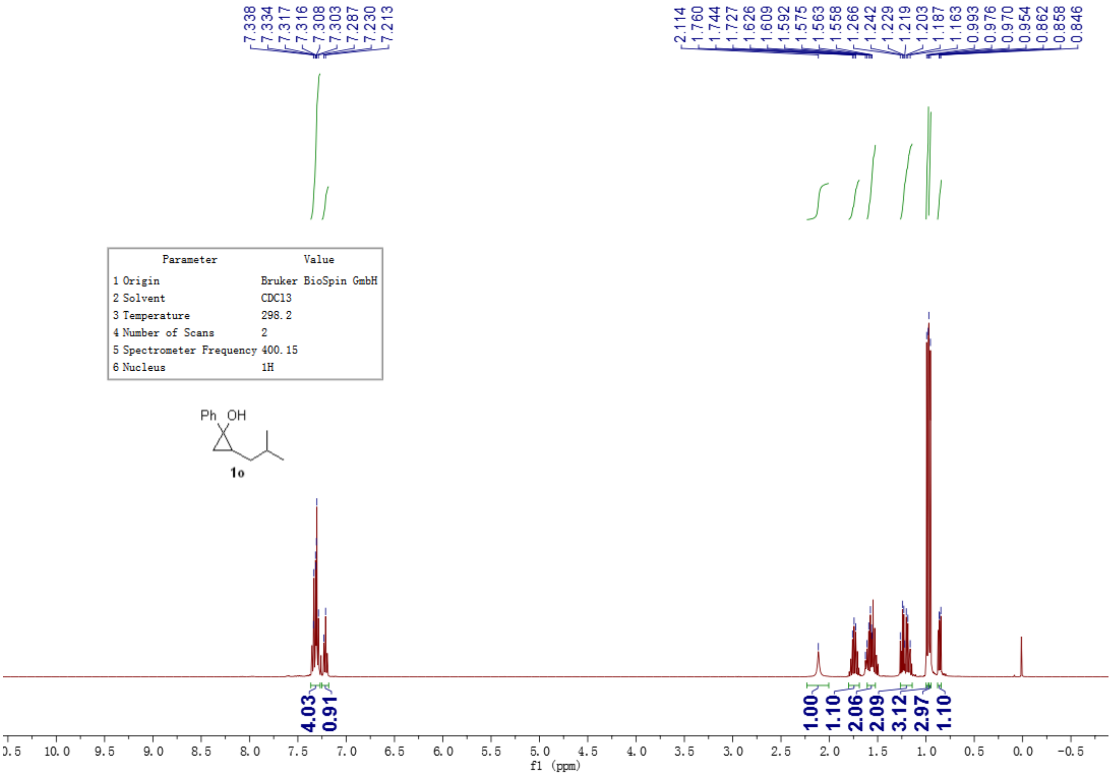

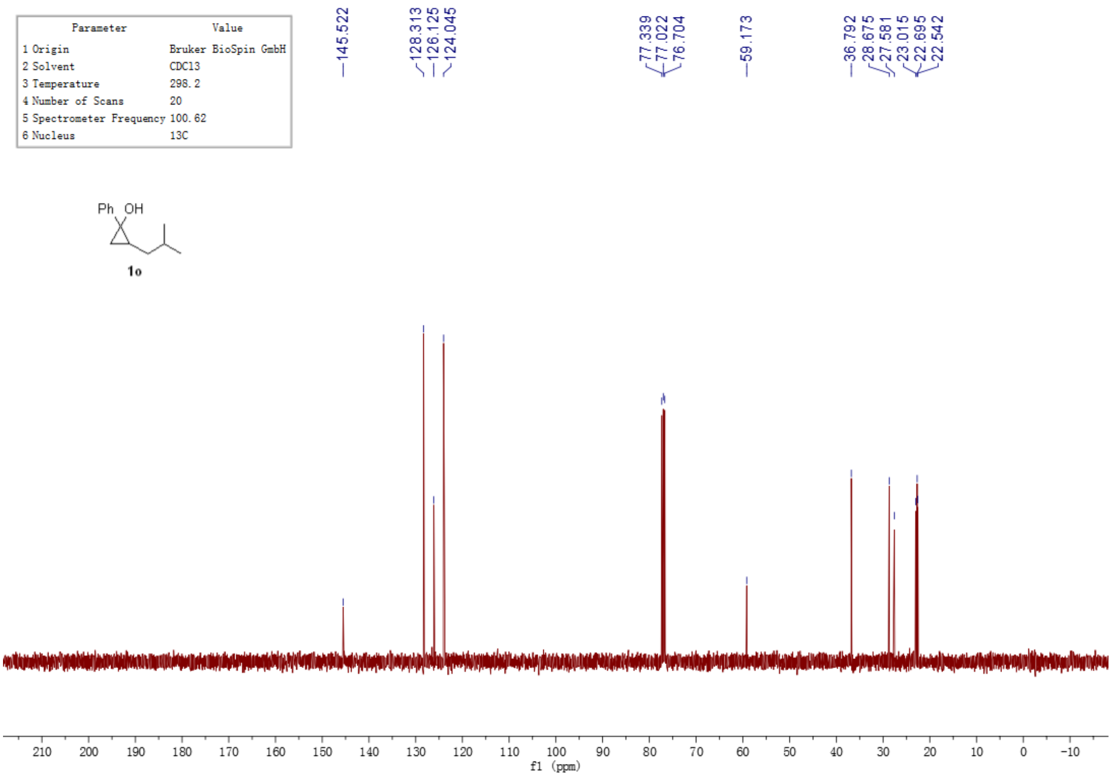

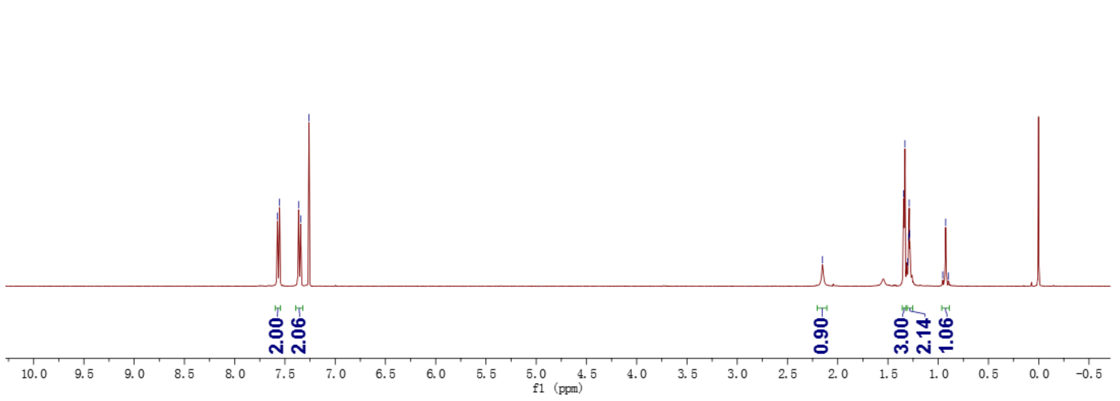

Parameter	Value
1 Origin	Bruker BioSpin GmbH
2 Solvent	CDCl3
3 Temperature	298.1
4 Number of Scans	2
5 Spectrometer Frequency	400.15
6 Nucleus	1H

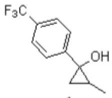

1r

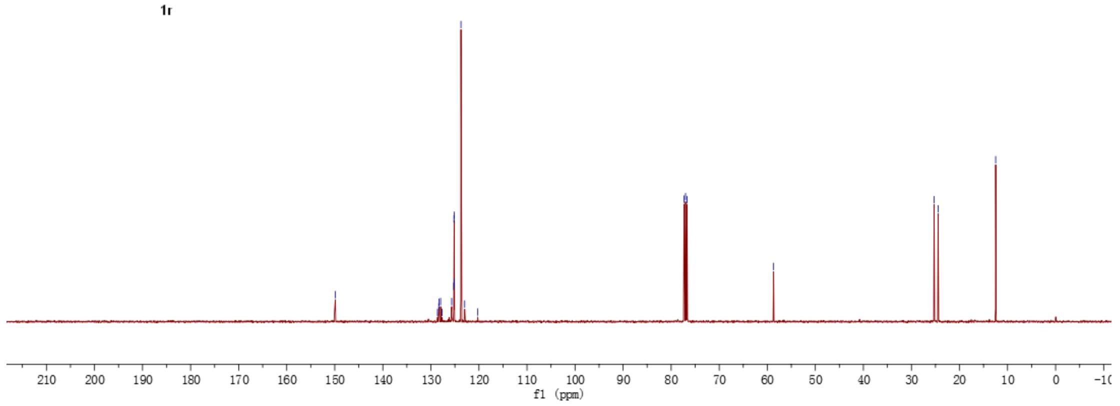

Parameter	Value
1 Origin	Bruker BioSpin GmbH
2 Solvent	CDCl3
3 Temperature	298.1
4 Number of Scans	1024
5 Spectrometer Frequency	100.62
6 Nucleus	13C

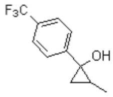

1r

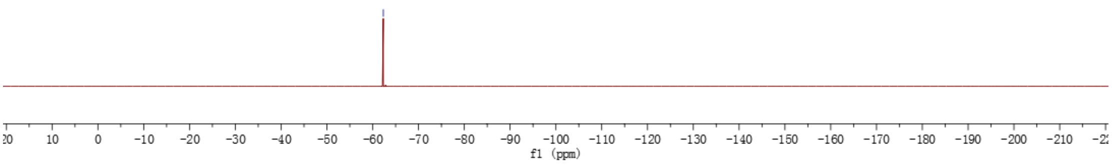

Parameter	Value
1 Origin	Bruker BioSpin GmbH
2 Solvent	CDCl3
3 Temperature	298.2
4 Number of Scans	2
5 Spectrometer Frequency	376.52
6 Nucleus	19F

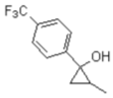

1r

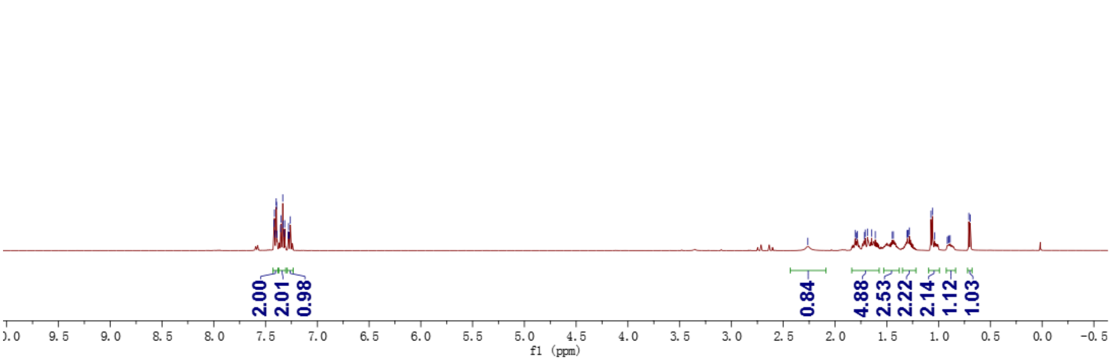

Parameter	Value
1 Origin	Bruker BioSpin GmbH
2 Solvent	CDCl3
3 Temperature	296.1
4 Number of Scans	2
5 Spectrometer Frequency	400.13
6 Nucleus	1H

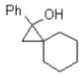

1t

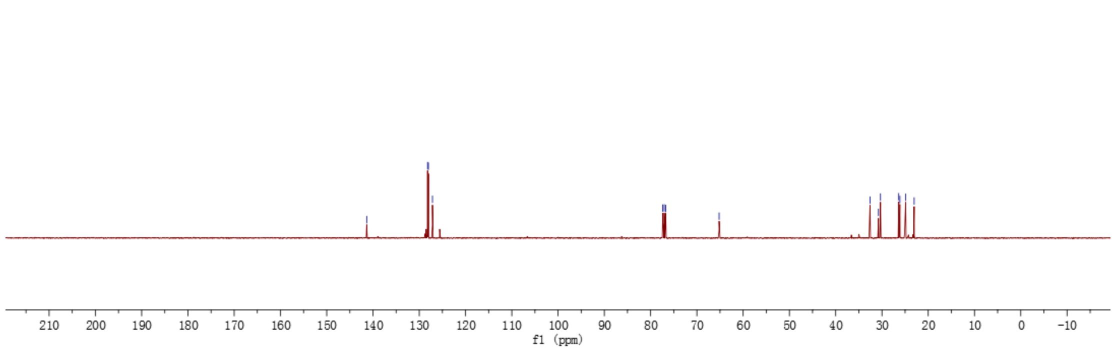

Parameter	Value
1 Origin	Bruker BioSpin GmbH
2 Solvent	CDCl3
3 Temperature	296.7
4 Number of Scans	80
5 Spectrometer Frequency	100.62
6 Nucleus	13C

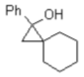

1t

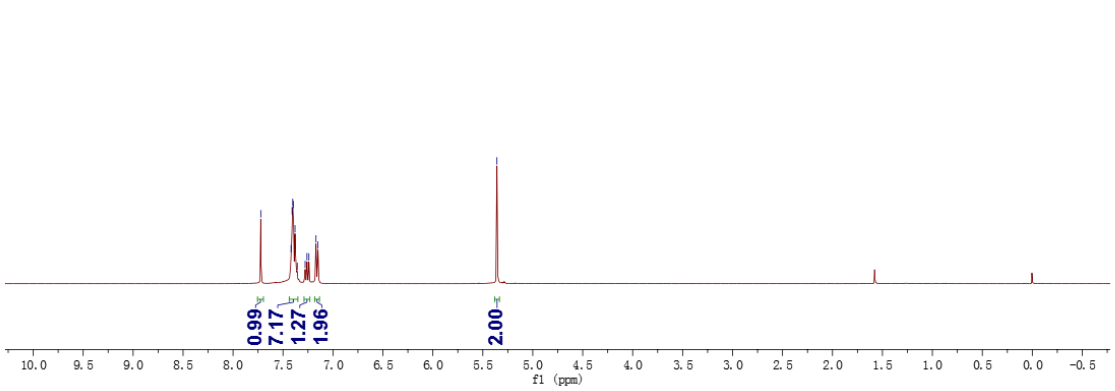

Parameter	Value
1 Origin	Bruker BioSpin GmbH
2 Solvent	CDCl3
3 Temperature	294.5
4 Number of Scans	4
5 Spectrometer Frequency	400.13
6 Nucleus	1H

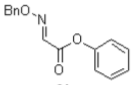

2b

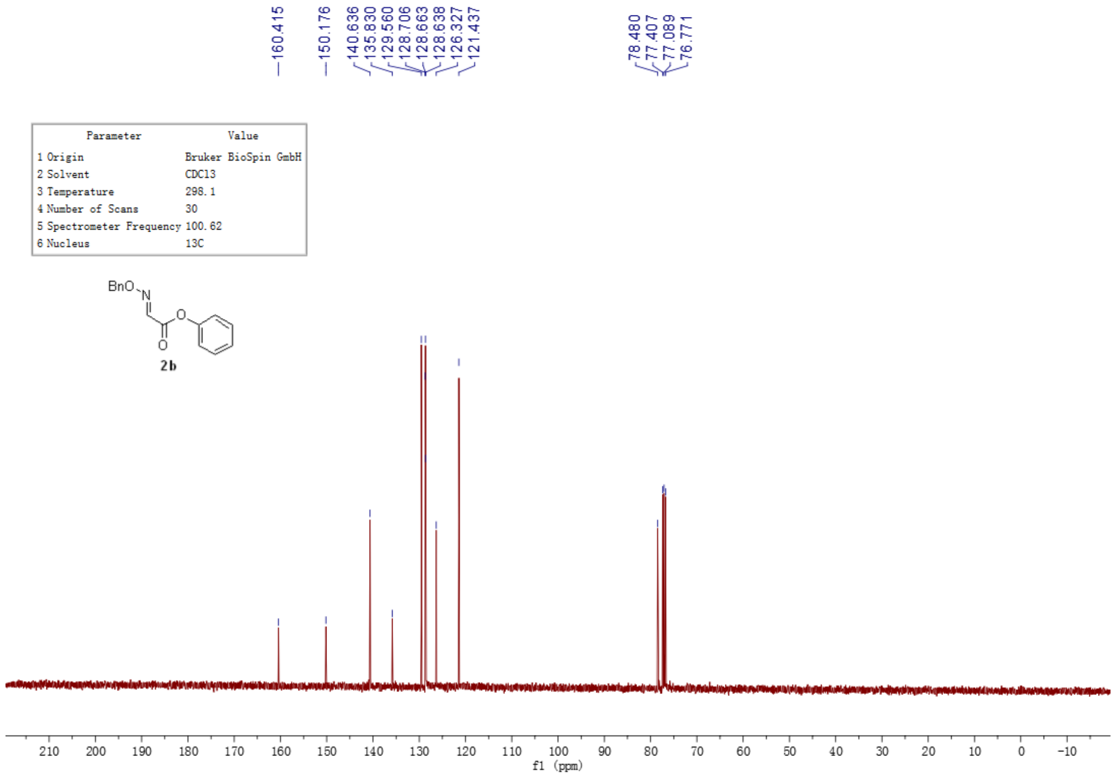
160.415
150.176
140.636
135.830
129.560
128.706
128.663
128.638
126.327
121.437
78.480
77.407
77.089
76.771
Parameter Value
1 Origin Bruker BioSpin GmbH
2 Solvent CDCl3
3 Temperature 298.1
4 Number of Scans 30
5 Spectrometer Frequency 100.62
6 Nucleus 13C
BnO
N
O
O
2b
f1 (ppm)

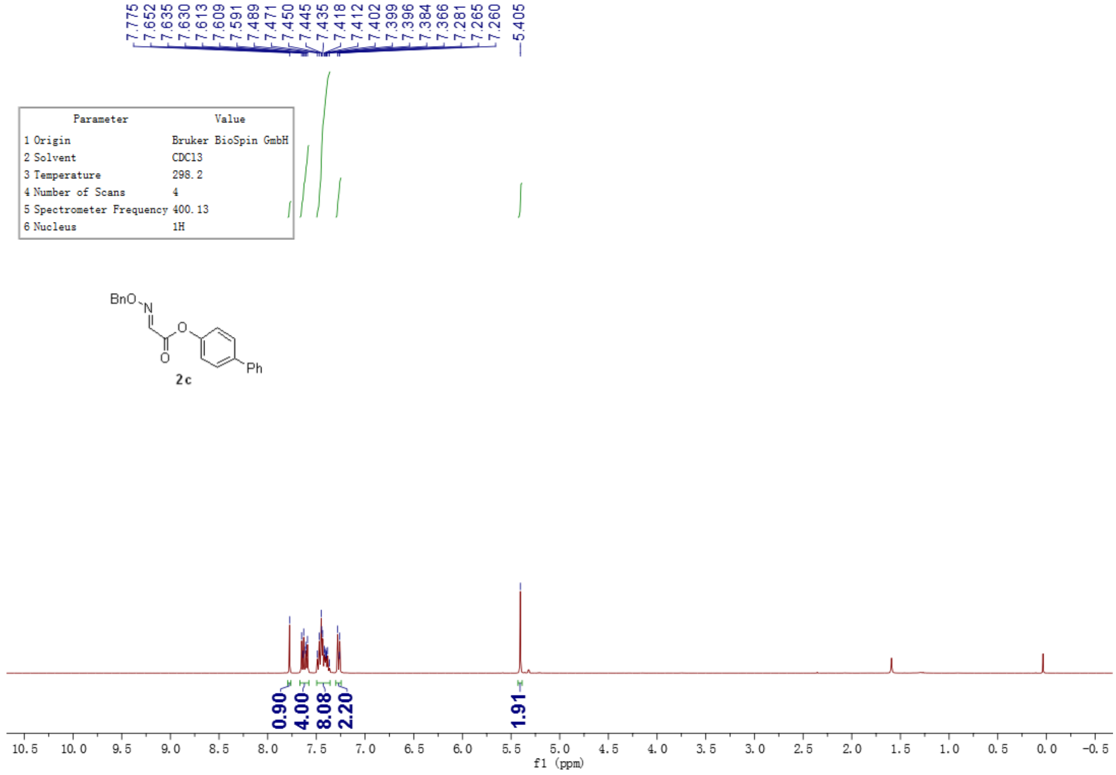
7.775
7.652
7.635
7.630
7.613
7.609
7.591
7.489
7.471
7.450
7.445
7.435
7.418
7.412
7.402
7.399
7.396
7.384
7.366
7.281
7.265
7.260
5.405
Parameter Value
1 Origin Bruker BioSpin GmbH
2 Solvent CDCl3
3 Temperature 298.2
4 Number of Scans 4
5 Spectrometer Frequency 400.13
6 Nucleus 1H
BnO
N
O
O
Ph
2c
0.90
4.00
8.08
2.20
1.91
f1 (ppm)

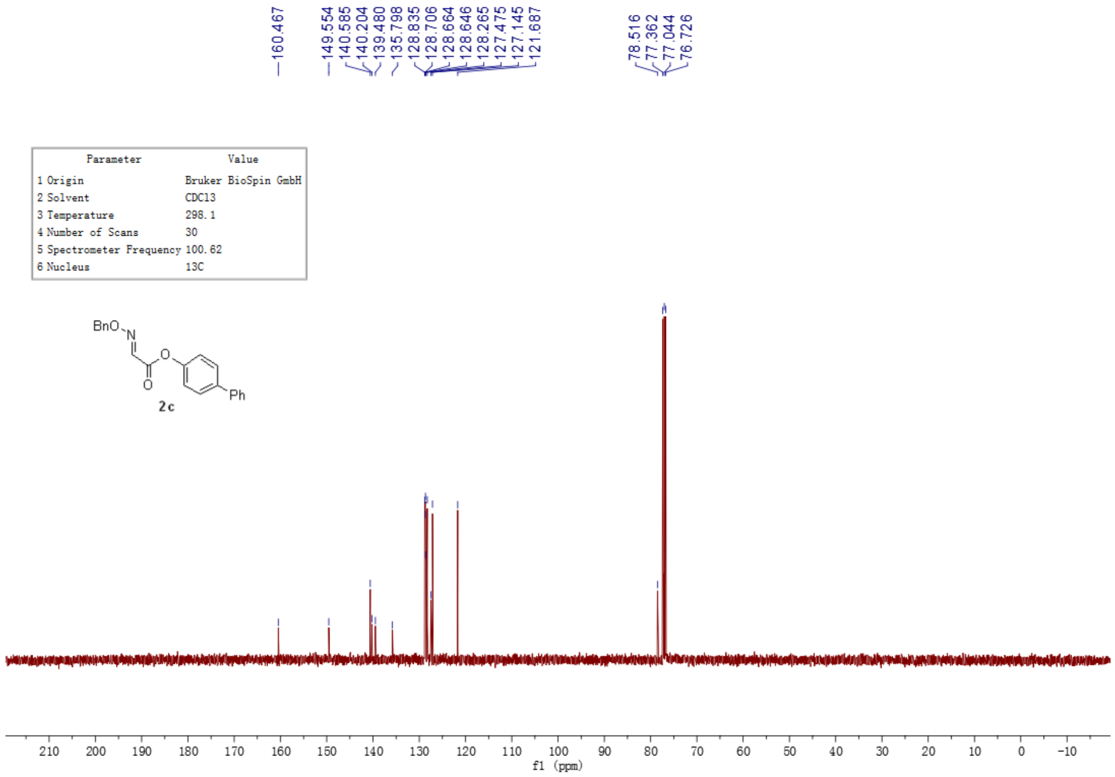
160.467
149.554
140.585
140.204
139.480
135.798
128.835
128.706
128.664
128.646
128.265
127.475
127.145
121.687
78.516
77.362
77.044
76.726
Parameter Value
1 Origin Bruker BioSpin GmbH
2 Solvent CDCl3
3 Temperature 298.1
4 Number of Scans 30
5 Spectrometer Frequency 100.62
6 Nucleus 13C
BnO
N
O
O
Ph
2c
f1 (ppm)

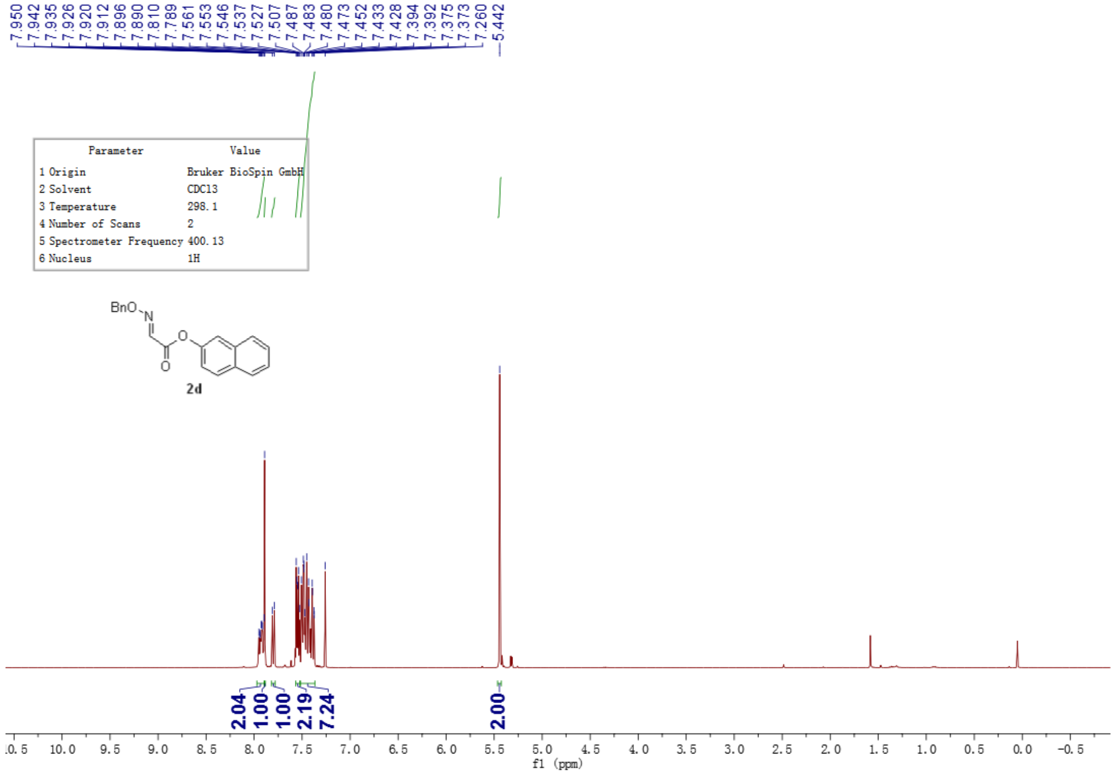
7.950
7.942
7.935
7.926
7.920
7.912
7.896
7.890
7.810
7.789
7.561
7.553
7.546
7.537
7.527
7.507
7.487
7.483
7.480
7.473
7.452
7.433
7.428
7.394
7.392
7.375
7.373
7.260
5.442
Parameter Value
1 Origin Bruker BioSpin GmbH
2 Solvent CDCl3
3 Temperature 298.1
4 Number of Scans 2
5 Spectrometer Frequency 400.13
6 Nucleus 1H
BnO
N
O
O
2d
2.04
1.00
1.00
2.19
7.24
2.00
f1 (ppm)

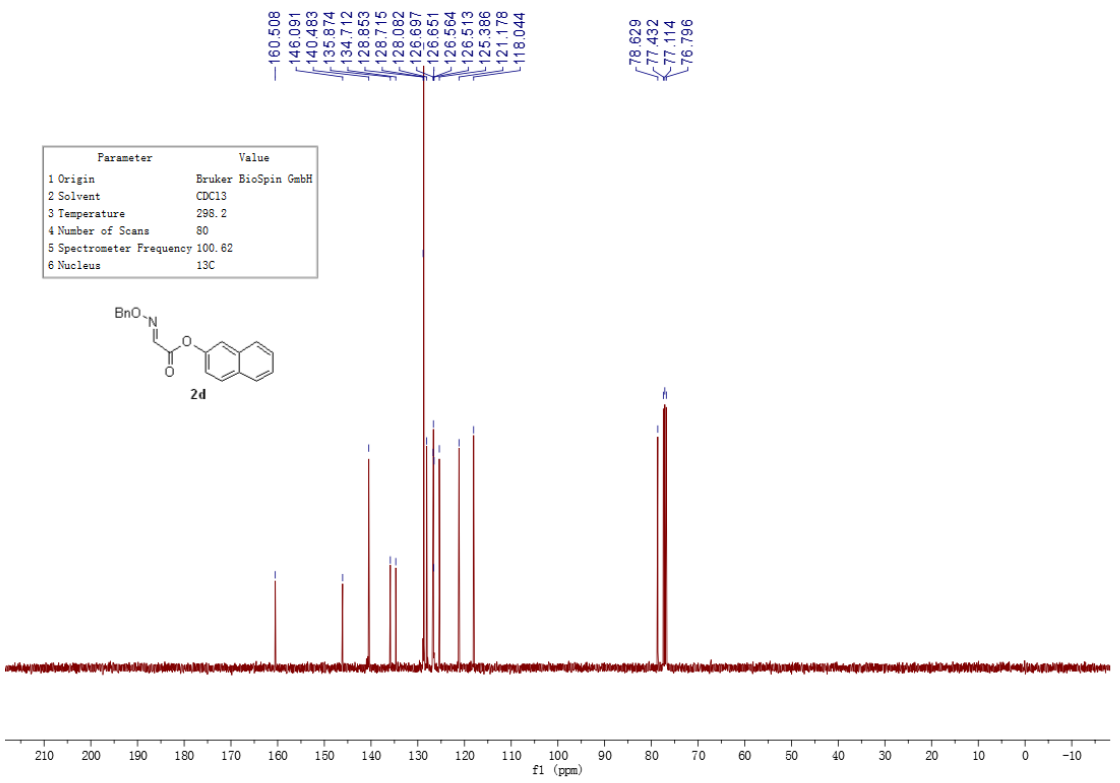
160.508
146.091
140.483
135.874
134.712
128.853
128.715
128.082
126.697
126.651
126.564
126.513
125.386
121.178
118.044
78.629
77.432
77.114
76.796
Parameter Value
1 Origin Bruker BioSpin GmbH
2 Solvent CDCl3
3 Temperature 298.2
4 Number of Scans 80
5 Spectrometer Frequency 100.62
6 Nucleus 13C
BnO
N
O
O
2d
f1 (ppm)

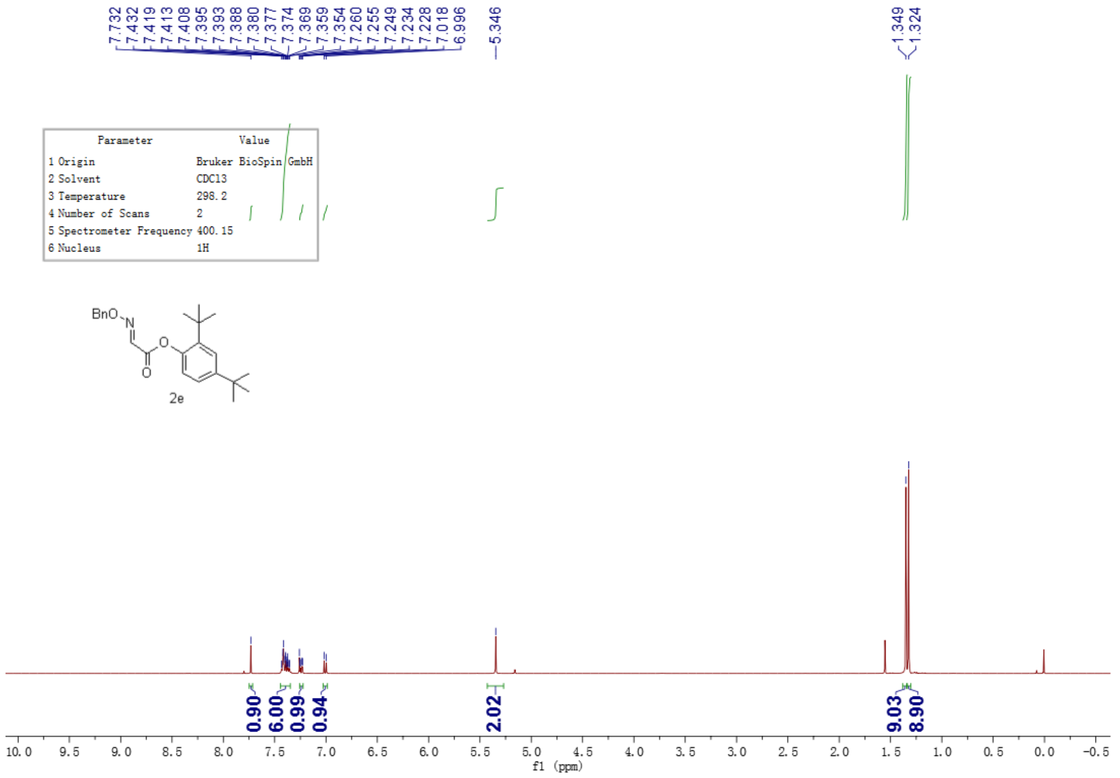
7.732
7.432
7.419
7.413
7.408
7.395
7.393
7.388
7.380
7.377
7.374
7.369
7.359
7.354
7.260
7.255
7.249
7.234
7.228
7.018
6.996
5.346
1.349
1.324
Parameter Value
1 Origin Bruker BioSpin GmbH
2 Solvent CDCl3
3 Temperature 298.2
4 Number of Scans 2
5 Spectrometer Frequency 400.15
6 Nucleus 1H
BnO
N
O
O
2e
0.90
6.00
0.99
0.94
2.02
9.03
8.90
f1 (ppm)

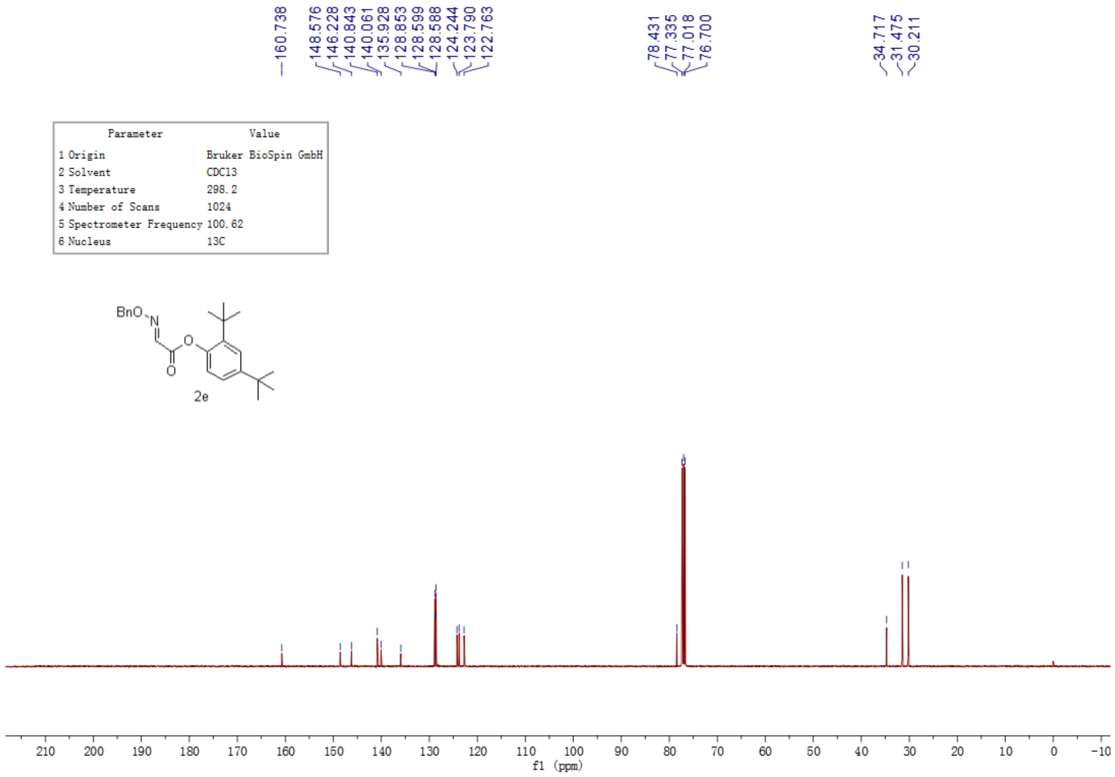
160.738
148.576
146.228
140.843
140.061
135.928
128.853
128.599
128.588
124.244
123.790
122.763
78.431
77.335
77.018
76.700
34.717
31.475
30.211
Parameter Value
1 Origin Bruker BioSpin GmbH
2 Solvent CDCl3
3 Temperature 298.2
4 Number of Scans 1024
5 Spectrometer Frequency 100.62
6 Nucleus 13C
BnO
N
O
O
2e
f1 (ppm)

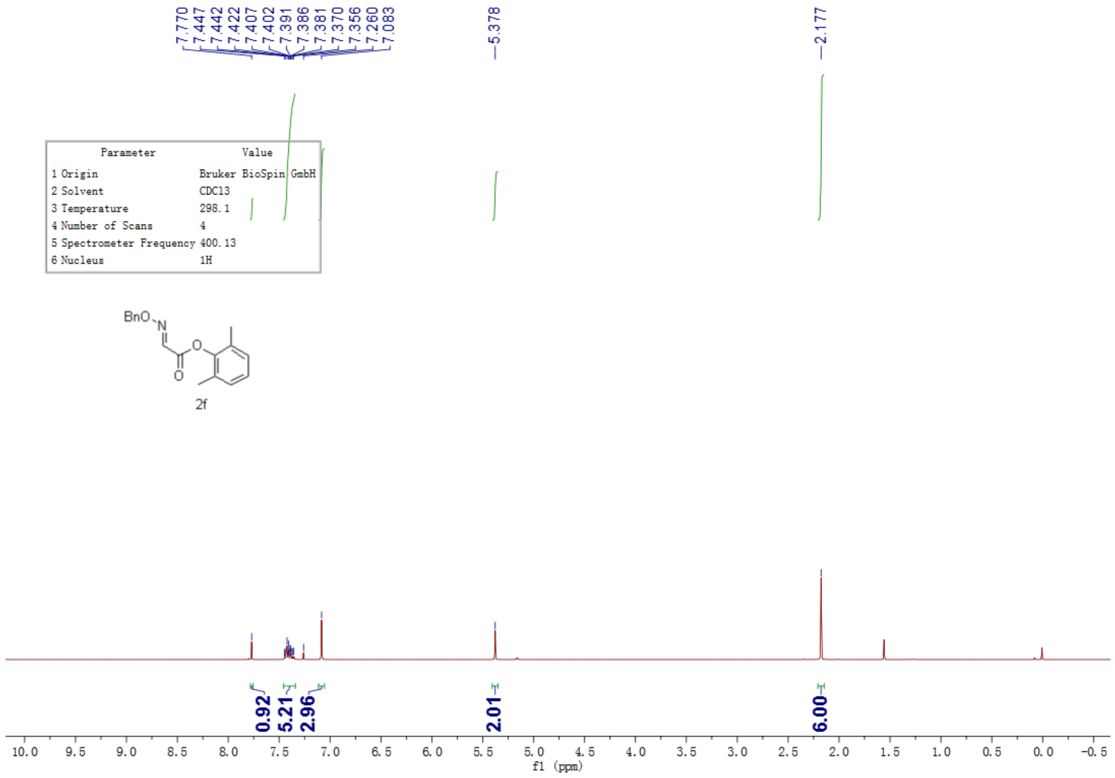
7.770
7.447
7.442
7.422
7.407
7.402
7.391
7.386
7.381
7.370
7.356
7.260
7.083
5.378
2.177
Parameter Value
1 Origin Bruker BioSpin GmbH
2 Solvent CDCl3
3 Temperature 298.1
4 Number of Scans 4
5 Spectrometer Frequency 400.13
6 Nucleus 1H
BnO
N
O
O
2f
0.92
5.21
2.96
2.01
6.00
f1 (ppm)

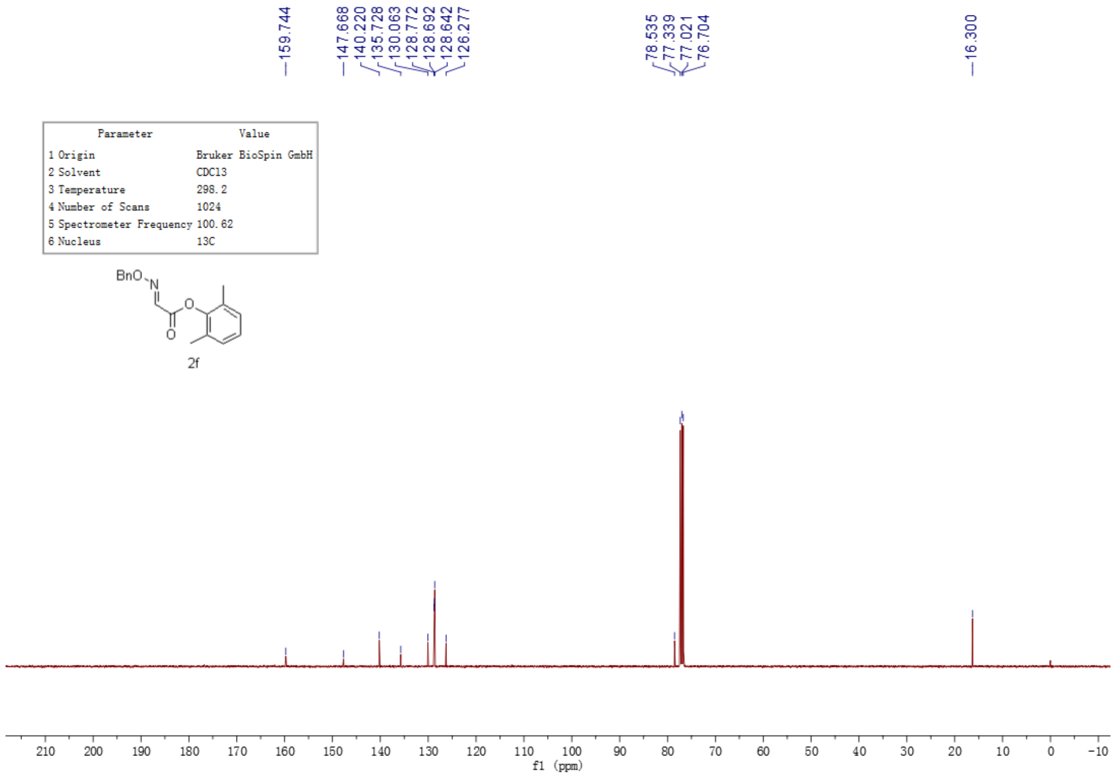
159.744
147.668
140.220
135.728
130.063
128.772
128.692
128.642
126.277
78.535
77.339
77.021
76.704
16.300
Parameter Value
1 Origin Bruker BioSpin GmbH
2 Solvent CDCl3
3 Temperature 298.2
4 Number of Scans 1024
5 Spectrometer Frequency 100.62
6 Nucleus 13C
BnO
N
O
O
2f
210 200 190 180 170 160 150 140 130 120 110 100 90 80 70 60 50 40 30 20 10 0 -10
f1 (ppm)

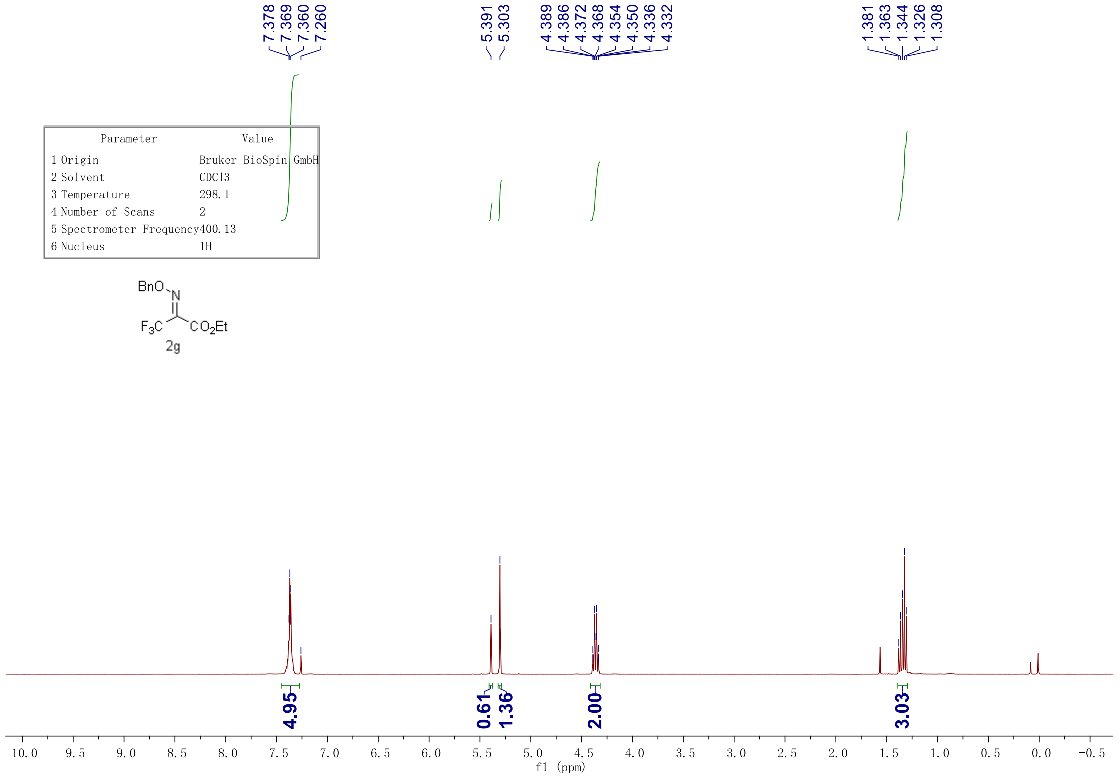
7.378
7.369
7.360
7.260
5.391
5.303
4.389
4.386
4.372
4.368
4.354
4.350
4.336
4.332
1.381
1.363
1.344
1.326
1.308
Parameter Value
1 Origin Bruker BioSpin GmbH
2 Solvent CDCl3
3 Temperature 298.1
4 Number of Scans 2
5 Spectrometer Frequency 400.13
6 Nucleus 1H
BnO
N
F3C
CO2Et
2g
4.95
0.61
1.36
2.00
3.03
10.0 9.5 9.0 8.5 8.0 7.5 7.0 6.5 6.0 5.5 5.0 4.5 4.0 3.5 3.0 2.5 2.0 1.5 1.0 0.5 0.0 -0.5
f1 (ppm)

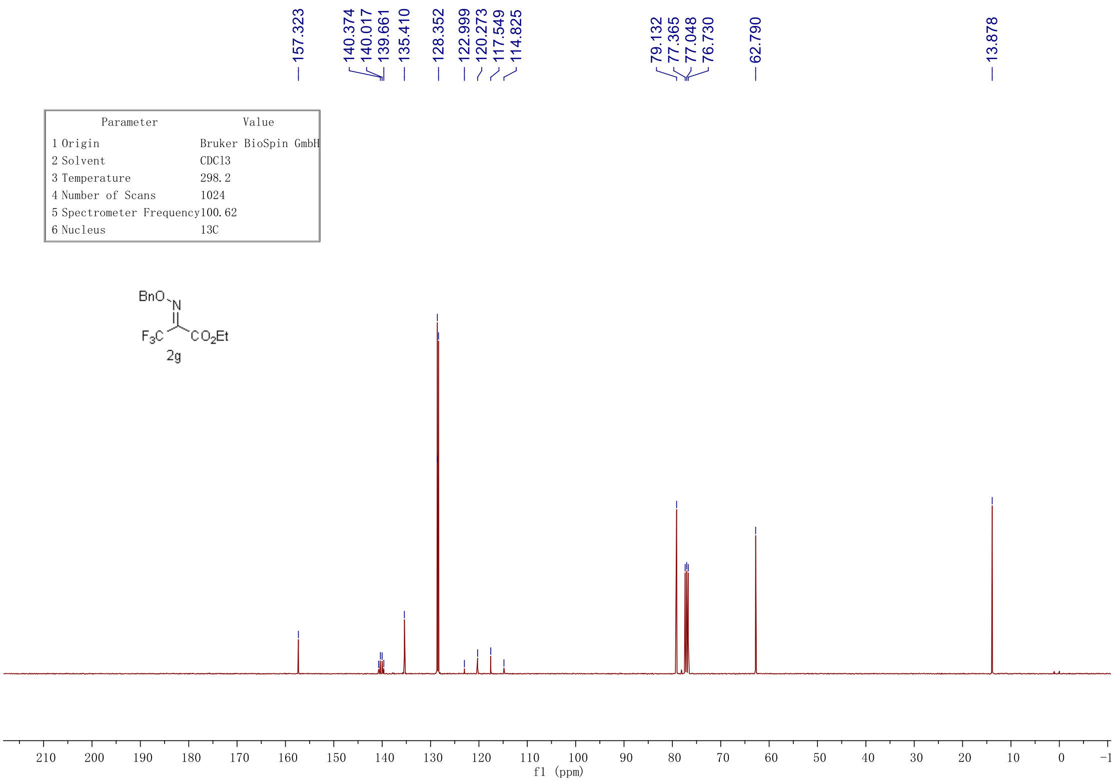
157.323
140.374
140.017
139.661
135.410
128.352
122.999
120.273
117.549
114.825
79.132
77.365
77.048
76.730
62.790
13.878
Parameter Value
1 Origin Bruker BioSpin GmbH
2 Solvent CDCl3
3 Temperature 298.2
4 Number of Scans 1024
5 Spectrometer Frequency 100.62
6 Nucleus 13C
BnO
N
F3C
CO2Et
2g
f1 (ppm)

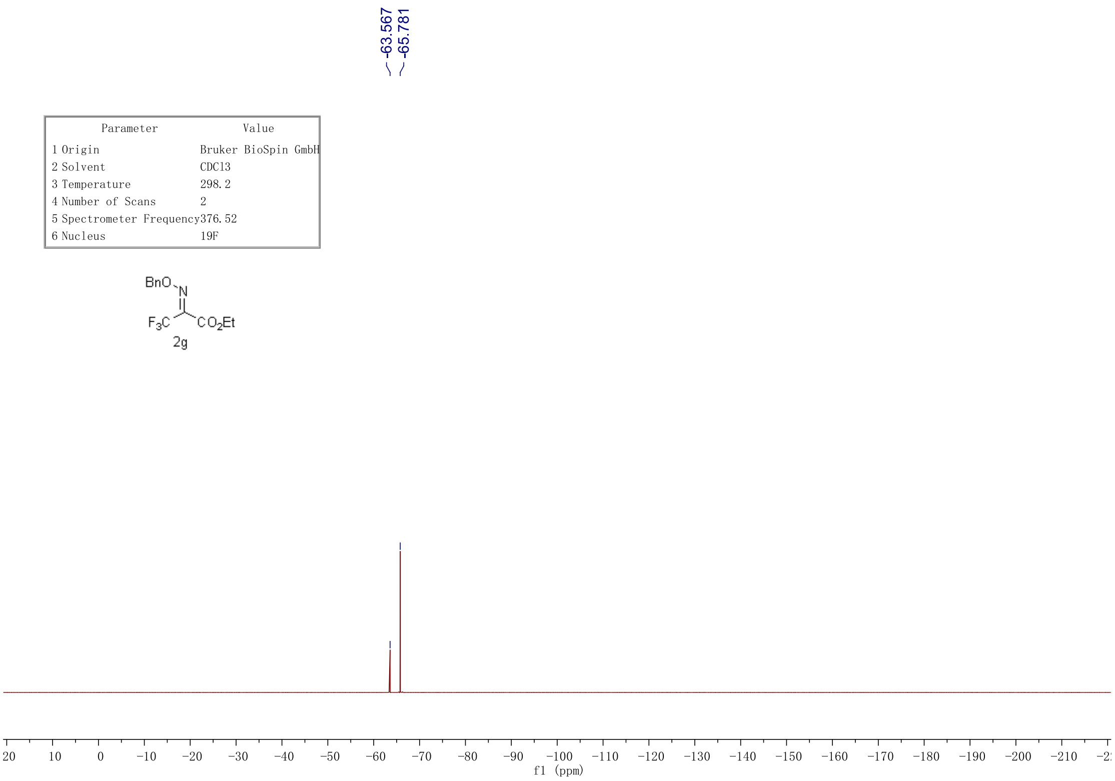
-63.567
-65.781
Parameter Value
1 Origin Bruker BioSpin GmbH
2 Solvent CDCl3
3 Temperature 298.2
4 Number of Scans 2
5 Spectrometer Frequency 376.52
6 Nucleus 19F
BnO
N
F3C
CO2Et
2g
f1 (ppm)

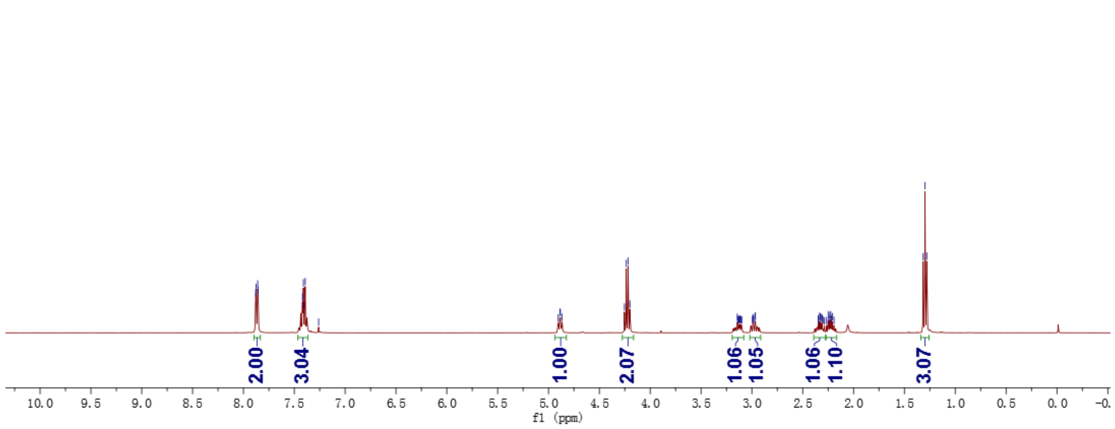

Parameter	Value
1 Origin	Bruker BioSpin GmbH
2 Solvent	CDCl3
3 Temperature	298.1
4 Number of Scans	2
5 Spectrometer Frequency	400.13
6 Nucleus	1H

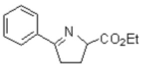

3a

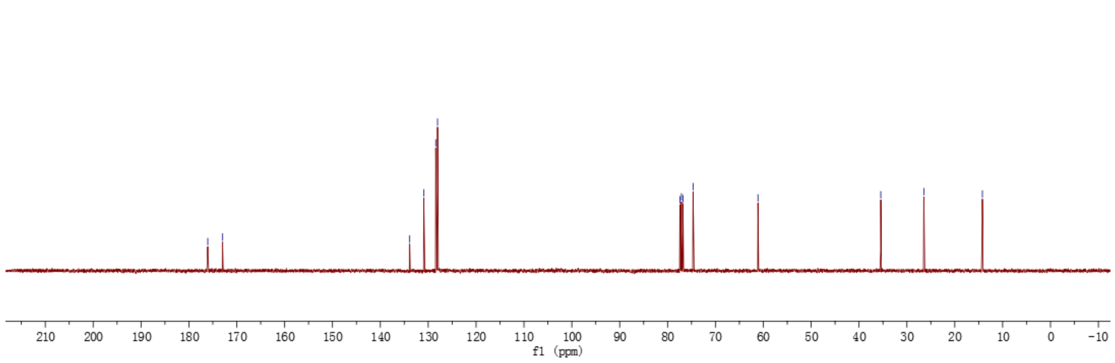

Parameter	Value
1 Origin	Bruker BioSpin GmbH
2 Solvent	CDCl3
3 Temperature	298.4
4 Number of Scans	41
5 Spectrometer Frequency	100.62
6 Nucleus	13C

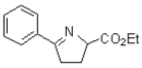

3a

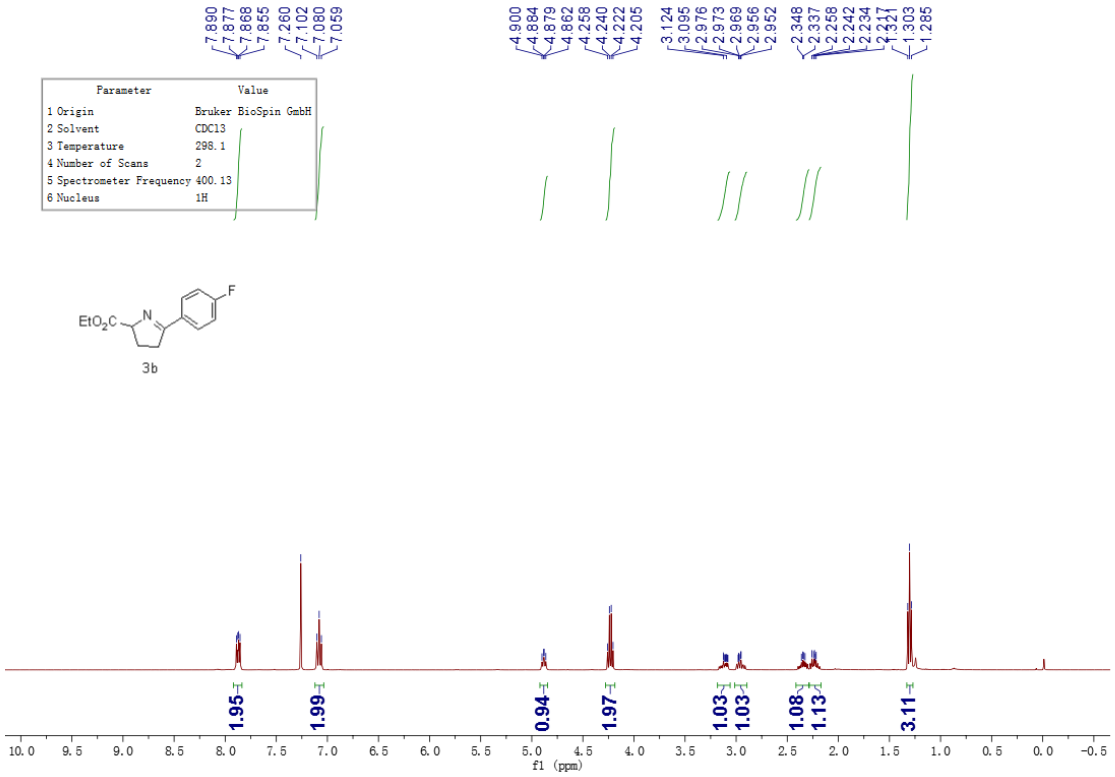
7.890 7.877 7.868 7.855 7.260 7.102 7.080 7.059
4.900 4.884 4.879 4.862 4.258 4.240 4.222 4.205 3.124 3.095 2.976 2.973 2.969 2.956 2.952 2.348 2.337 2.258 2.242 2.234 2.217 1.321 1.303 1.285
Parameter Value
1 Origin Bruker BioSpin GmbH
2 Solvent CDCl3
3 Temperature 298.1
4 Number of Scans 2
5 Spectrometer Frequency 400.13
6 Nucleus 1H
EtO2C
N
F
3b
1.95 1.99 0.94 1.97 1.03 1.03 1.08 1.13 3.11
f1 (ppm)

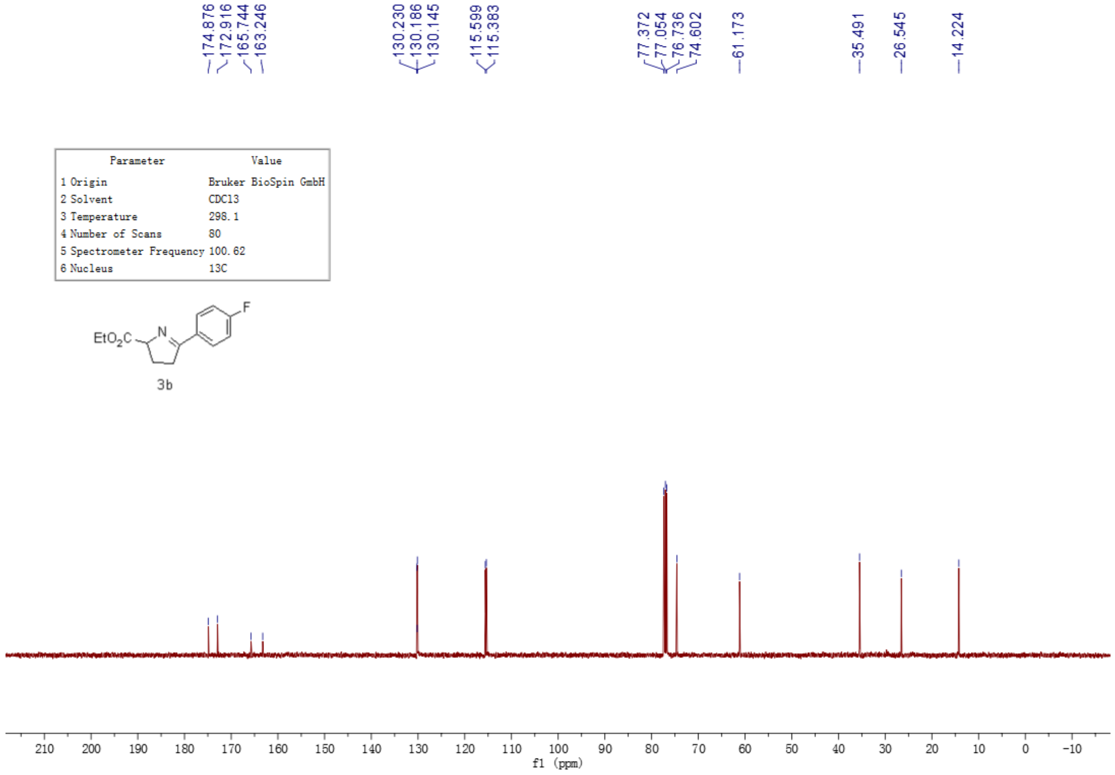
174.876 172.916 165.744 163.246 130.230 130.186 130.145 115.599 115.383 77.372 77.054 76.736 74.602 61.173 35.491 26.545 14.224
Parameter Value
1 Origin Bruker BioSpin GmbH
2 Solvent CDCl3
3 Temperature 298.1
4 Number of Scans 80
5 Spectrometer Frequency 100.62
6 Nucleus 13C
EtO2C
N
F
3b
f1 (ppm)

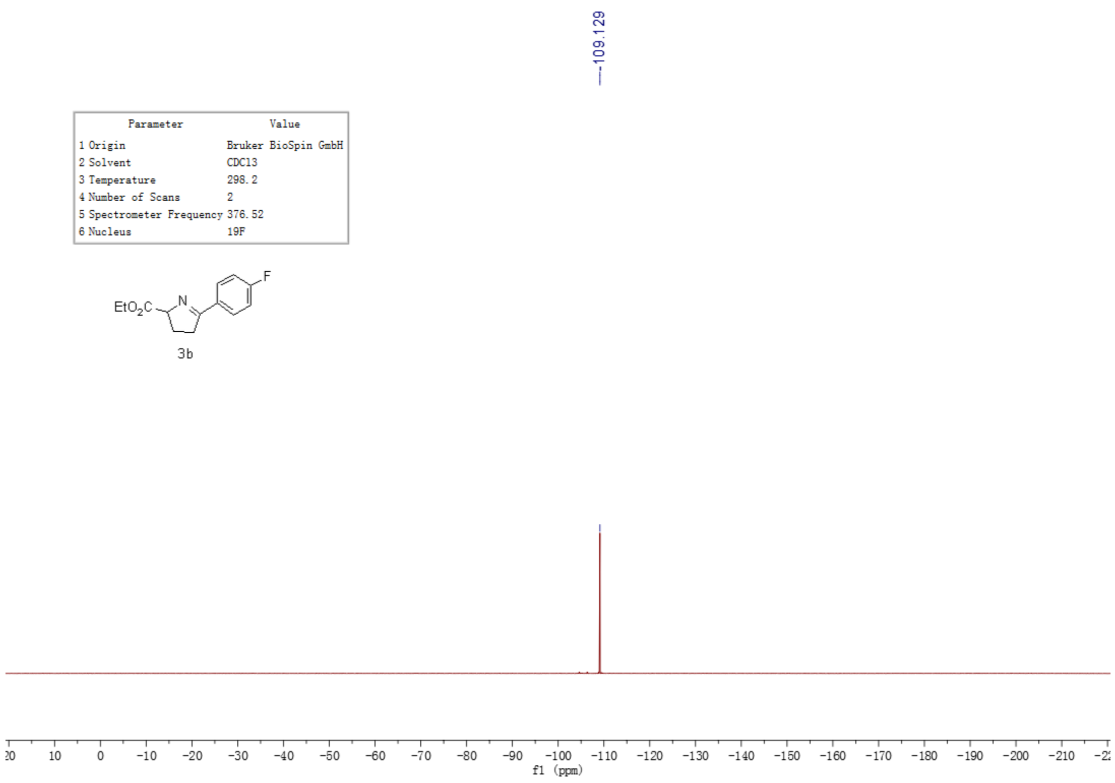
-109.129
Parameter Value
1 Origin Bruker BioSpin GmbH
2 Solvent CDCl3
3 Temperature 298.2
4 Number of Scans 2
5 Spectrometer Frequency 376.52
6 Nucleus 19F
EtO2C
N
F
3b
f1 (ppm)

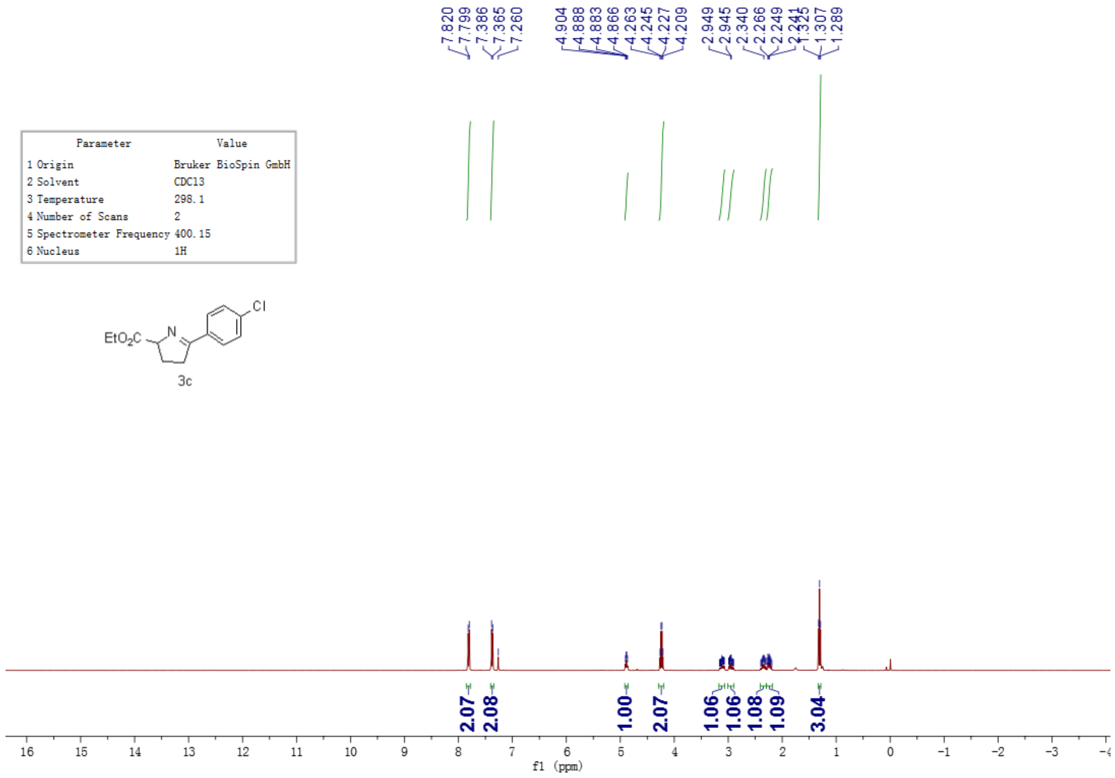
7.820
7.799
7.386
7.365
7.260
4.904
4.888
4.883
4.866
4.263
4.245
4.227
4.209
2.949
2.945
2.340
2.266
2.249
1.307
1.289
Parameter Value
1 Origin Bruker BioSpin GmbH
2 Solvent CDCl3
3 Temperature 298.1
4 Number of Scans 2
5 Spectrometer Frequency 400.15
6 Nucleus 1H
EtO2C
N
Cl
3c
2.07
2.08
1.00
2.07
1.06
1.06
1.08
1.09
3.04
f1 (ppm)

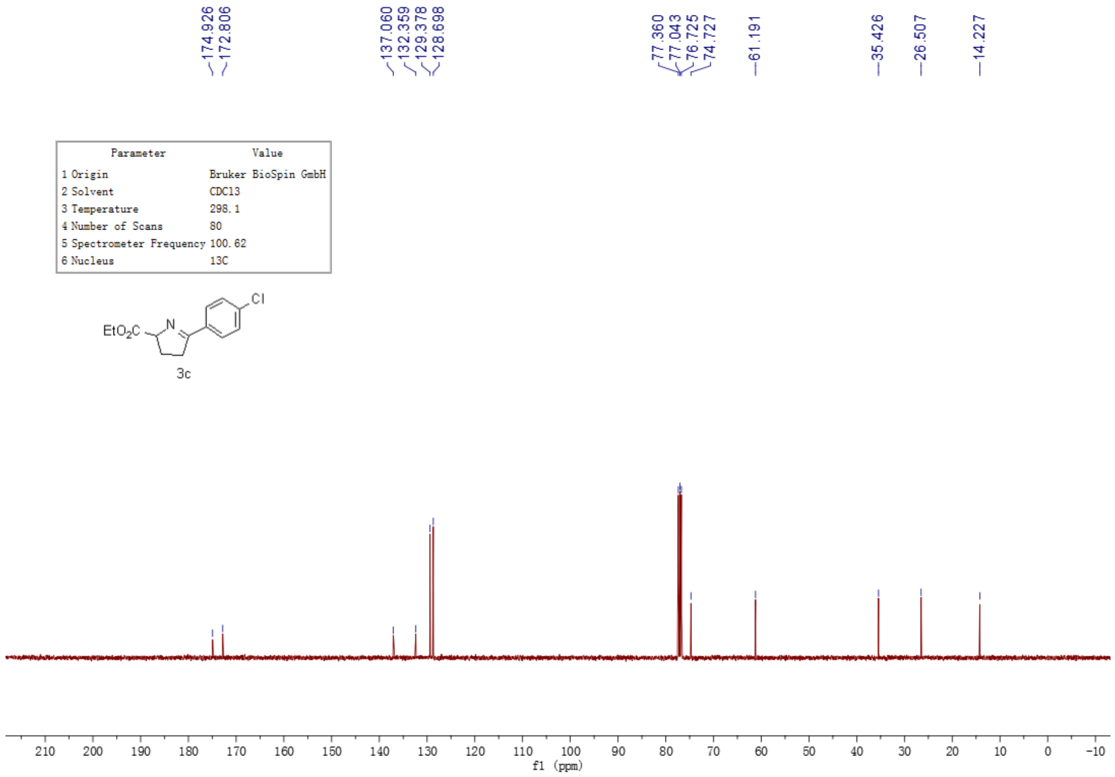
174.926
172.806
137.060
132.359
129.378
128.698
77.360
77.043
76.725
74.727
61.191
35.426
26.507
14.227
Parameter Value
1 Origin Bruker BioSpin GmbH
2 Solvent CDCl3
3 Temperature 298.1
4 Number of Scans 80
5 Spectrometer Frequency 100.62
6 Nucleus 13C
EtO2C
N
Cl
3c
f1 (ppm)

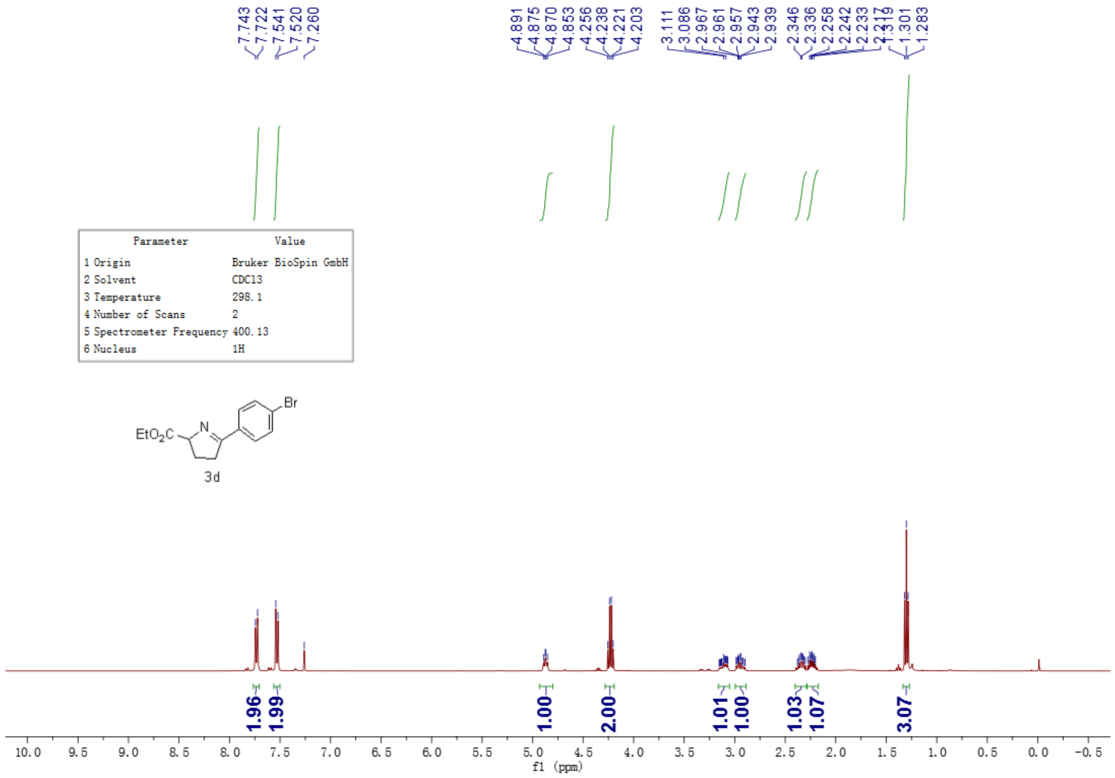
7.743
7.722
7.541
7.520
7.260
4.891
4.875
4.870
4.853
4.256
4.238
4.221
4.203
3.111
3.086
2.967
2.961
2.957
2.943
2.939
2.346
2.336
2.258
2.242
2.233
1.301
1.283
Parameter Value
1 Origin Bruker BioSpin GmbH
2 Solvent CDCl3
3 Temperature 298.1
4 Number of Scans 2
5 Spectrometer Frequency 400.13
6 Nucleus 1H
EtO2C
N
Br
3d
1.96
1.99
1.00
2.00
1.01
1.00
1.03
1.07
3.07
f1 (ppm)

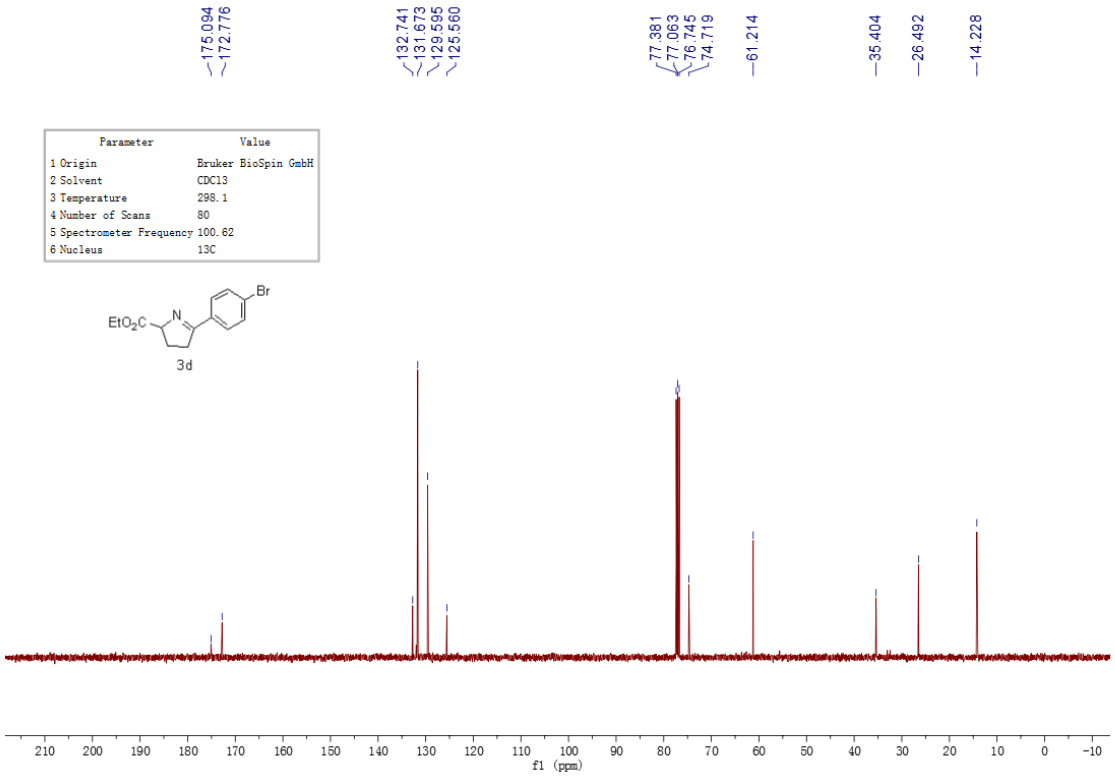
175.094
172.776
132.741
131.673
129.595
125.560
77.381
77.063
76.745
74.719
61.214
35.404
26.492
14.228
Parameter Value
1 Origin Bruker BioSpin GmbH
2 Solvent CDCl3
3 Temperature 298.1
4 Number of Scans 80
5 Spectrometer Frequency 100.62
6 Nucleus 13C
EtO2C
N
Br
3d
210 200 190 180 170 160 150 140 130 120 110 100 90 80 70 60 50 40 30 20 10 0 -10
f1 (ppm)

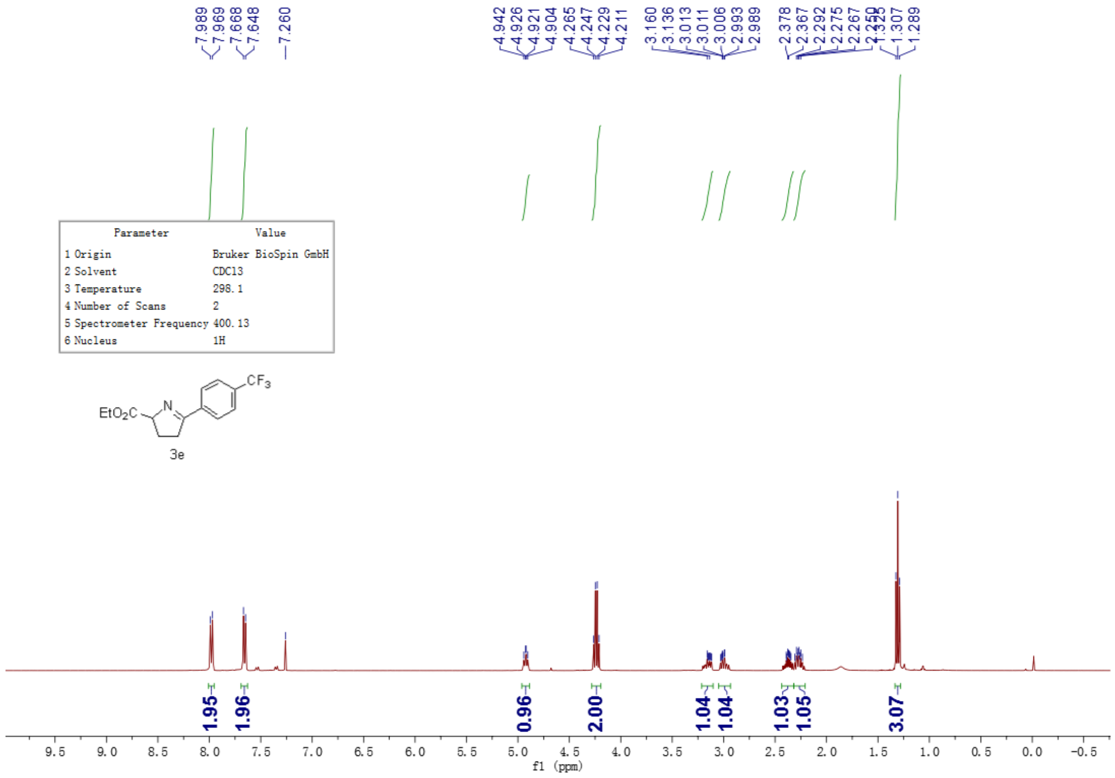
7.989
7.969
7.668
7.648
7.260
4.942
4.926
4.921
4.904
4.265
4.247
4.229
4.211
3.160
3.136
3.013
3.011
3.006
2.993
2.989
2.378
2.367
2.292
2.275
2.267
1.307
1.289
Parameter Value
1 Origin Bruker BioSpin GmbH
2 Solvent CDCl3
3 Temperature 298.1
4 Number of Scans 2
5 Spectrometer Frequency 400.13
6 Nucleus 1H
EtO2C
N
CF3
3e
1.95
1.96
0.96
2.00
1.04
1.04
1.03
1.05
3.07
9.5 9.0 8.5 8.0 7.5 7.0 6.5 6.0 5.5 5.0 4.5 4.0 3.5 3.0 2.5 2.0 1.5 1.0 0.5 0.0 -0.5
f1 (ppm)

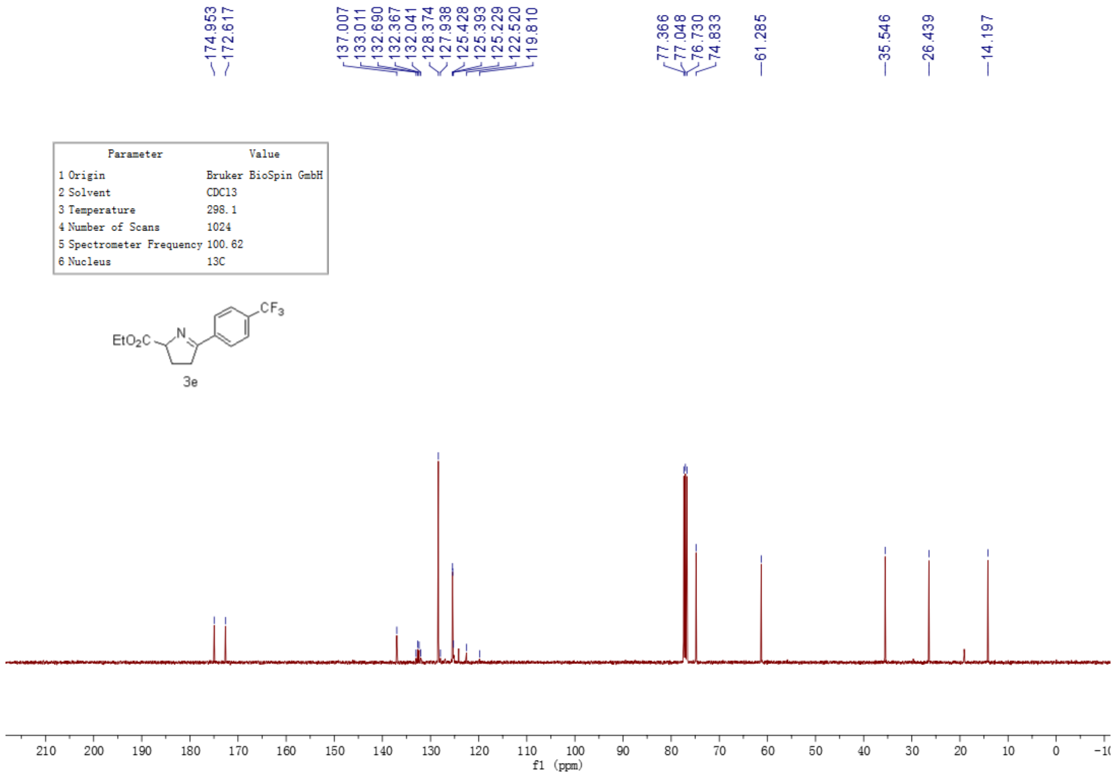
174.953
172.617
137.007
133.011
132.690
132.367
132.041
128.374
127.938
125.428
125.393
125.229
122.520
119.810
77.366
77.048
76.730
74.833
61.285
35.546
26.439
14.197
Parameter Value
1 Origin Bruker BioSpin GmbH
2 Solvent CDCl3
3 Temperature 298.1
4 Number of Scans 1024
5 Spectrometer Frequency 100.62
6 Nucleus 13C
EtO2C
N
CF3
3e
f1 (ppm)

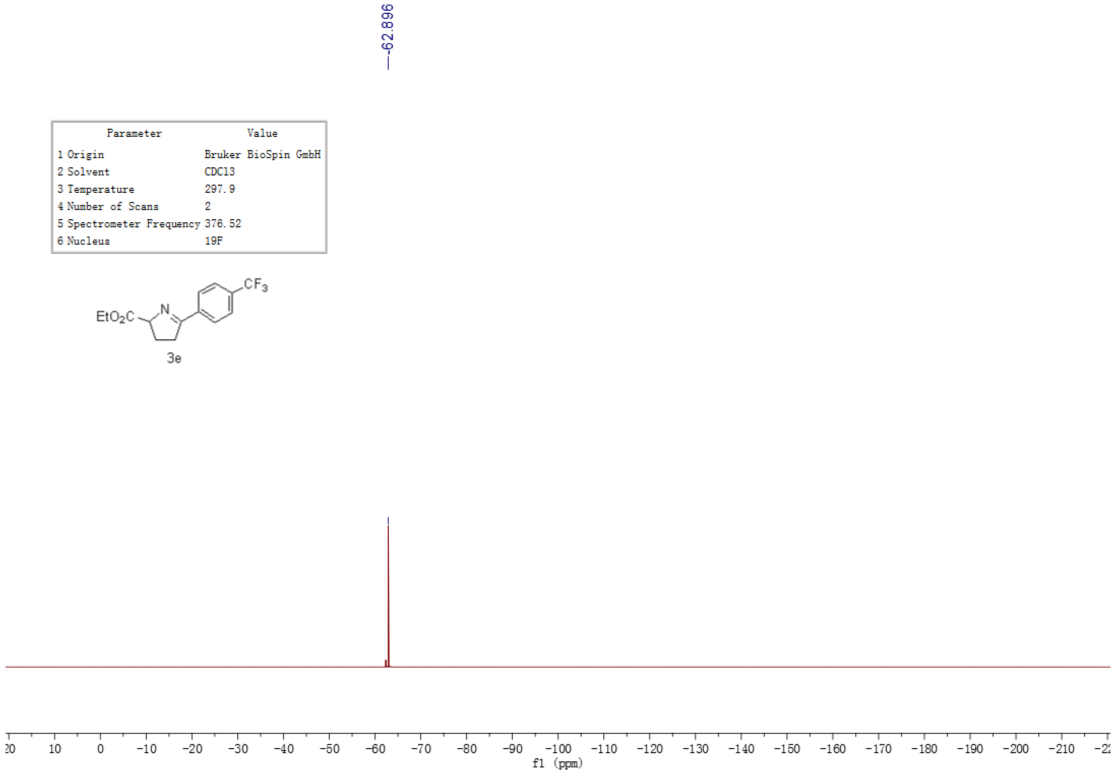
-62.896
Parameter Value
1 Origin Bruker BioSpin GmbH
2 Solvent CDCl3
3 Temperature 297.9
4 Number of Scans 2
5 Spectrometer Frequency 376.52
6 Nucleus 19F
EtO2C
N
CF3
3e
f1 (ppm)

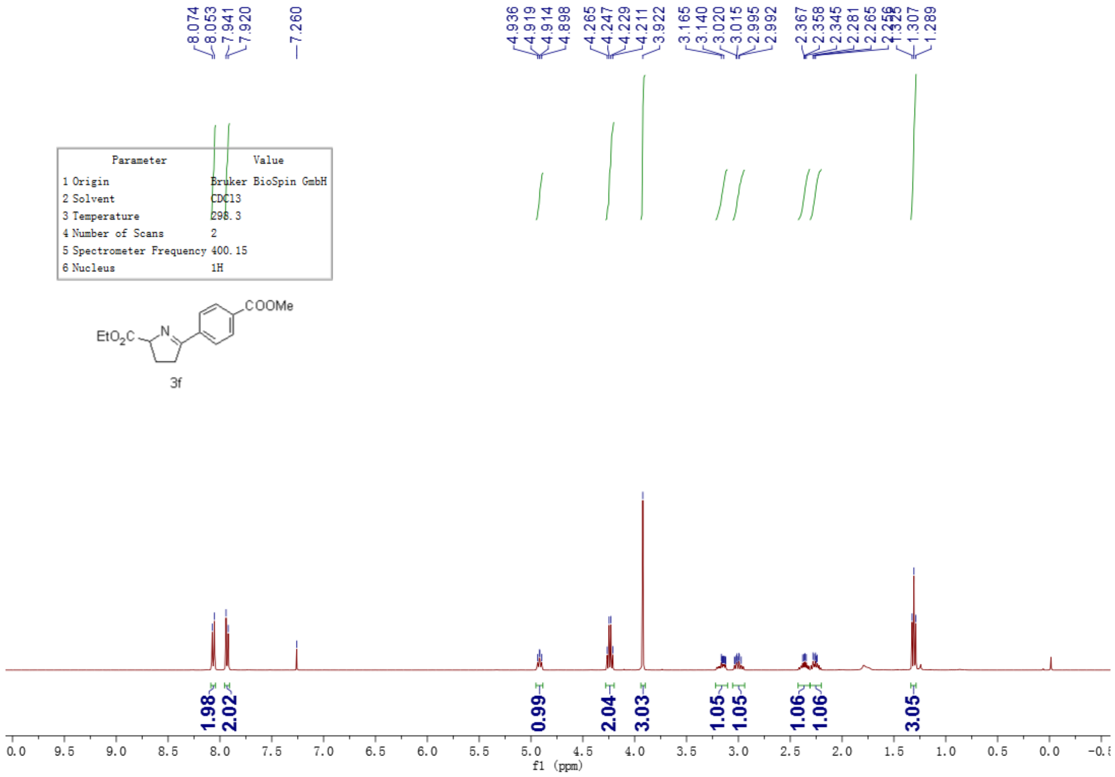
8.074
8.053
7.941
7.920
7.260
4.936
4.919
4.914
4.898
4.265
4.247
4.229
4.211
3.922
3.165
3.140
3.020
3.015
2.995
2.992
2.367
2.358
2.345
2.281
2.265
1.325
1.307
1.289
Parameter Value
1 Origin Bruker BioSpin GmbH
2 Solvent CDCl3
3 Temperature 298.3
4 Number of Scans 2
5 Spectrometer Frequency 400.15
6 Nucleus 1H
COOMe
EtO2C
N
3f
1.98
2.02
0.99
2.04
3.03
1.05
1.05
1.06
1.06
3.05
f1 (ppm)

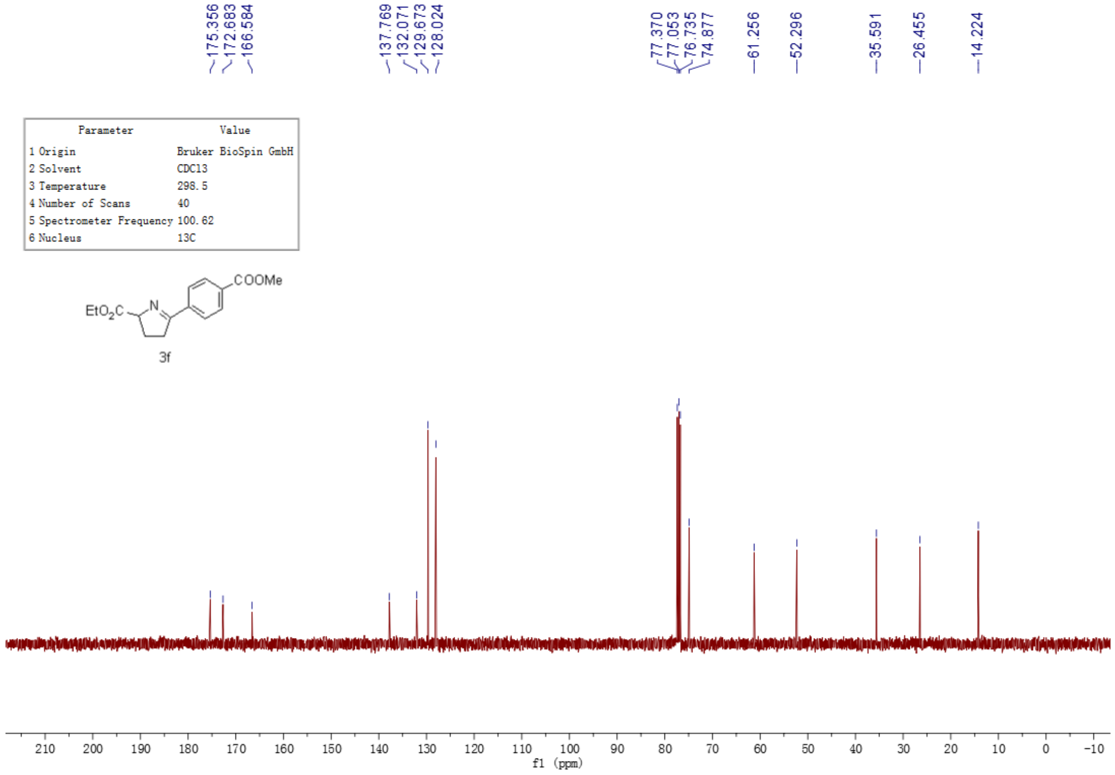
175.356
172.683
166.584
137.769
132.071
129.673
128.024
77.370
77.053
76.735
74.877
61.256
52.296
35.591
26.455
14.224
Parameter Value
1 Origin Bruker BioSpin GmbH
2 Solvent CDCl3
3 Temperature 298.5
4 Number of Scans 40
5 Spectrometer Frequency 100.62
6 Nucleus 13C
COOMe
EtO2C
N
3f
f1 (ppm)

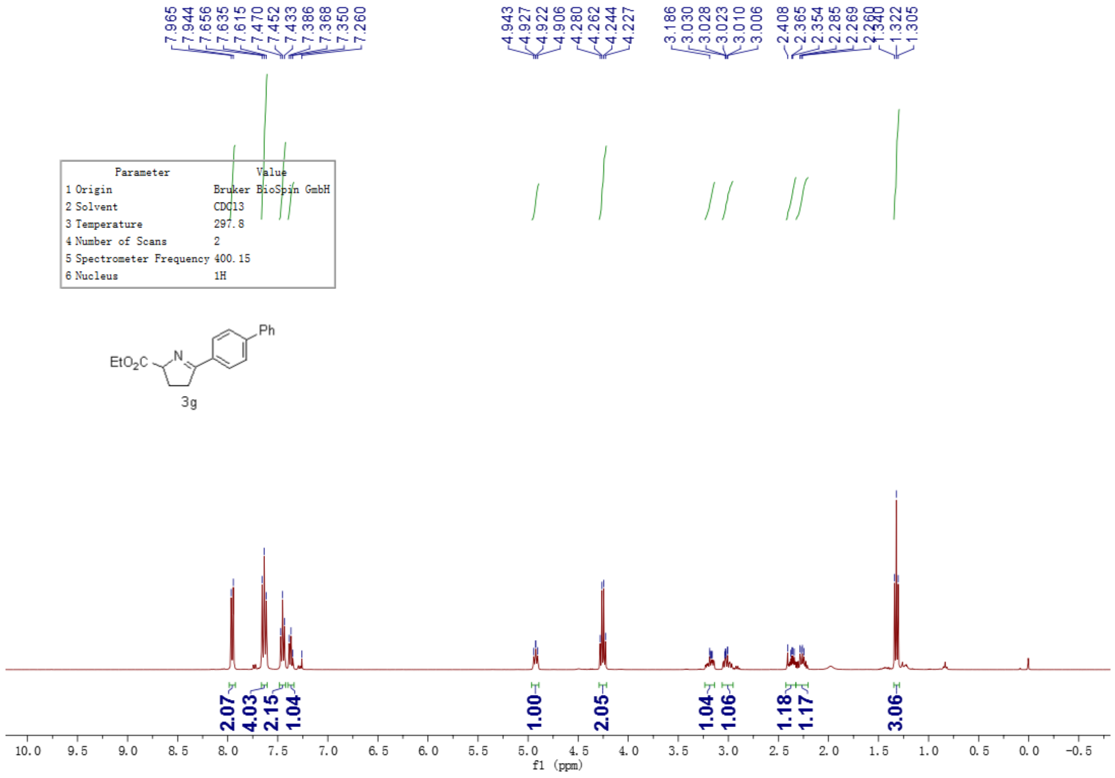
Parameter Value
1 Origin Bruker BioSpin GmbH
2 Solvent CDCl3
3 Temperature 297.8
4 Number of Scans 2
5 Spectrometer Frequency 400.15
6 Nucleus 1H
EtO2C
N
Ph
3g
f1 (ppm)

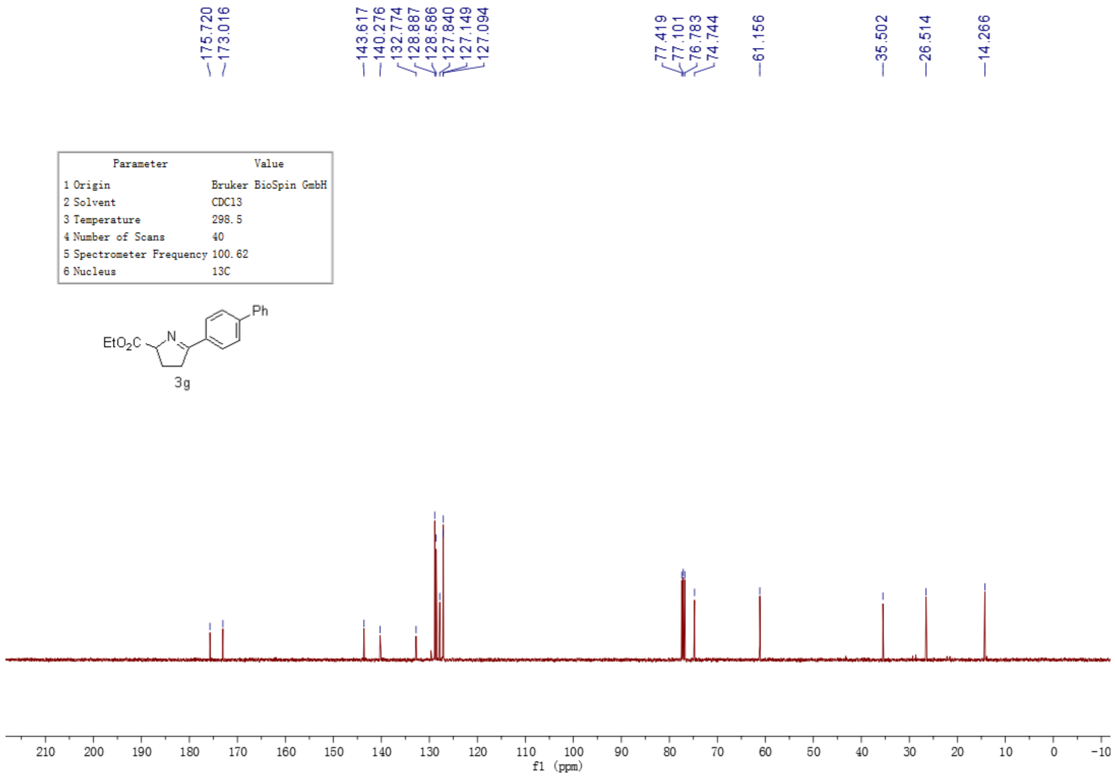
Parameter Value
1 Origin Bruker BioSpin GmbH
2 Solvent CDCl3
3 Temperature 298.5
4 Number of Scans 40
5 Spectrometer Frequency 100.62
6 Nucleus 13C
EtO2C
N
Ph
3g
175.720
173.016
143.617
140.276
132.774
128.887
128.586
127.840
127.149
127.094
77.419
77.101
76.783
74.744
61.156
35.502
26.514
14.266
f1 (ppm)

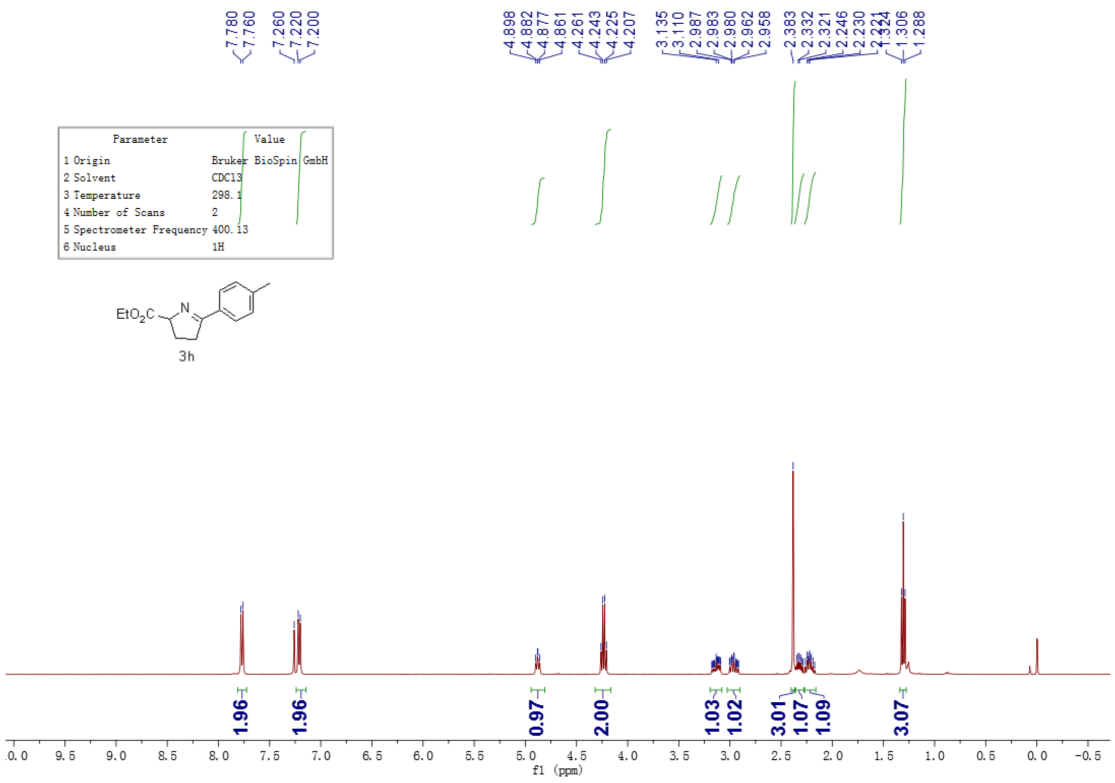
7.780
7.760
7.260
7.220
7.200
4.898
4.882
4.877
4.861
4.261
4.243
4.225
4.207
3.135
3.110
2.987
2.983
2.980
2.962
2.958
2.383
2.332
2.321
2.246
2.230
2.221
1.324
1.306
1.288
Parameter | Value
1 Origin | Bruker BioSpin GmbH
2 Solvent | CDCl3
3 Temperature | 298.1
4 Number of Scans | 2
5 Spectrometer Frequency | 400.13
6 Nucleus | 1H
EtO2C
N
3h
1.96
1.96
0.97
2.00
1.03
1.02
3.01
1.07
1.09
3.07
f1 (ppm)

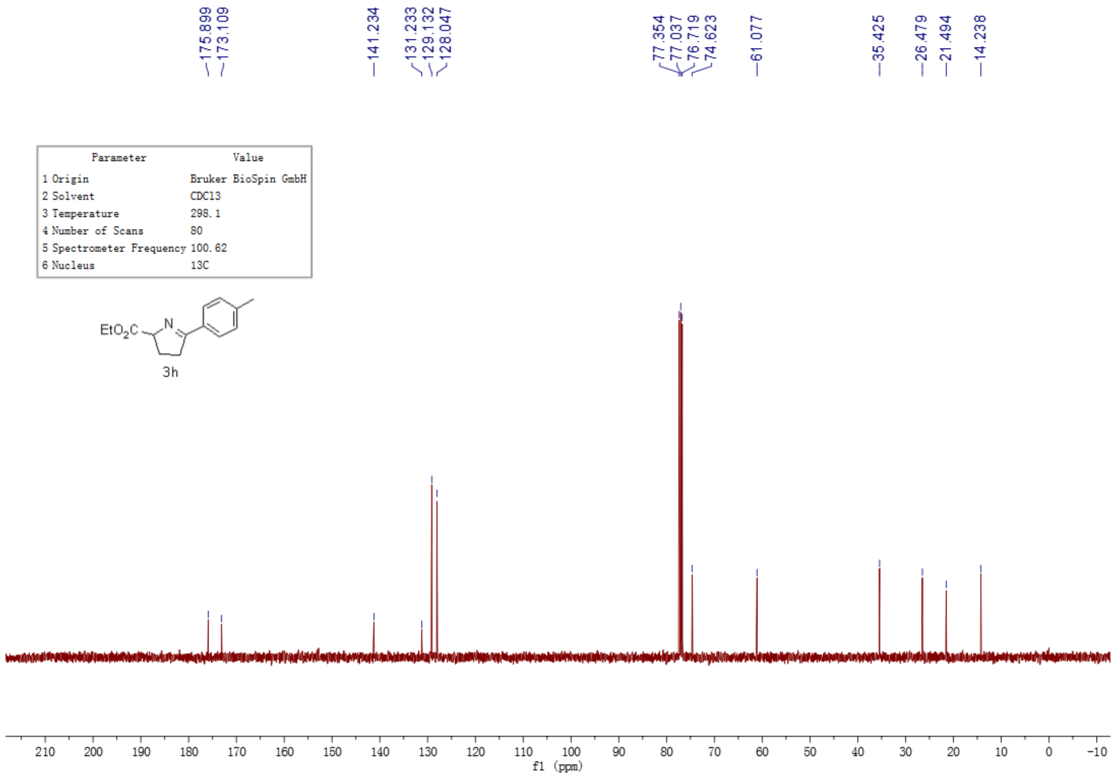
175.899
173.109
141.234
131.233
129.132
128.047
77.354
77.037
76.719
74.623
61.077
35.425
26.479
21.494
14.238
Parameter | Value
1 Origin | Bruker BioSpin GmbH
2 Solvent | CDCl3
3 Temperature | 298.1
4 Number of Scans | 80
5 Spectrometer Frequency | 100.62
6 Nucleus | 13C
EtO2C
N
3h
f1 (ppm)

Parameter	Value
1 Origin	Bruker BioSpin GmbH
2 Solvent	CDCl3
3 Temperature	298.2
4 Number of Scans	2
5 Spectrometer Frequency	400.13
6 Nucleus	1H

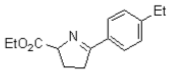

3i

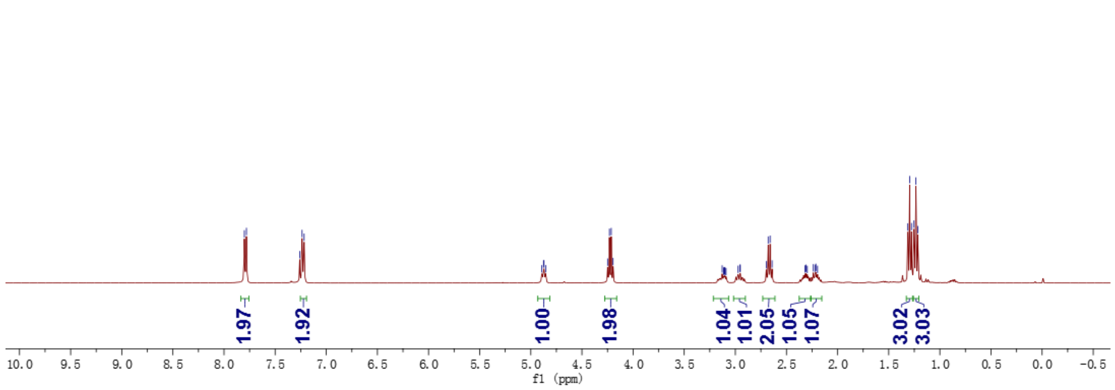

Parameter	Value
1 Origin	Bruker BioSpin GmbH
2 Solvent	CDCl3
3 Temperature	298.1
4 Number of Scans	80
5 Spectrometer Frequency	100.62
6 Nucleus	13C

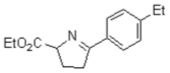

3i

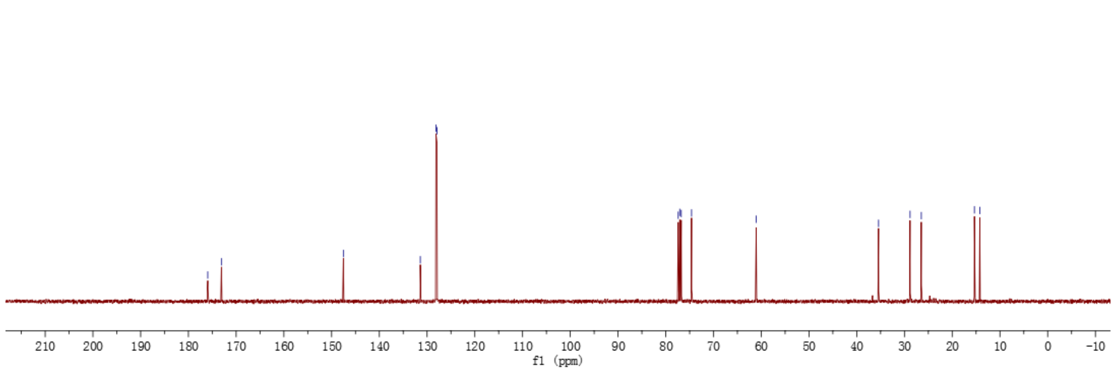

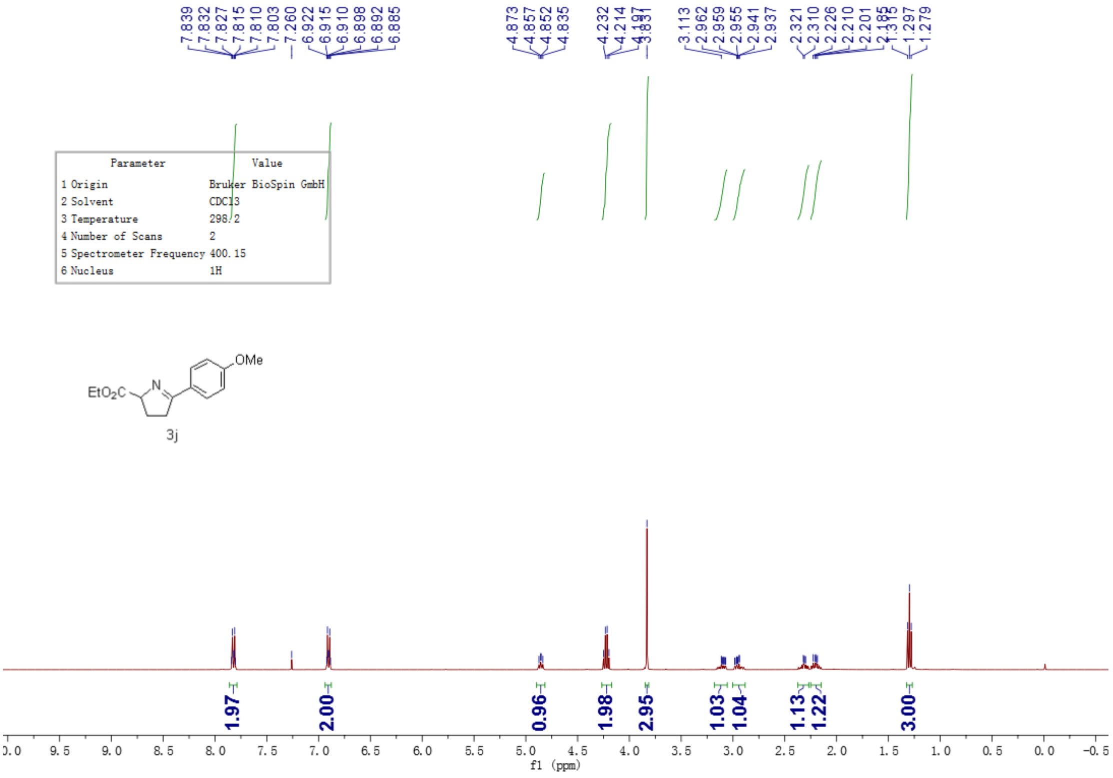

	Parameter	Value
1	Origin	Bruker BioSpin GmbH
2	Solvent	CDCl3
3	Temperature	298.2
4	Number of Scans	2
5	Spectrometer Frequency	400.15
6	Nucleus	1H

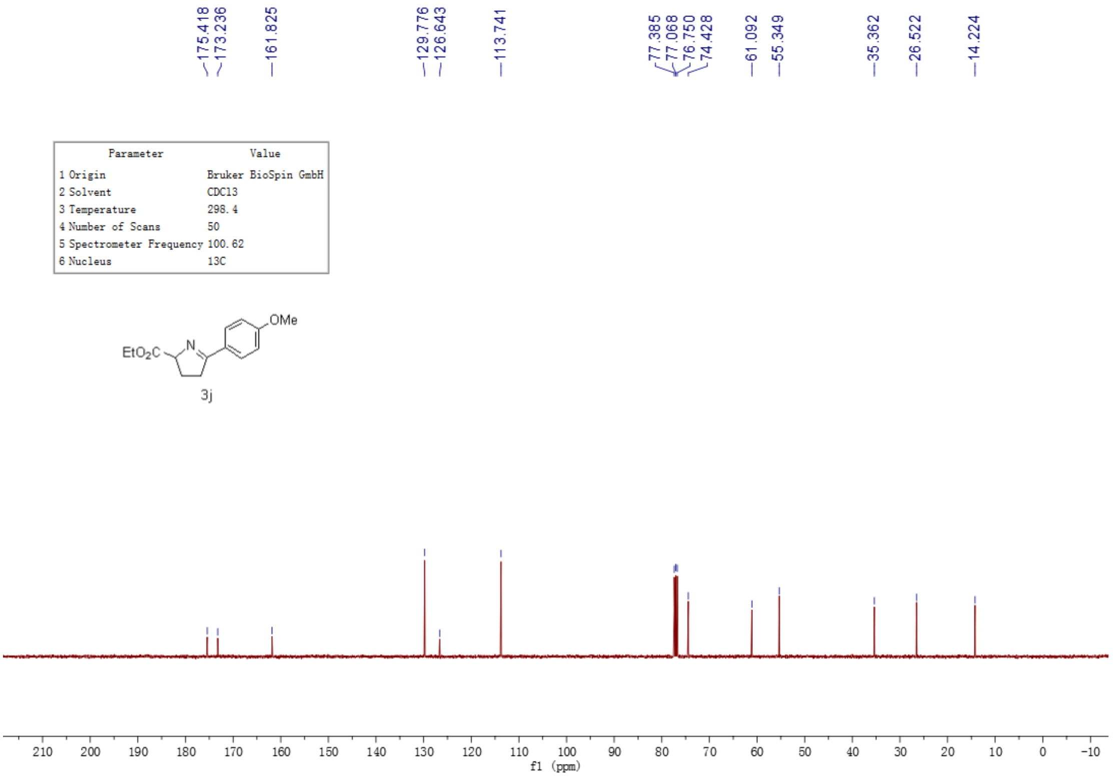

	Parameter	Value
1	Origin	Bruker BioSpin GmbH
2	Solvent	CDCl3
3	Temperature	298.4
4	Number of Scans	50
5	Spectrometer Frequency	100.62
6	Nucleus	13C

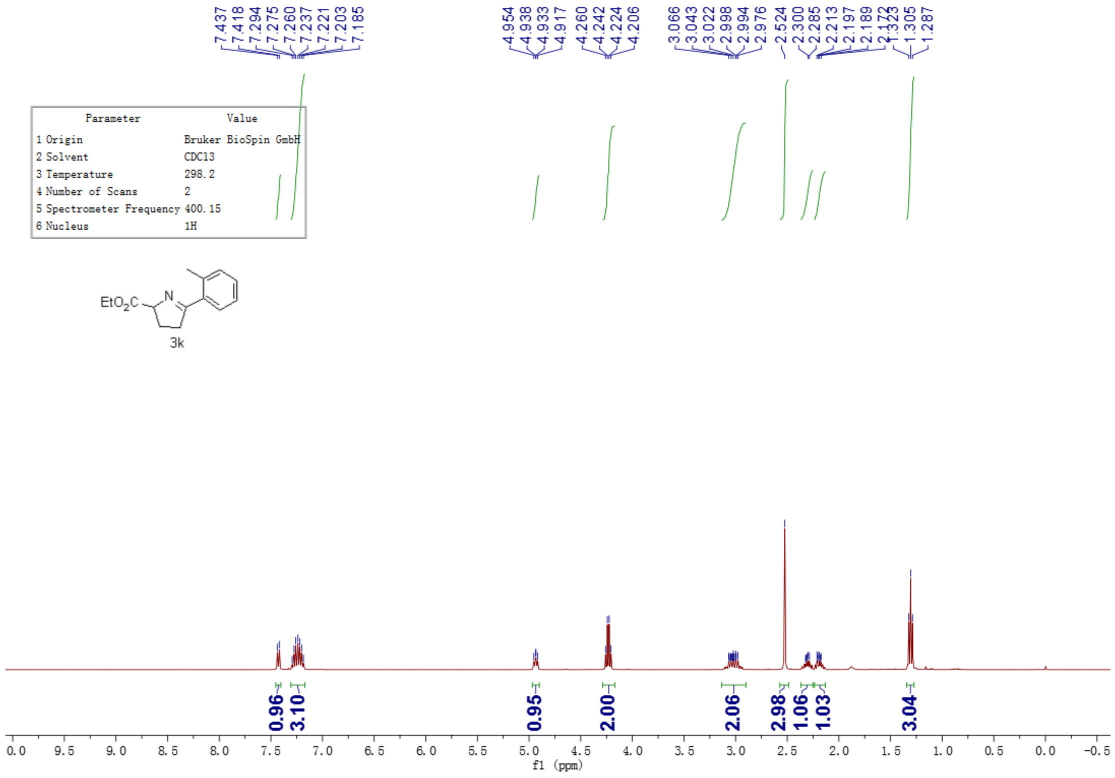

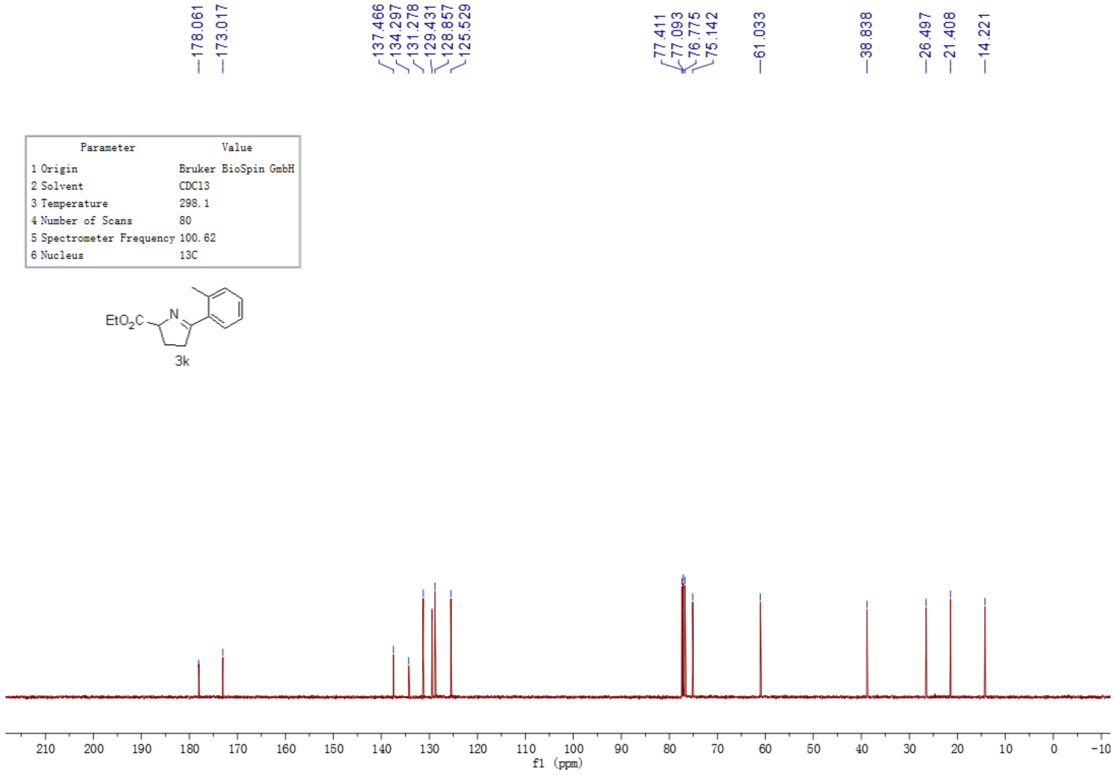

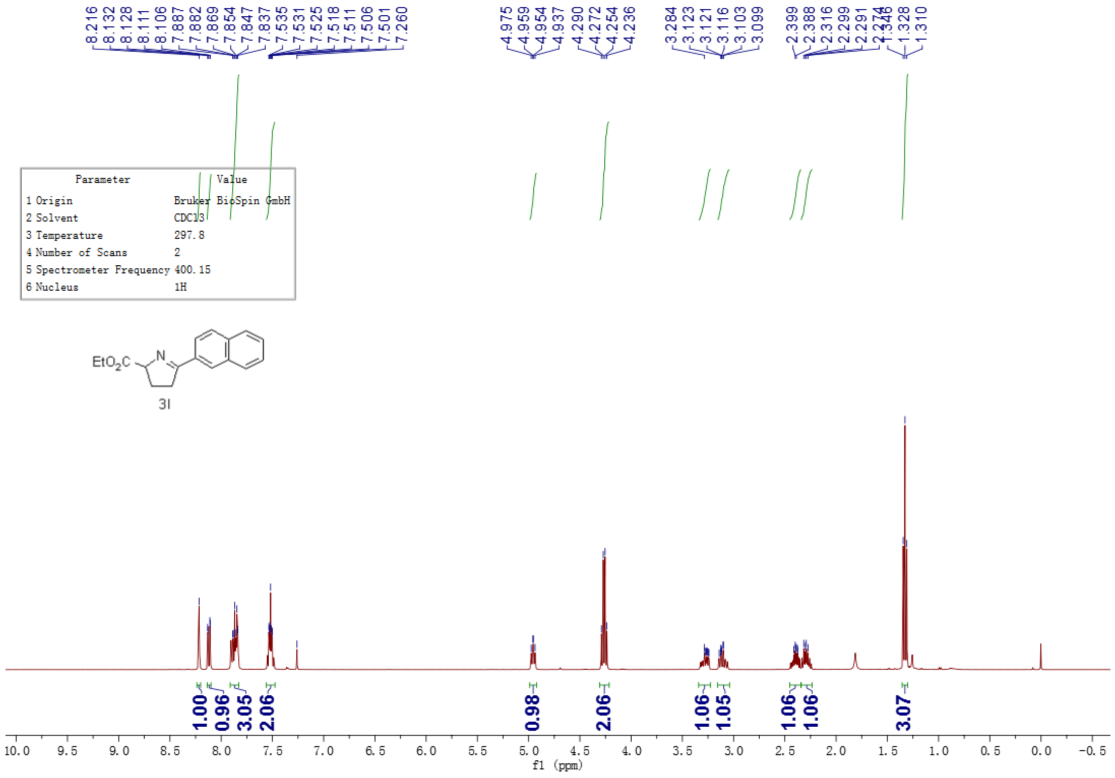

Parameter Value
1 Origin Bruker BioSpin GmbH
2 Solvent CDCl3
3 Temperature 297.8
4 Number of Scans 2
5 Spectrometer Frequency 400.15
6 Nucleus 1H
EtO2C
N
3l
f1 (ppm)

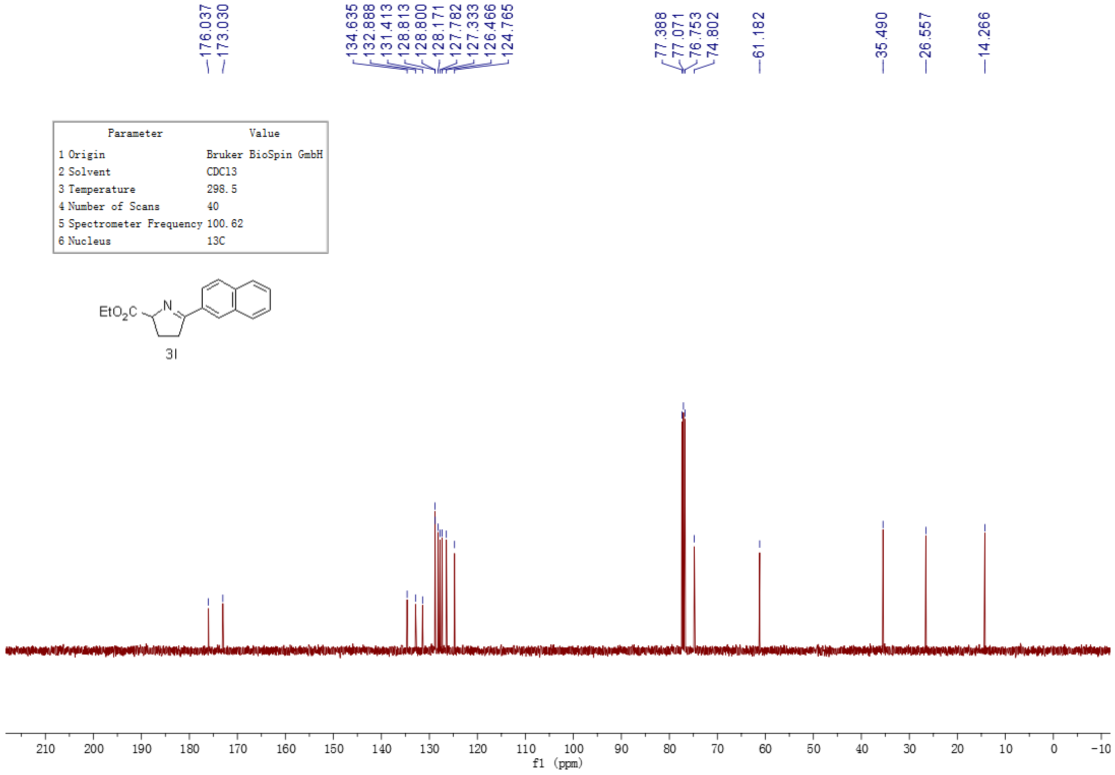

Parameter Value
1 Origin Bruker BioSpin GmbH
2 Solvent CDCl3
3 Temperature 298.5
4 Number of Scans 40
5 Spectrometer Frequency 100.62
6 Nucleus 13C
EtO2C
N
3l
f1 (ppm)

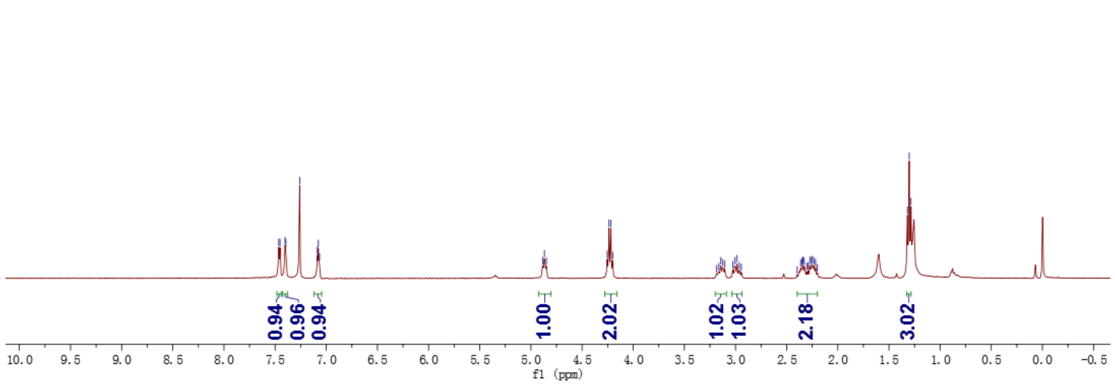

Parameter	Value
1 Origin	Bruker BioSpin GmbH
2 Solvent	CDCl3
3 Temperature	299.1
4 Number of Scans	4
5 Spectrometer Frequency	400.13
6 Nucleus	1H

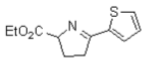

3m

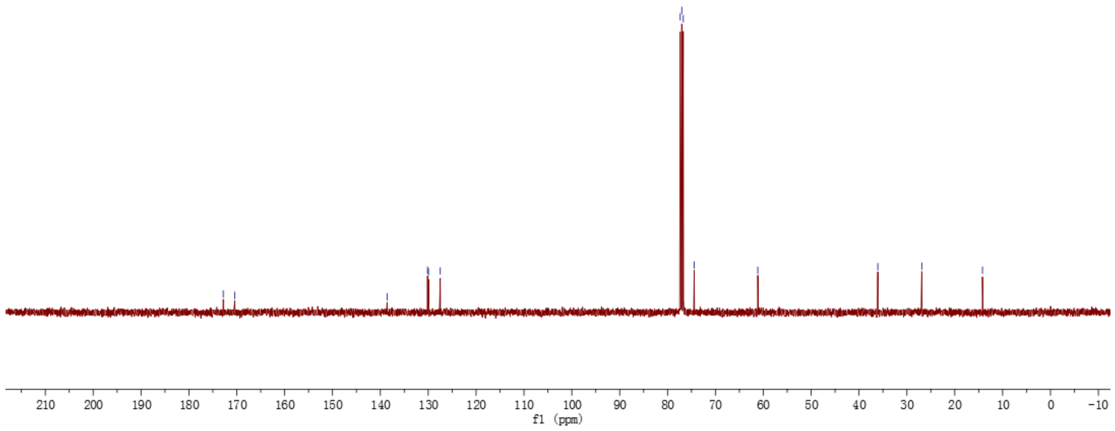

Parameter	Value
1 Origin	Bruker BioSpin GmbH
2 Solvent	CDCl3
3 Temperature	298.1
4 Number of Scans	80
5 Spectrometer Frequency	100.62
6 Nucleus	13C

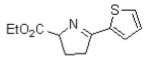

3m

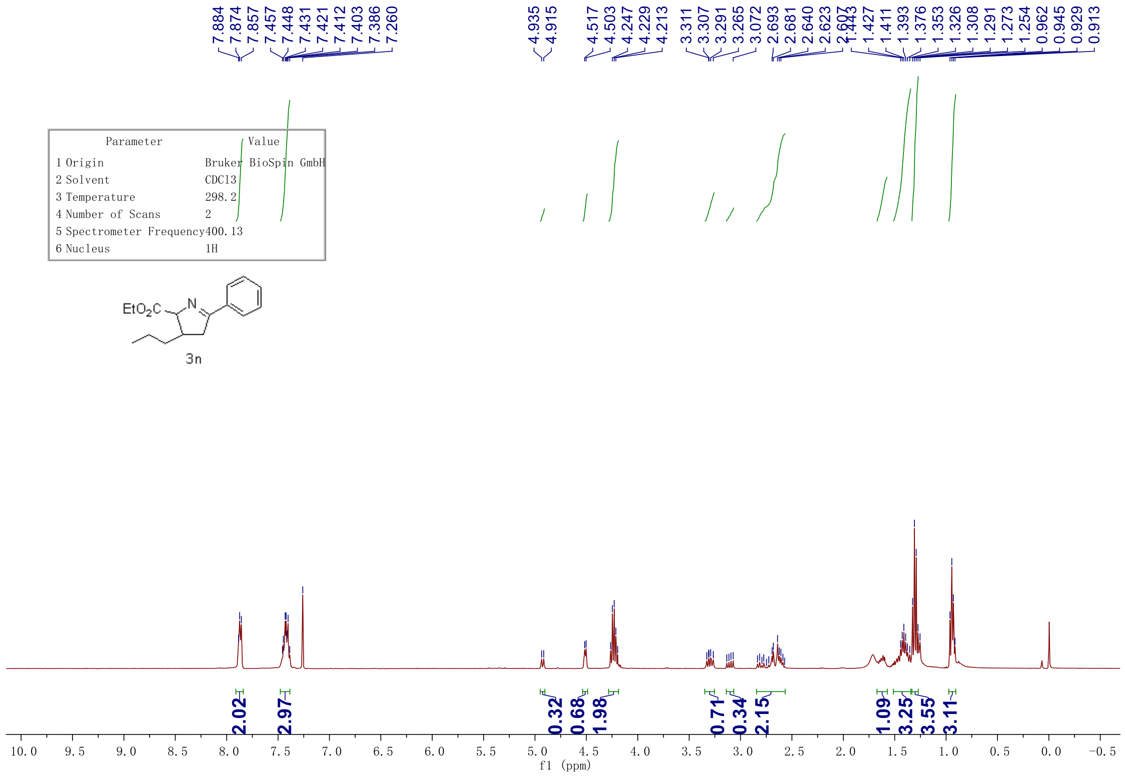

Parameter	Value
1 Origin	Bruker BioSpin GmbH
2 Solvent	CDCl3
3 Temperature	298.2
4 Number of Scans	2
5 Spectrometer Frequency	400.13
6 Nucleus	1H

3n

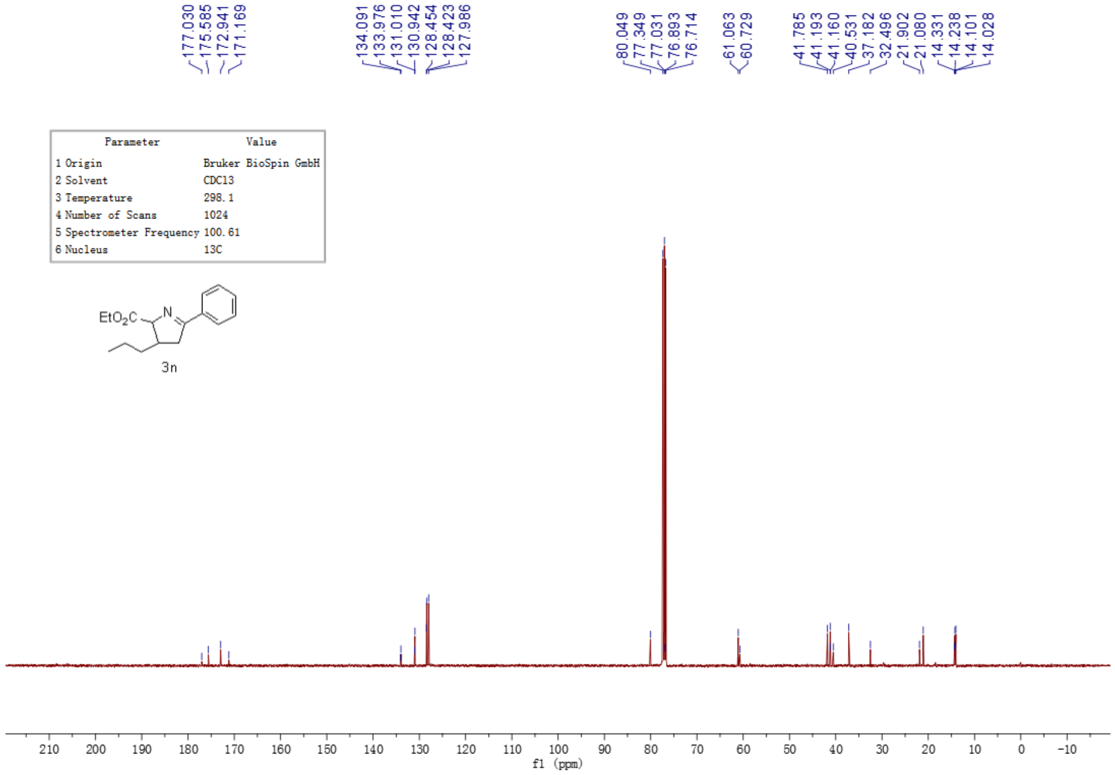

Parameter	Value
1 Origin	Bruker BioSpin GmbH
2 Solvent	CDCl3
3 Temperature	298.1
4 Number of Scans	1024
5 Spectrometer Frequency	100.61
6 Nucleus	13C

3n

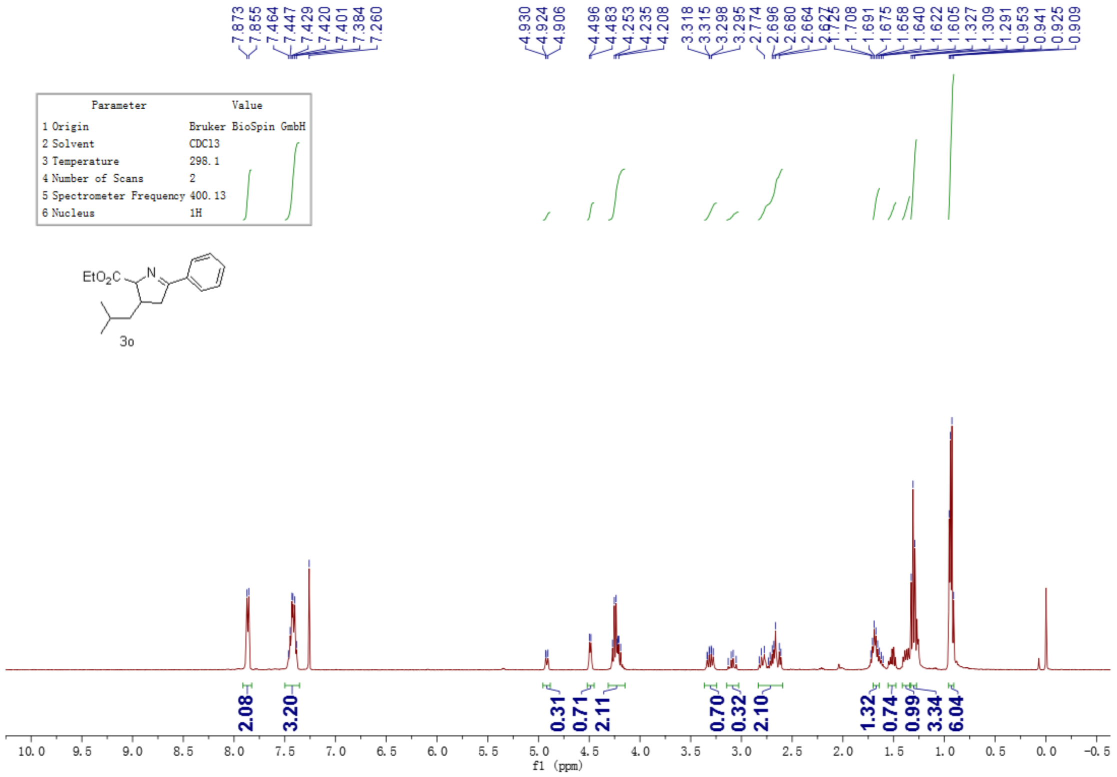
Parameter Value
1 Origin Bruker BioSpin GmbH
2 Solvent CDCl3
3 Temperature 298.1
4 Number of Scans 2
5 Spectrometer Frequency 400.13
6 Nucleus 1H
EtO2C
N
3o
f1 (ppm)

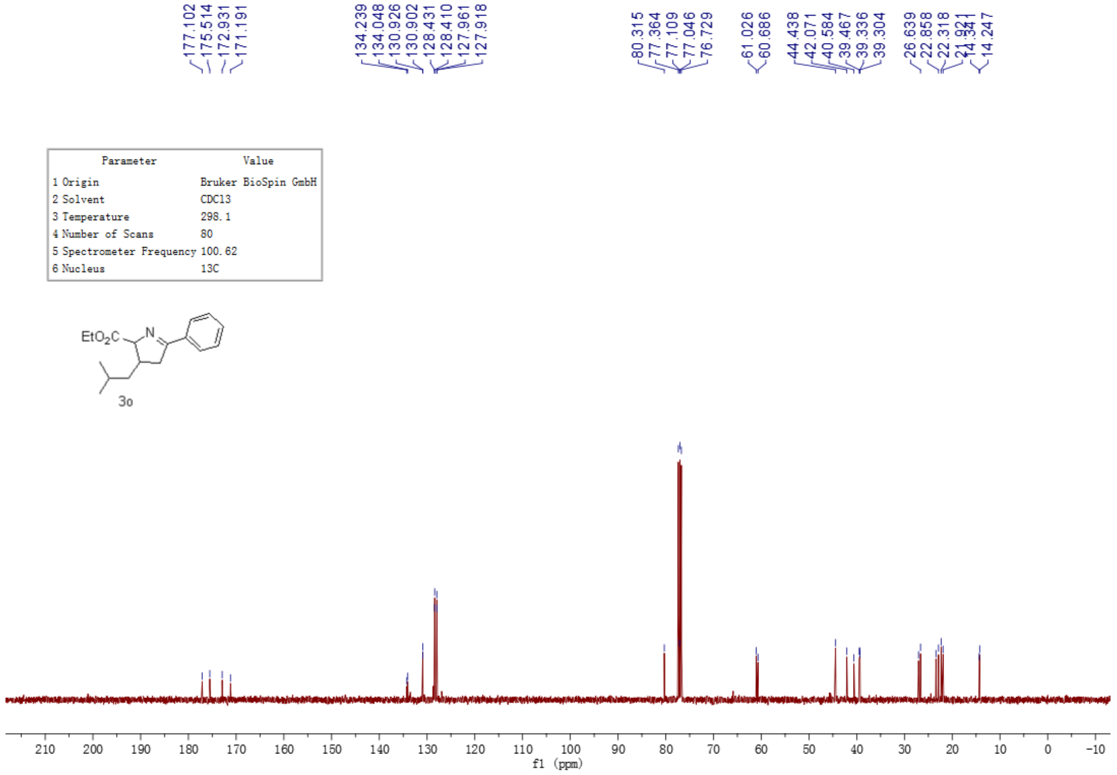
Parameter Value
1 Origin Bruker BioSpin GmbH
2 Solvent CDCl3
3 Temperature 298.1
4 Number of Scans 80
5 Spectrometer Frequency 100.62
6 Nucleus 13C
EtO2C
N
3o
f1 (ppm)

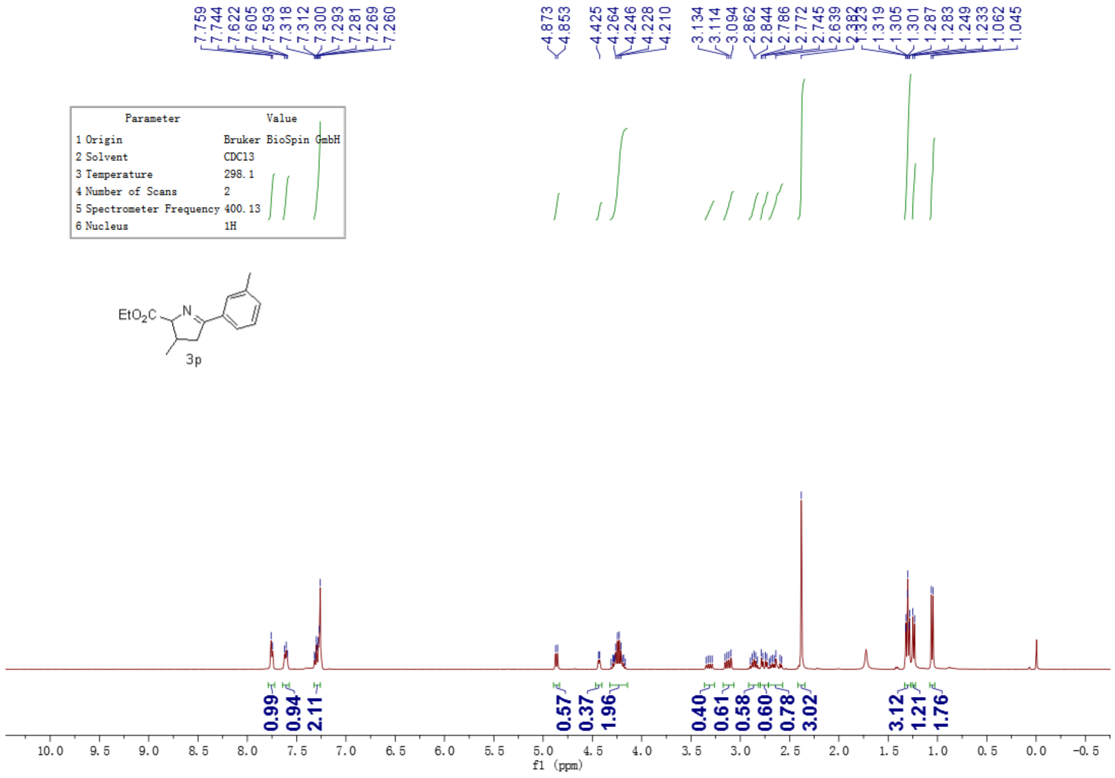
7.759
7.744
7.622
7.605
7.593
7.318
7.312
7.300
7.293
7.281
7.269
7.260
4.873
4.853
4.425
4.264
4.246
4.228
4.210
3.134
3.114
3.094
2.862
2.844
2.786
2.772
2.745
2.639
2.382
1.319
1.305
1.301
1.287
1.283
1.249
1.233
1.062
1.045
Parameter Value
1 Origin Bruker BioSpin GmbH
2 Solvent CDCl3
3 Temperature 298.1
4 Number of Scans 2
5 Spectrometer Frequency 400.13
6 Nucleus 1H
EtO2C
N
3p
0.99
0.94
2.11
0.57
0.37
1.96
0.40
0.61
0.58
0.60
0.78
3.02
3.12
1.21
1.76
f1 (ppm)

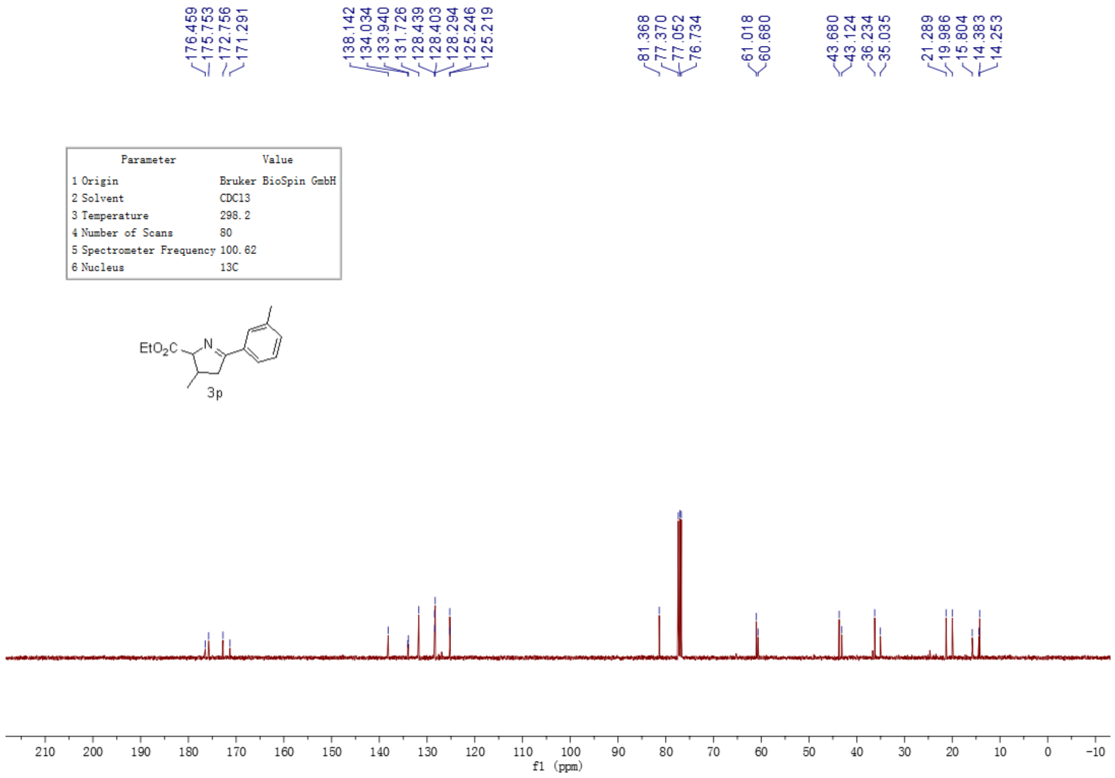
176.459
175.753
172.756
171.291
138.142
134.034
133.940
131.726
128.439
128.403
128.294
125.246
125.219
81.368
77.370
77.052
76.734
61.018
60.680
43.680
43.124
36.234
35.035
21.289
19.986
15.804
14.383
14.253
Parameter Value
1 Origin Bruker BioSpin GmbH
2 Solvent CDCl3
3 Temperature 298.2
4 Number of Scans 80
5 Spectrometer Frequency 100.62
6 Nucleus 13C
EtO2C
N
3p
f1 (ppm)

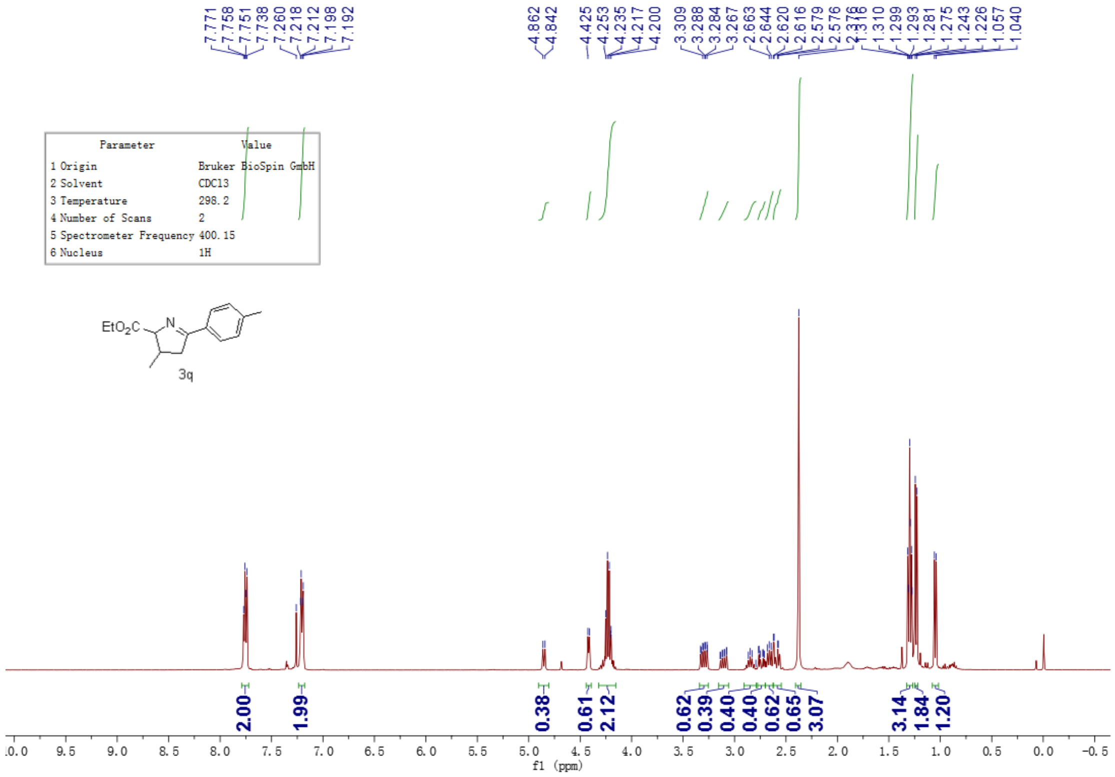
7.771
7.758
7.751
7.738
7.260
7.218
7.212
7.198
7.192
4.862
4.842
4.425
4.253
4.235
4.217
4.200
3.309
3.288
3.284
3.267
2.663
2.644
2.620
2.616
2.579
2.576
2.376
1.316
1.310
1.299
1.293
1.281
1.275
1.243
1.226
1.057
1.040
Parameter Value
1 Origin Bruker BioSpin GmbH
2 Solvent CDCl3
3 Temperature 298.2
4 Number of Scans 2
5 Spectrometer Frequency 400.15
6 Nucleus 1H
EtO2C
N
3q
2.00
1.99
0.38
0.61
2.12
0.62
0.39
0.40
0.40
0.62
0.65
3.07
3.14
1.84
1.20
f1 (ppm)

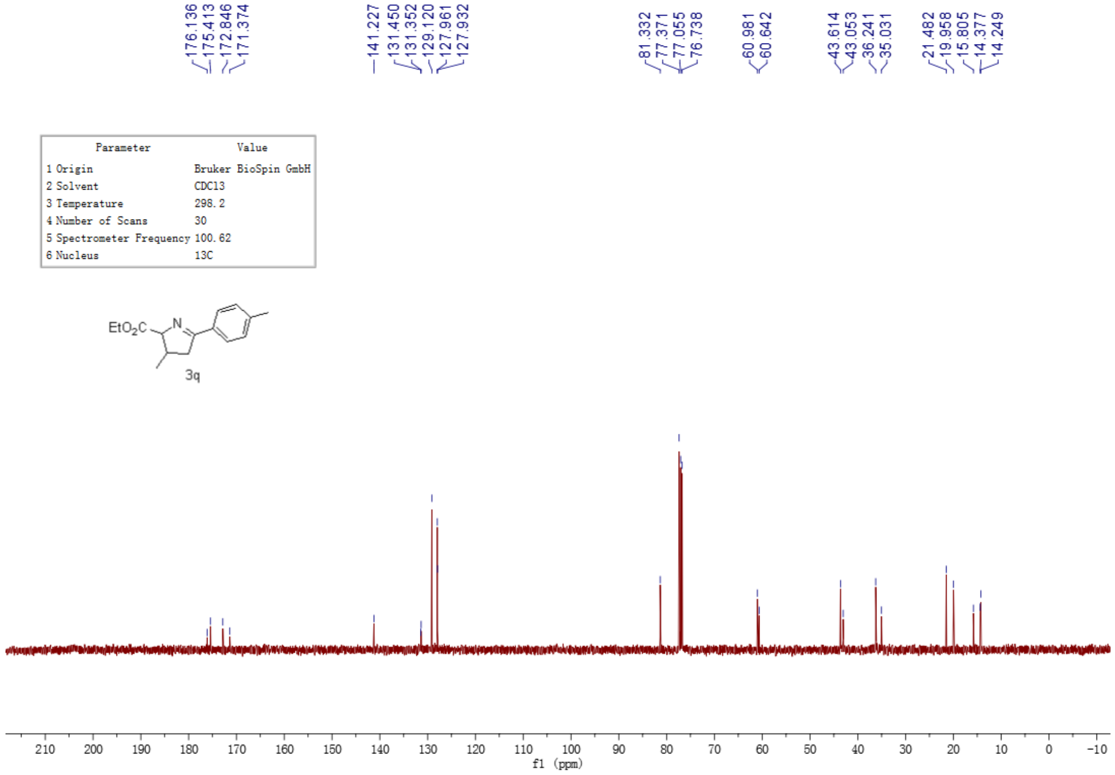
176.136
175.413
172.846
171.374
141.227
131.450
131.352
129.120
127.961
127.932
81.332
77.371
77.055
76.738
60.981
60.642
43.614
43.053
36.241
35.031
21.482
19.958
15.805
14.377
14.249
Parameter Value
1 Origin Bruker BioSpin GmbH
2 Solvent CDCl3
3 Temperature 298.2
4 Number of Scans 30
5 Spectrometer Frequency 100.62
6 Nucleus 13C
EtO2C
N
3q
f1 (ppm)

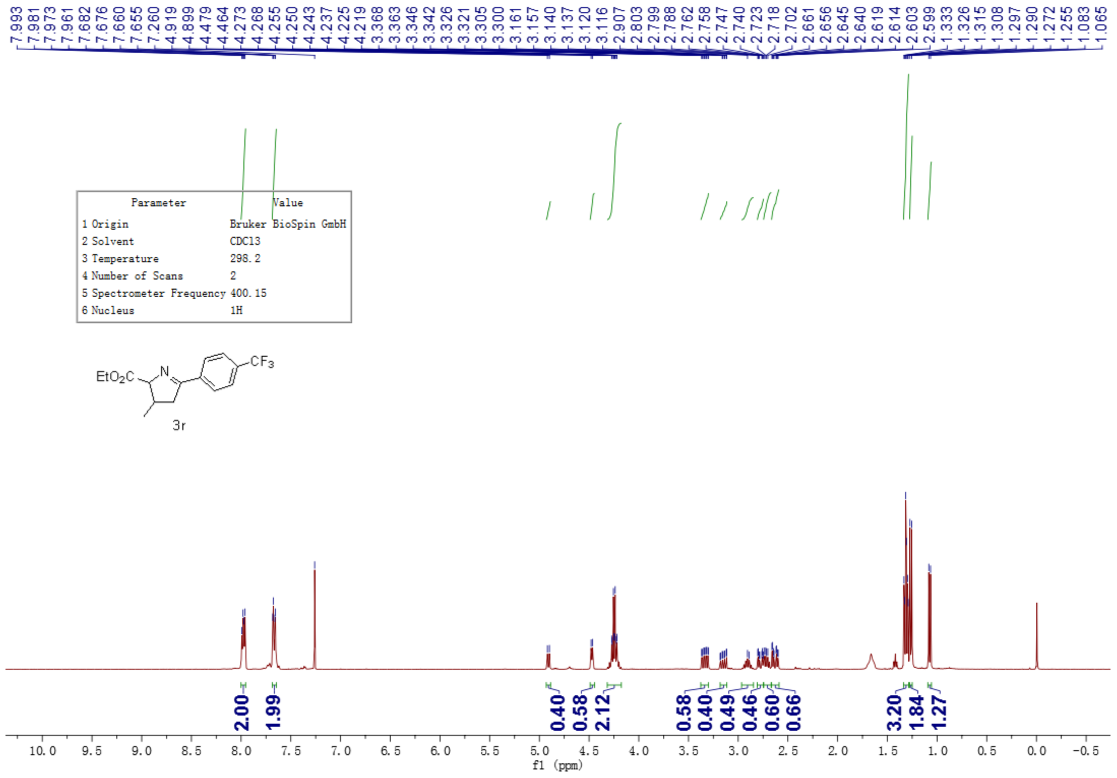
Parameter Value
1 Origin Bruker BioSpin GmbH
2 Solvent CDCl3
3 Temperature 298.2
4 Number of Scans 2
5 Spectrometer Frequency 400.15
6 Nucleus 1H
EtO2C
N
CF3
3r
f1 (ppm)

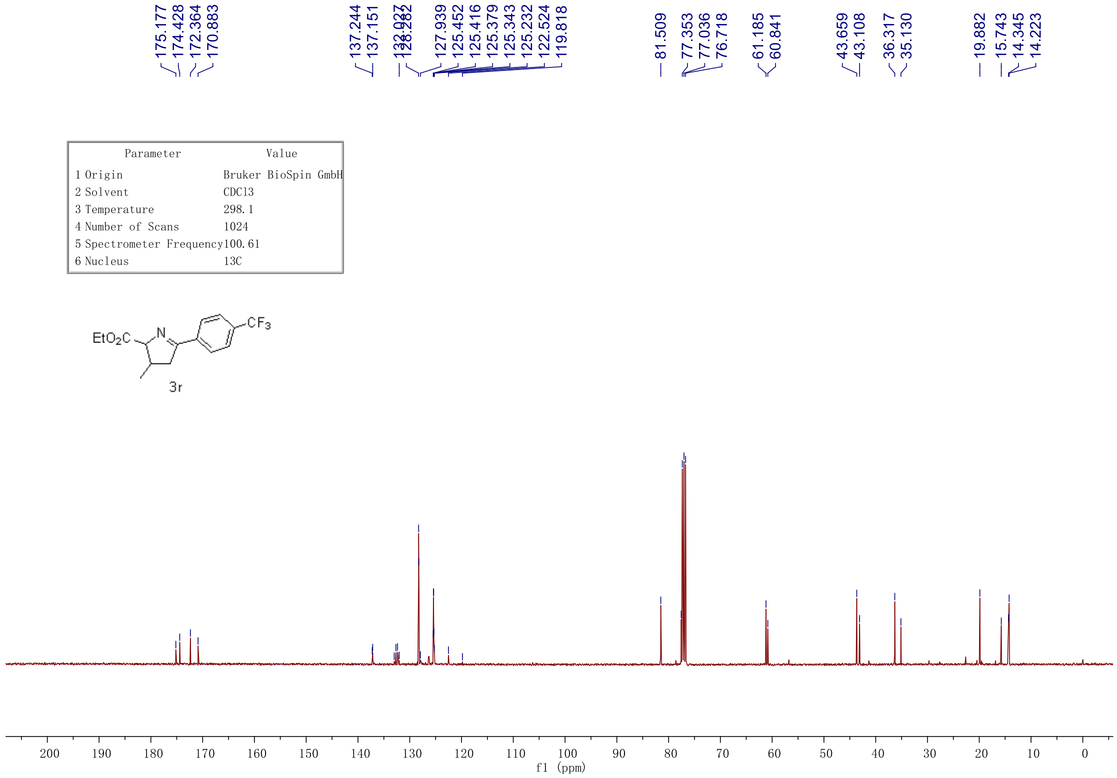
Parameter Value
1 Origin Bruker BioSpin GmbH
2 Solvent CDCl3
3 Temperature 298.1
4 Number of Scans 1024
5 Spectrometer Frequency 100.61
6 Nucleus 13C
EtO2C
N
CF3
3r
f1 (ppm)

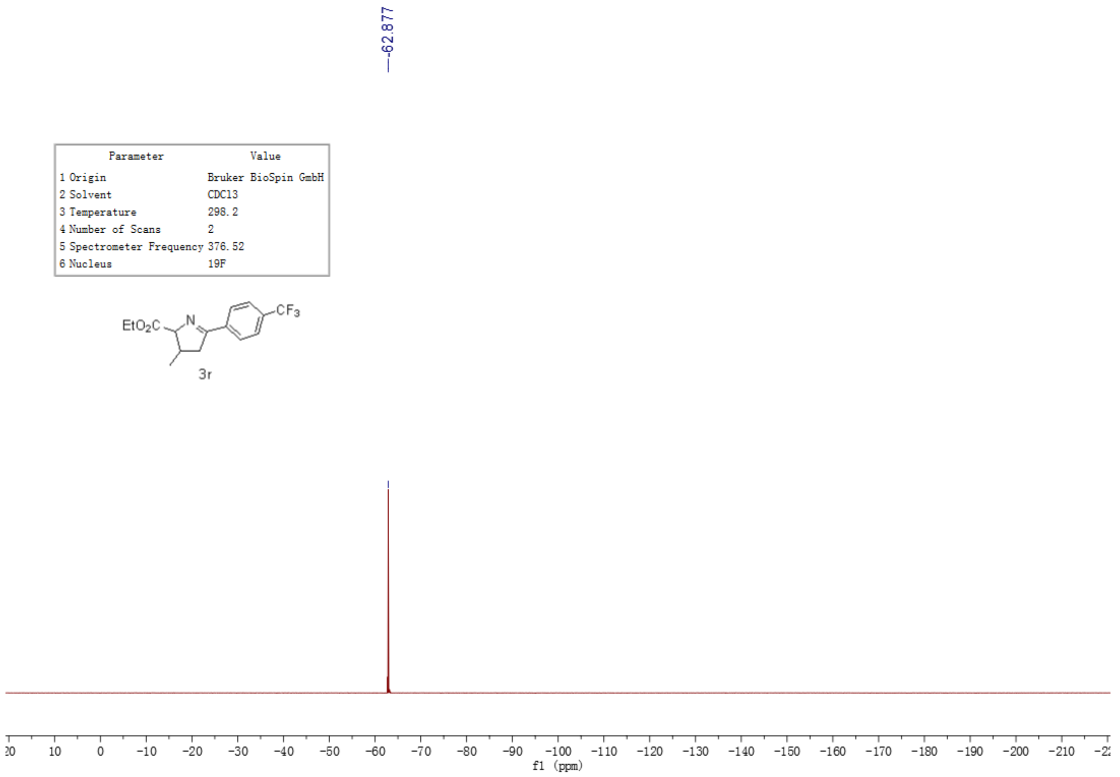

Parameter	Value
1 Origin	Bruker BioSpin GmbH
2 Solvent	CDCl3
3 Temperature	298.2
4 Number of Scans	2
5 Spectrometer Frequency	376.52
6 Nucleus	19F

3r

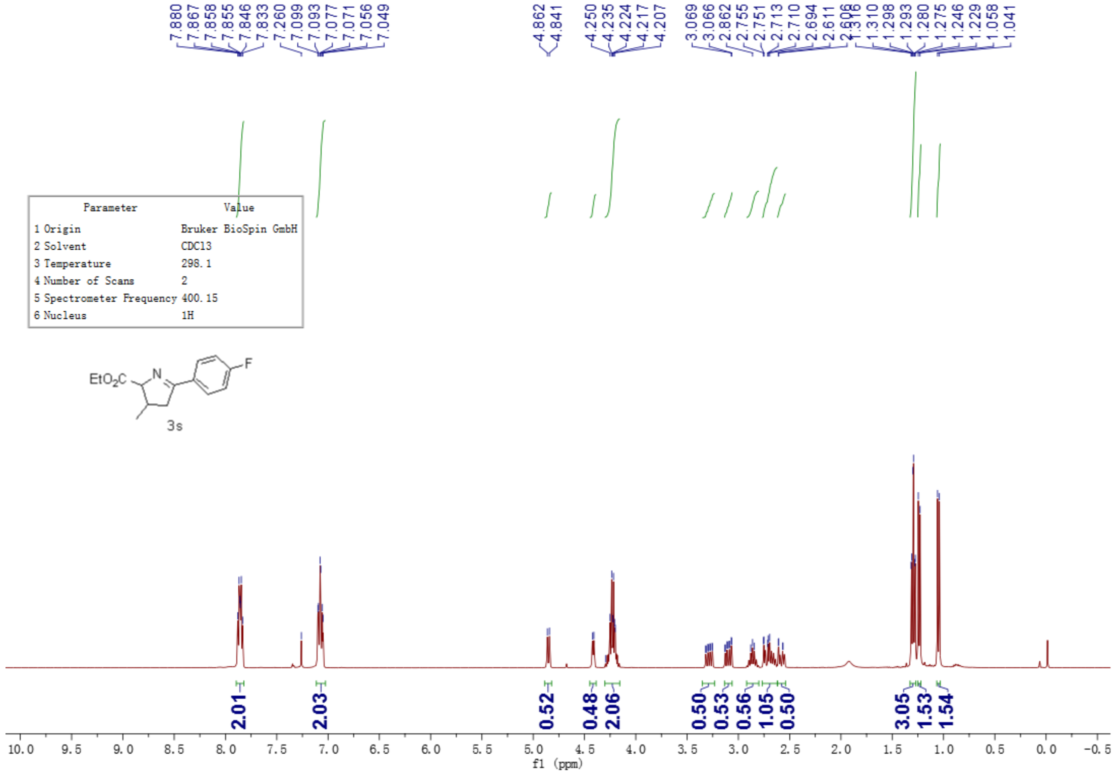

Parameter	Value
1 Origin	Bruker BioSpin GmbH
2 Solvent	CDCl3
3 Temperature	298.1
4 Number of Scans	2
5 Spectrometer Frequency	400.15
6 Nucleus	1H

3s

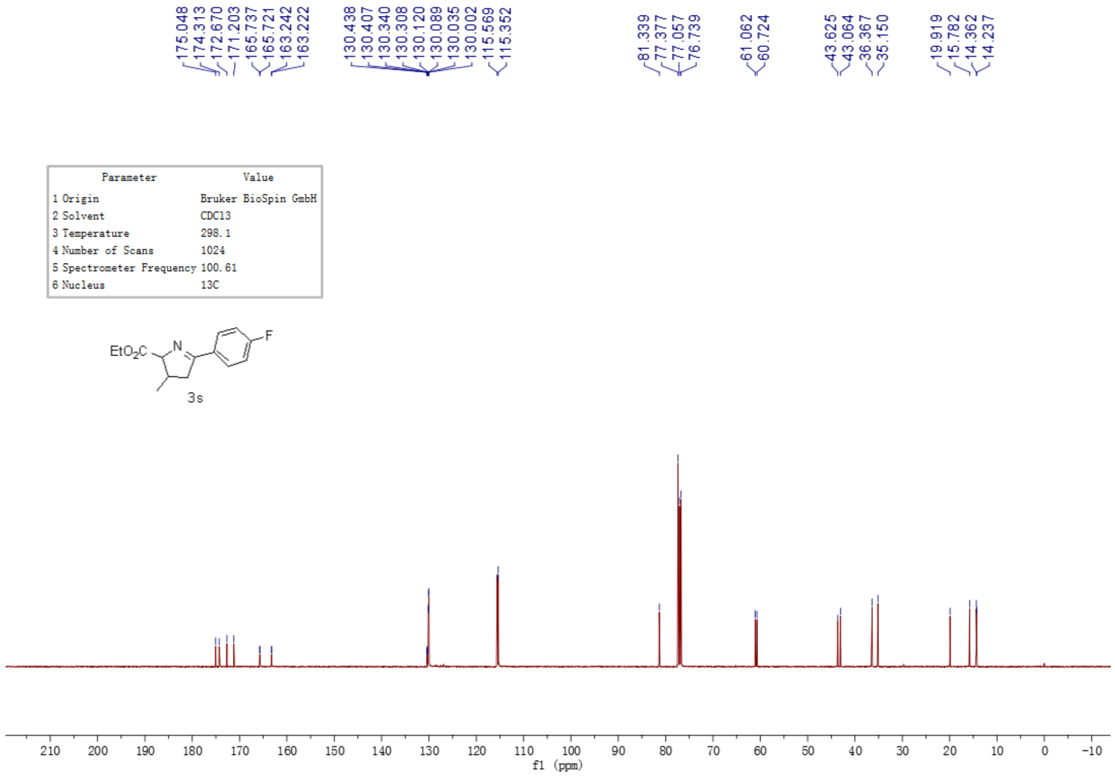
175.048
174.313
172.670
171.203
165.737
165.721
163.242
163.222
130.438
130.407
130.340
130.308
130.120
130.089
130.035
130.002
115.569
115.352
81.339
77.377
77.057
76.739
61.062
60.724
43.625
43.064
36.367
35.150
19.919
15.782
14.362
14.237
Parameter Value
1 Origin Bruker BioSpin GmbH
2 Solvent CDCl3
3 Temperature 298.1
4 Number of Scans 1024
5 Spectrometer Frequency 100.61
6 Nucleus 13C
EtO2C
N
F
3s
f1 (ppm)

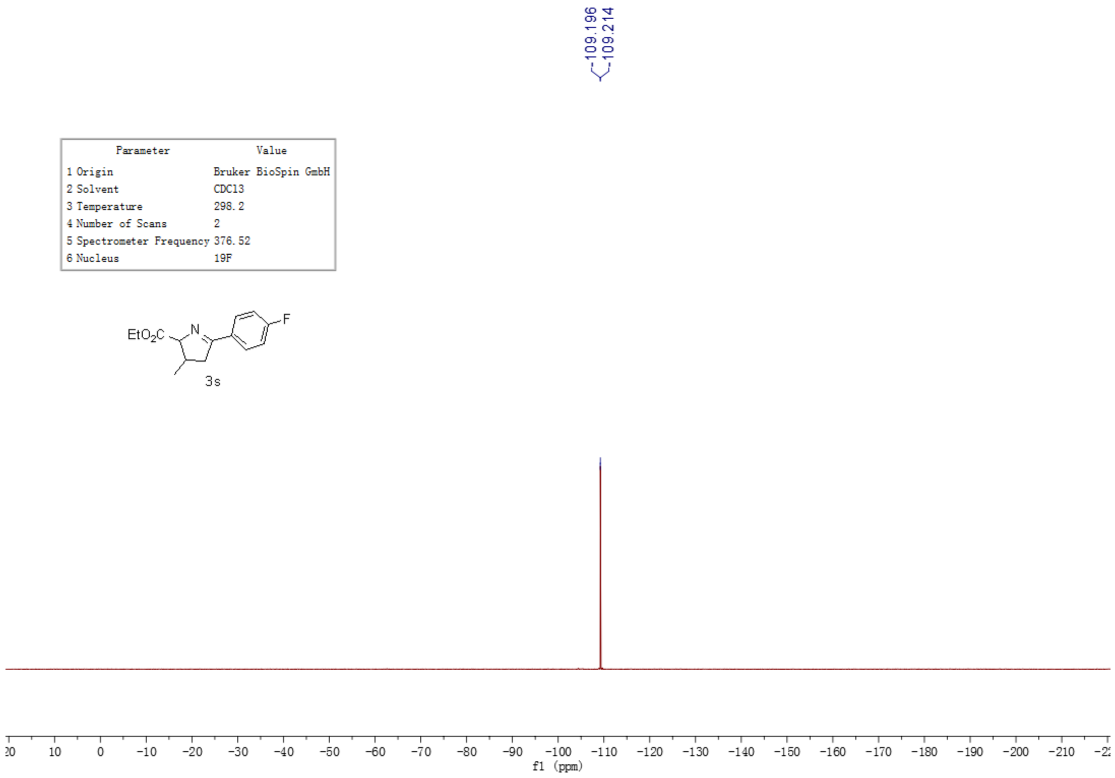
-109.196
-109.214
Parameter Value
1 Origin Bruker BioSpin GmbH
2 Solvent CDCl3
3 Temperature 298.2
4 Number of Scans 2
5 Spectrometer Frequency 376.52
6 Nucleus 19F
EtO2C
N
F
3s
f1 (ppm)

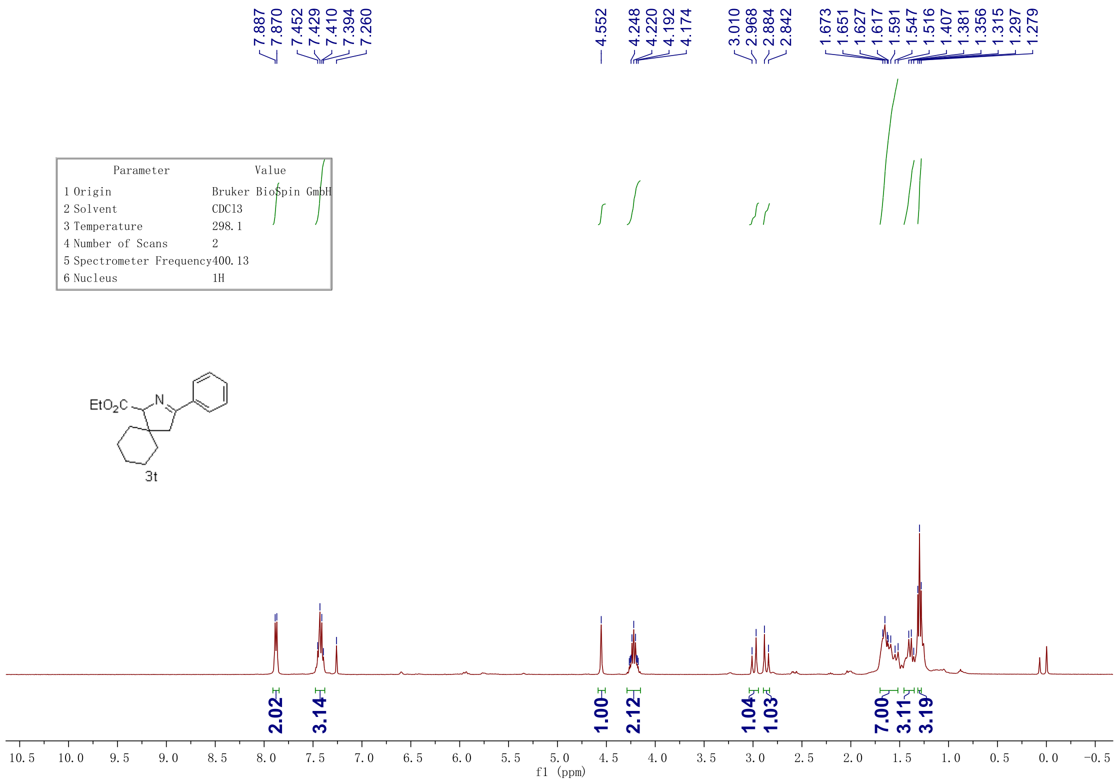
7.887
7.870
7.452
7.429
7.410
7.394
7.260
4.552
4.248
4.220
4.192
4.174
3.010
2.968
2.884
2.842
1.673
1.651
1.627
1.617
1.591
1.547
1.516
1.407
1.381
1.356
1.315
1.297
1.279
Parameter Value
1 Origin Bruker BioSpin GmbH
2 Solvent CDCl3
3 Temperature 298.1
4 Number of Scans 2
5 Spectrometer Frequency 400.13
6 Nucleus 1H
EtO2C
N
3t
2.02
3.14
1.00
2.12
1.04
1.03
7.00
3.11
3.19
f1 (ppm)

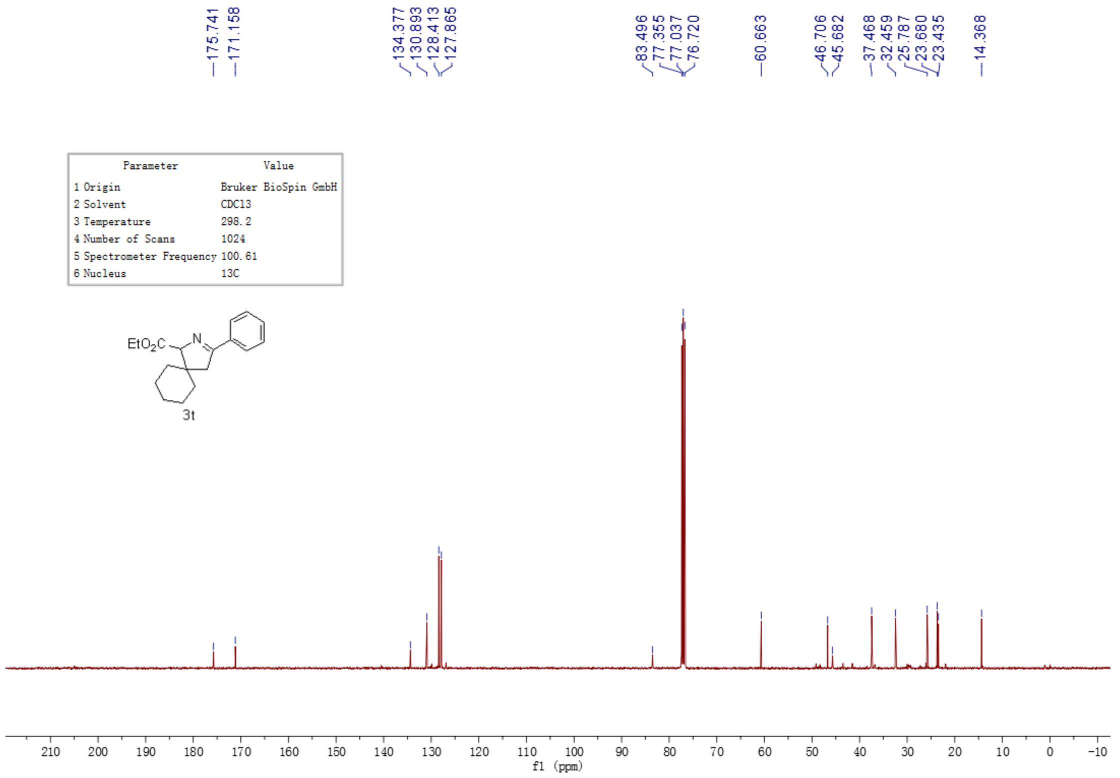
175.741
171.158
134.377
130.893
128.413
127.865
83.496
77.355
77.037
76.720
60.663
46.706
45.682
37.468
32.459
25.787
23.680
23.435
14.368
Parameter Value
1 Origin Bruker BioSpin GmbH
2 Solvent CDCl3
3 Temperature 298.2
4 Number of Scans 1024
5 Spectrometer Frequency 100.61
6 Nucleus 13C
EtO2C
N
3t
f1 (ppm)

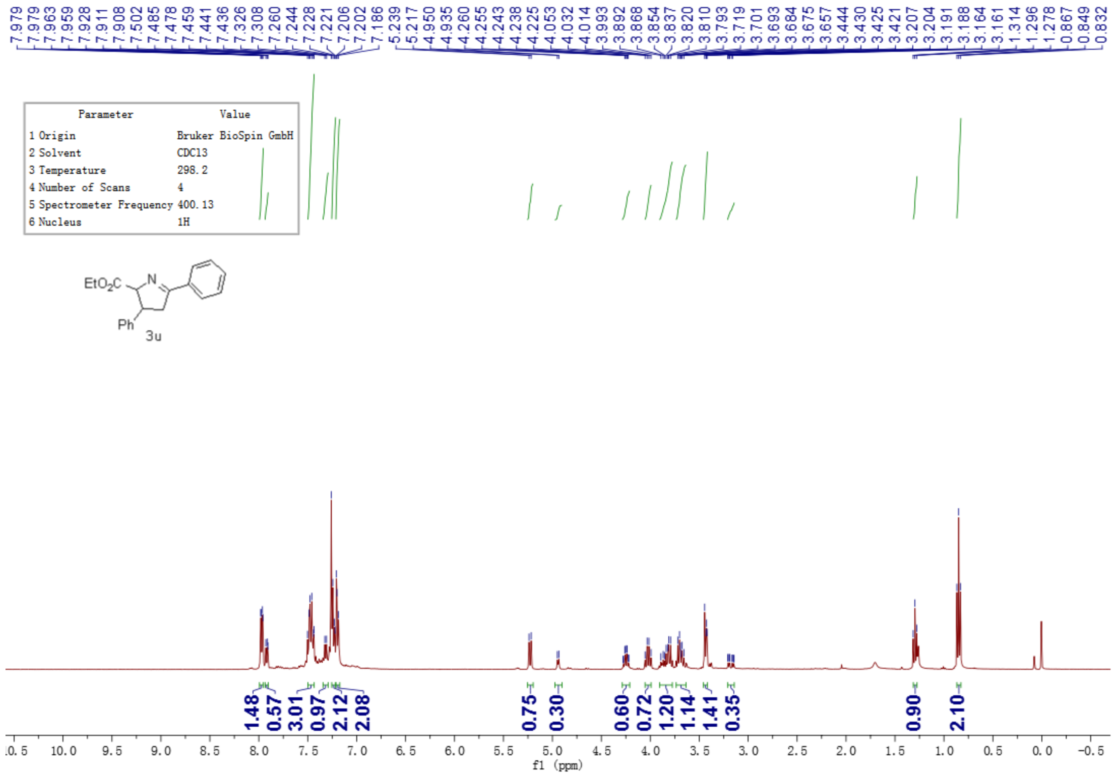

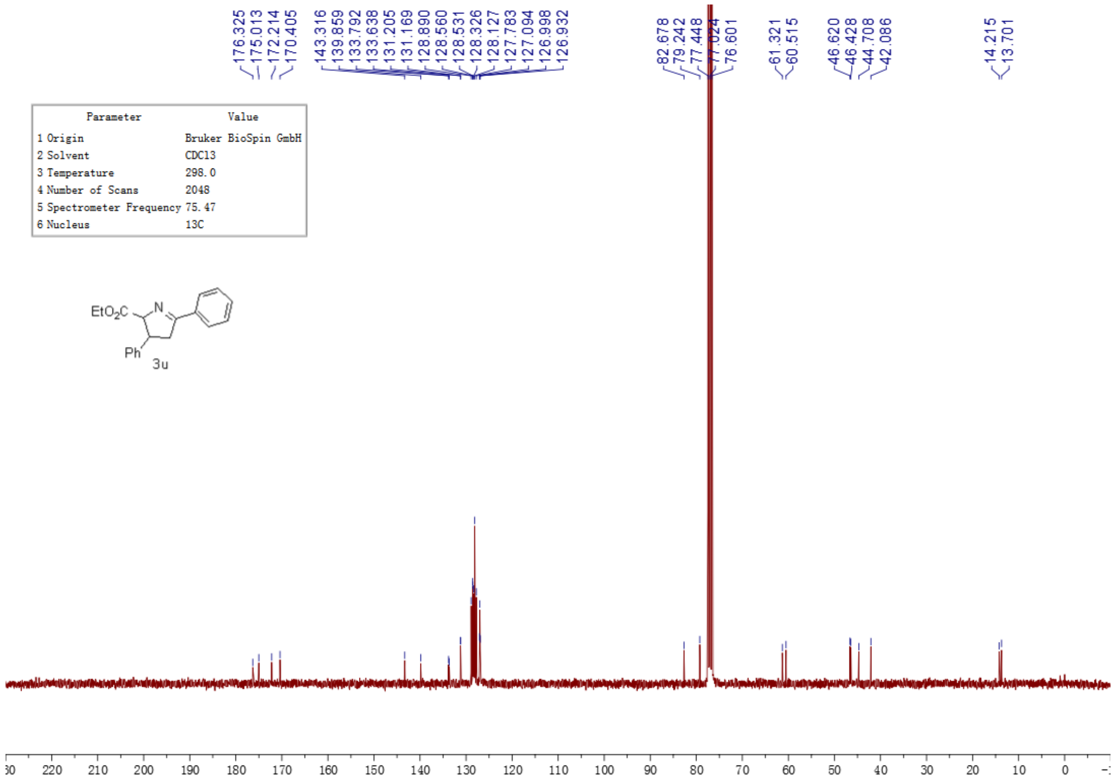

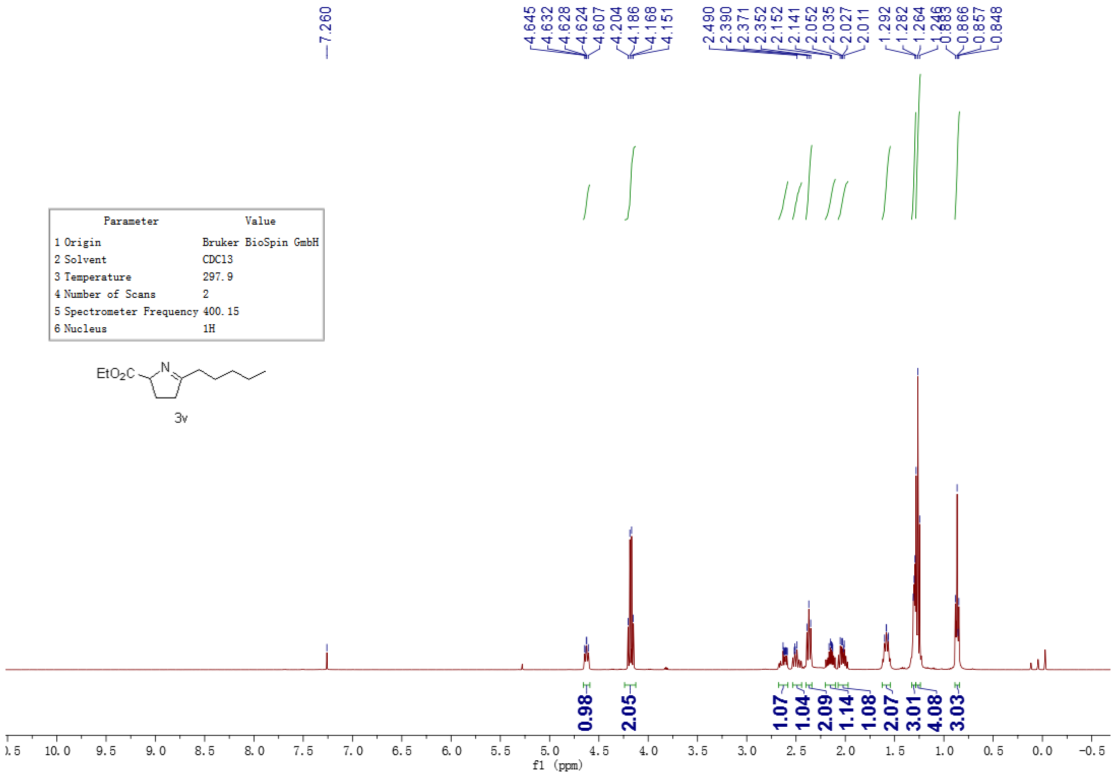

7.260
4.645 4.632 4.628 4.624 4.607 4.204 4.186 4.168 4.151
2.490 2.390 2.371 2.352 2.152 2.141 2.052 2.035 2.027 2.011
1.292 1.282 1.264 1.246 0.883 0.866 0.857 0.848
Parameter Value
1 Origin Bruker BioSpin GmbH
2 Solvent CDCl3
3 Temperature 297.9
4 Number of Scans 2
5 Spectrometer Frequency 400.15
6 Nucleus 1H
EtO2C
N
3v
0.98 2.05 1.07 1.04 2.09 1.14 1.08 2.07 3.01 4.08 3.03
f1 (ppm)

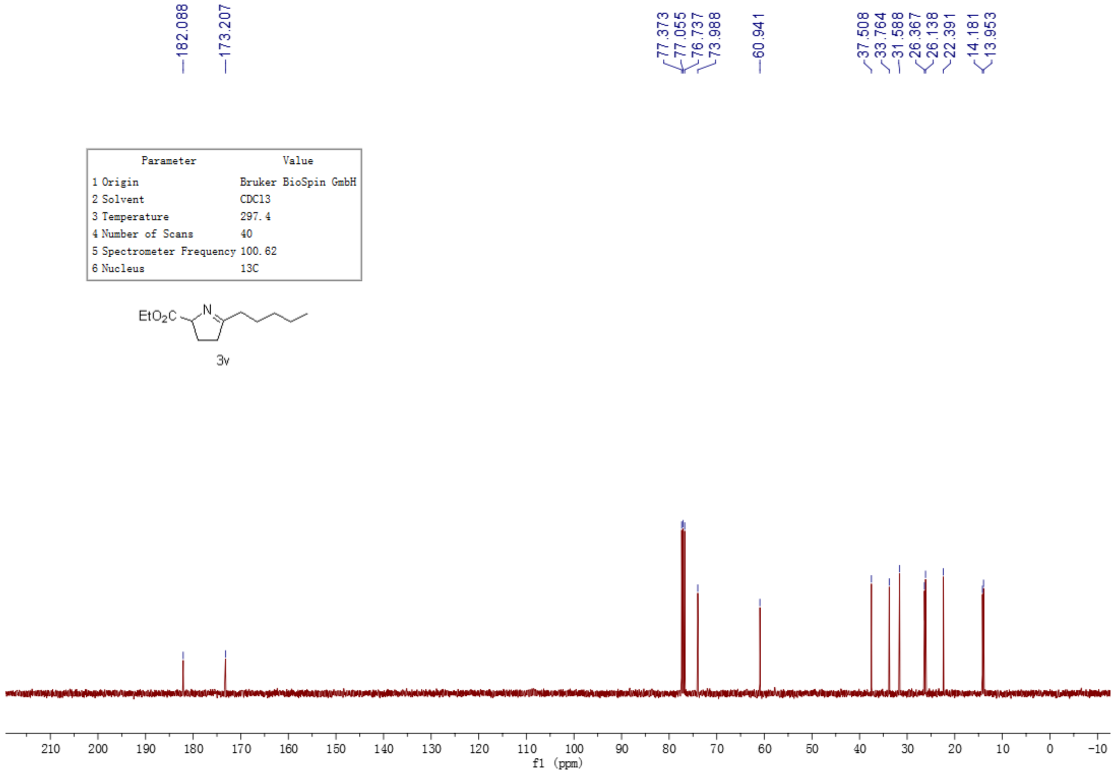

182.088
173.207
77.373 77.055 76.737 73.988
60.941
37.508 33.764 31.588 26.367 26.138 22.391 14.181 13.953
Parameter Value
1 Origin Bruker BioSpin GmbH
2 Solvent CDCl3
3 Temperature 297.4
4 Number of Scans 40
5 Spectrometer Frequency 100.62
6 Nucleus 13C
EtO2C
N
3v
f1 (ppm)

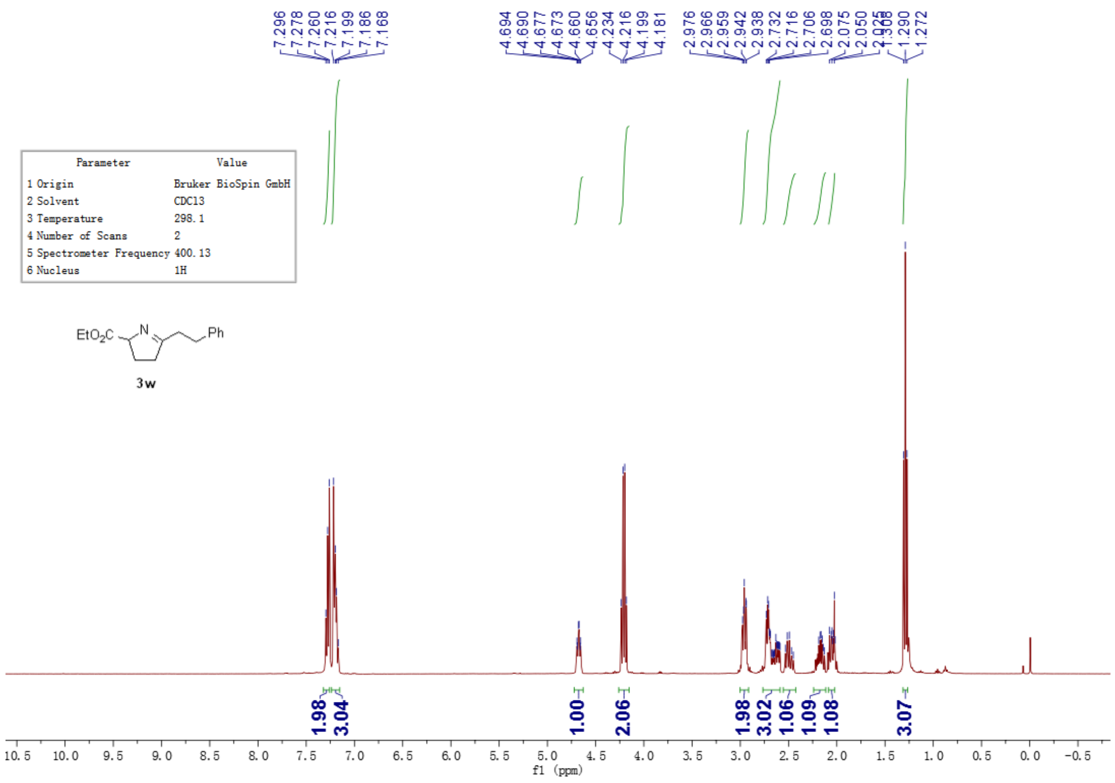
7.296
7.278
7.260
7.216
7.199
7.186
7.168
4.694
4.690
4.677
4.673
4.660
4.656
4.234
4.216
4.199
4.181
2.976
2.966
2.959
2.942
2.938
2.732
2.716
2.706
2.698
2.075
2.050
1.290
1.272
Parameter Value
1 Origin Bruker BioSpin GmbH
2 Solvent CDCl3
3 Temperature 298.1
4 Number of Scans 2
5 Spectrometer Frequency 400.13
6 Nucleus 1H
EtO2C
N
Ph
3w
1.98
3.04
1.00
2.06
1.98
3.02
1.06
1.09
1.08
3.07
f1 (ppm)

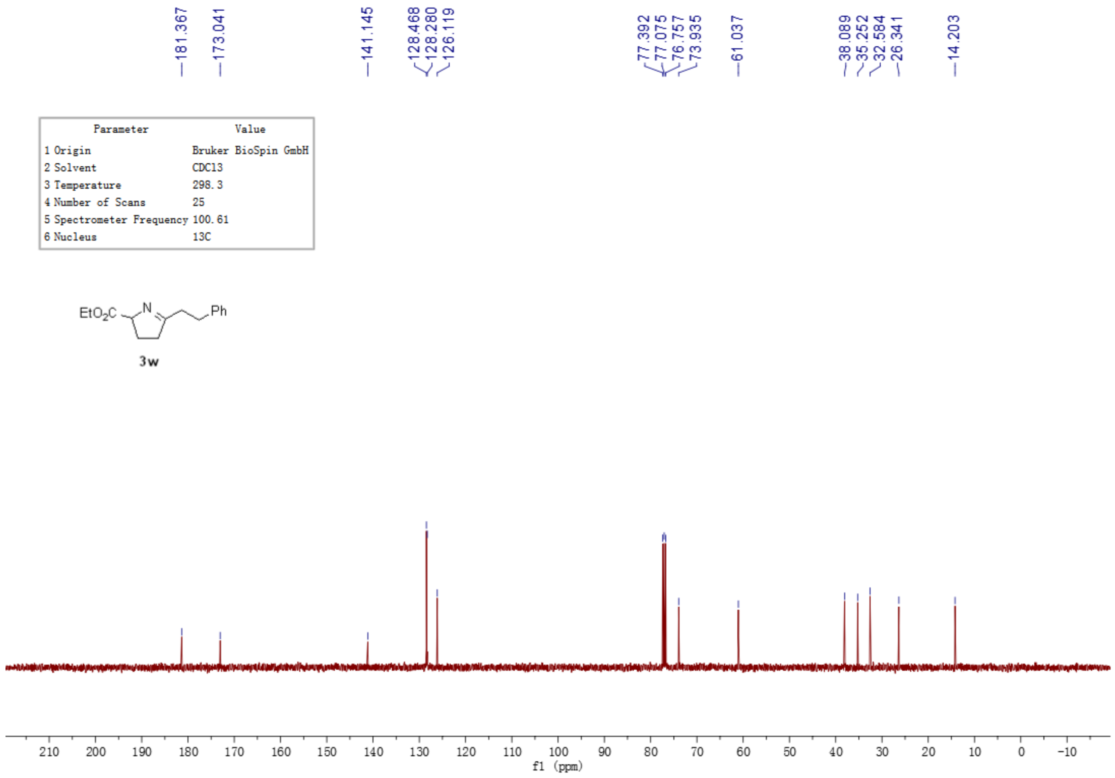
181.367
173.041
141.145
128.468
128.280
126.119
77.392
77.075
76.757
73.935
61.037
38.089
35.252
32.584
26.341
14.203
Parameter Value
1 Origin Bruker BioSpin GmbH
2 Solvent CDCl3
3 Temperature 298.3
4 Number of Scans 25
5 Spectrometer Frequency 100.61
6 Nucleus 13C
EtO2C
N
Ph
3w
f1 (ppm)

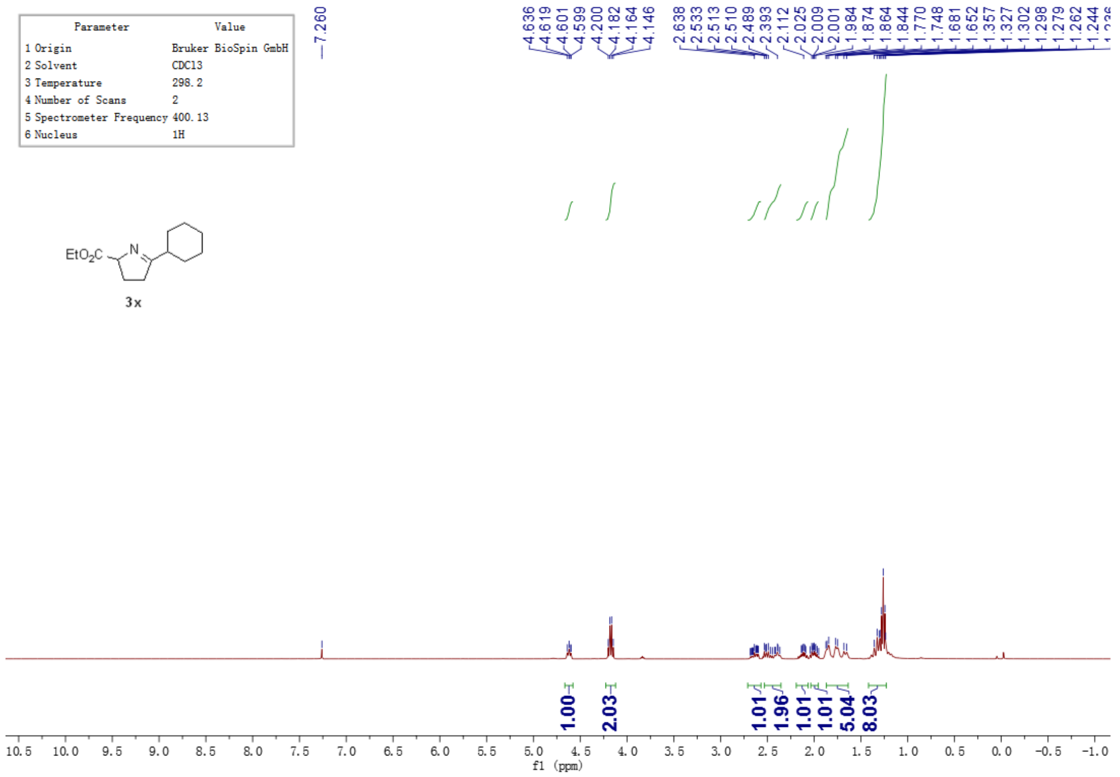
Parameter Value
1 Origin Bruker BioSpin GmbH
2 Solvent CDCl3
3 Temperature 298.2
4 Number of Scans 2
5 Spectrometer Frequency 400.13
6 Nucleus 1H
7.260
4.636 4.619 4.601 4.599 4.200 4.182 4.164 4.146
2.638 2.533 2.513 2.510 2.489 2.393 2.112 2.025 2.009 2.001 1.984 1.874 1.864 1.844 1.770 1.748 1.681 1.652 1.357 1.327 1.302 1.298 1.279 1.262 1.244
EtO2C
N
3x
1.00 2.03 1.01 1.96 1.01 1.01 5.04 8.03
f1 (ppm)

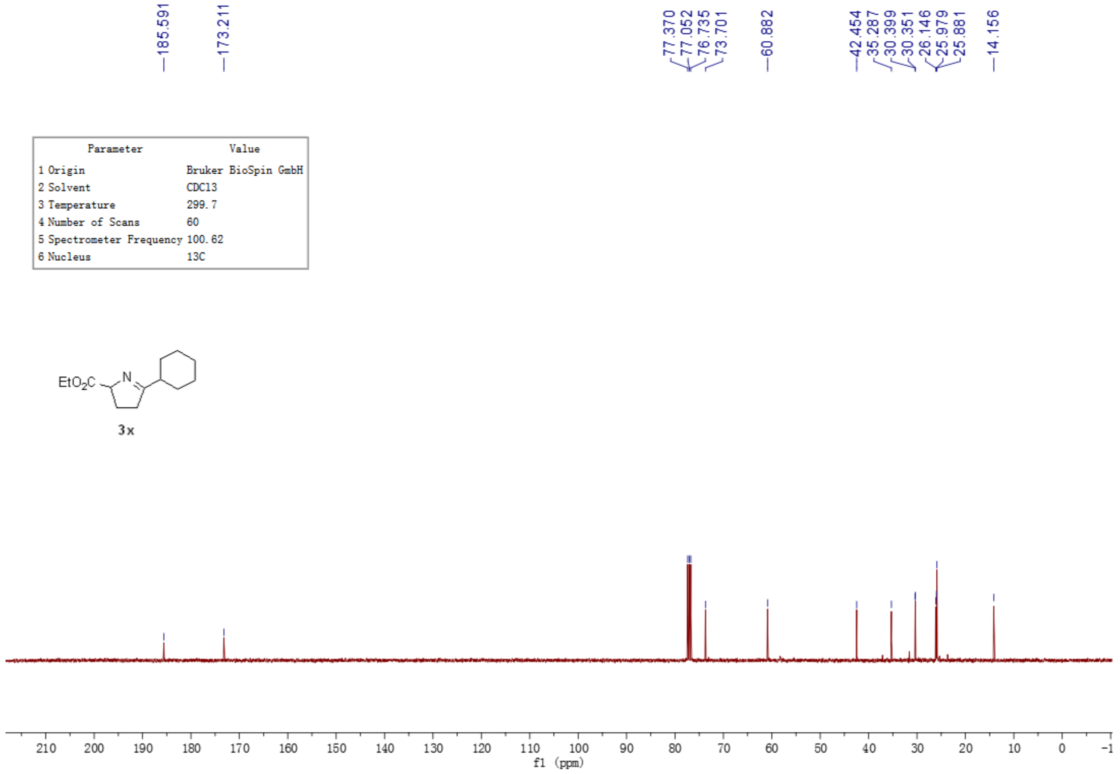
185.591
173.211
77.370 77.052 76.735 73.701
60.882
42.454 35.287 30.399 30.351 26.146 25.979 25.881
14.156
Parameter Value
1 Origin Bruker BioSpin GmbH
2 Solvent CDCl3
3 Temperature 299.7
4 Number of Scans 60
5 Spectrometer Frequency 100.62
6 Nucleus 13C
EtO2C
N
3x
f1 (ppm)

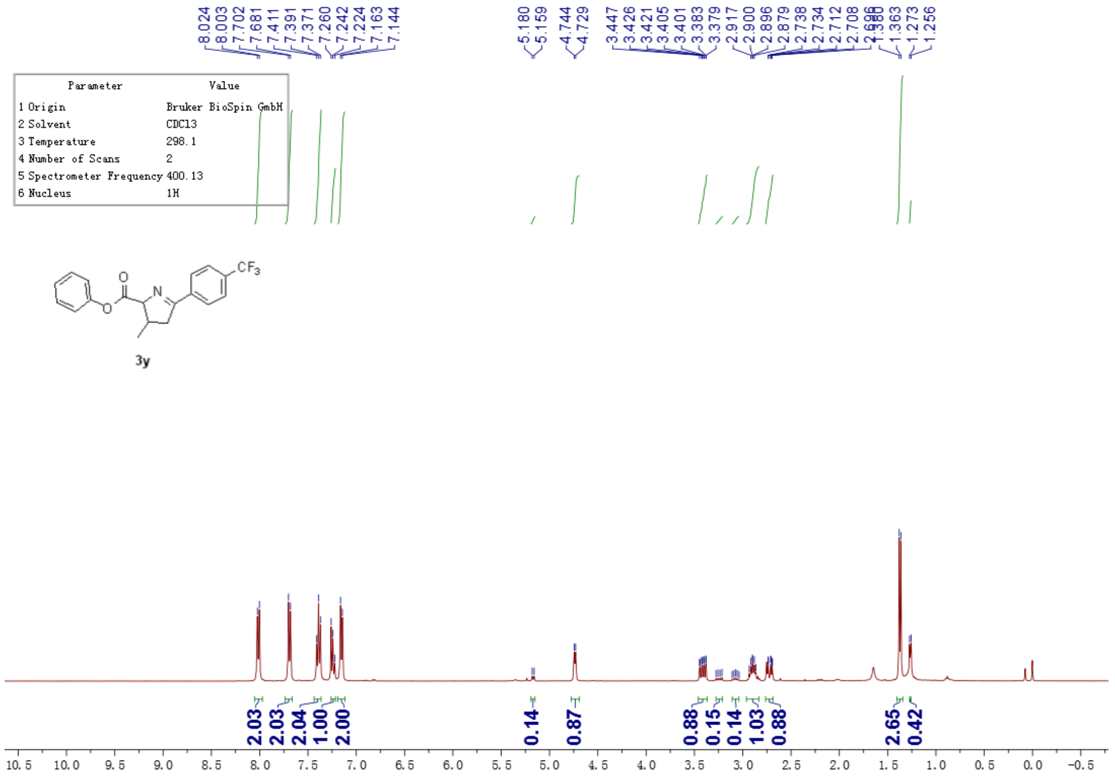
8.024
8.003
7.702
7.681
7.411
7.391
7.371
7.260
7.242
7.224
7.163
7.144
5.180
5.159
4.744
4.729
3.447
3.426
3.421
3.405
3.401
3.383
3.379
2.917
2.900
2.896
2.879
2.738
2.734
2.712
2.708
1.380
1.363
1.273
1.256
Parameter
Value
1 Origin
Bruker BioSpin GmbH
2 Solvent
CDCl3
3 Temperature
298.1
4 Number of Scans
2
5 Spectrometer Frequency
400.13
6 Nucleus
1H
CF3
O
N
O
3y
2.03
2.03
2.04
1.00
2.00
0.14
0.87
0.88
0.15
0.14
1.03
0.88
2.65
0.42
10.5
10.0
9.5
9.0
8.5
8.0
7.5
7.0
6.5
6.0
5.5
5.0
4.5
4.0
3.5
3.0
2.5
2.0
1.5
1.0
0.5
0.0
-0.5

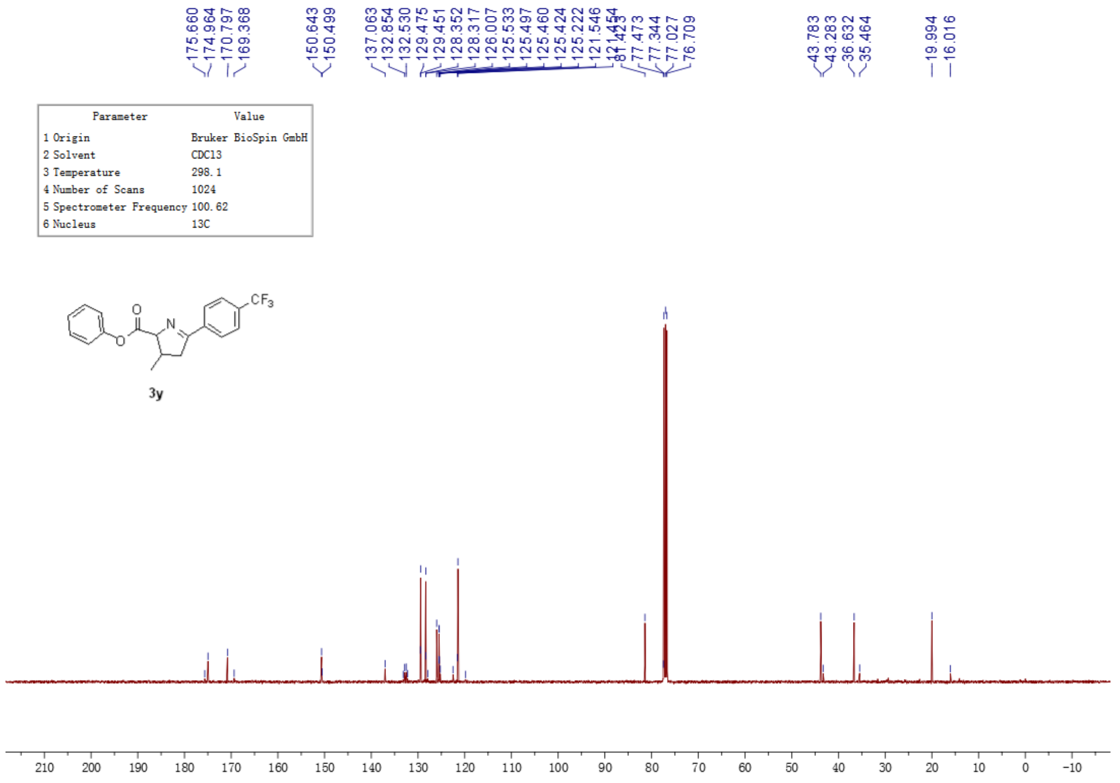
175.660
174.964
170.797
169.368
150.643
150.499
137.063
132.854
132.530
129.475
129.451
128.352
128.317
126.007
125.533
125.497
125.460
125.424
125.222
121.546
121.454
81.423
77.473
77.344
77.027
76.709
43.783
43.283
36.632
35.464
19.994
16.016
Parameter
Value
1 Origin
Bruker BioSpin GmbH
2 Solvent
CDCl3
3 Temperature
298.1
4 Number of Scans
1024
5 Spectrometer Frequency
100.62
6 Nucleus
13C
CF3
O
N
O
3y
210
200
190
180
170
160
150
140
130
120
110
100
90
80
70
60
50
40
30
20
10
0
-10

Parameter	Value
1 Origin	Bruker BioSpin GmbH
2 Solvent	CDCl3
3 Temperature	298.2
4 Number of Scans	2
5 Spectrometer Frequency	376.52
6 Nucleus	19F

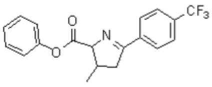

3y

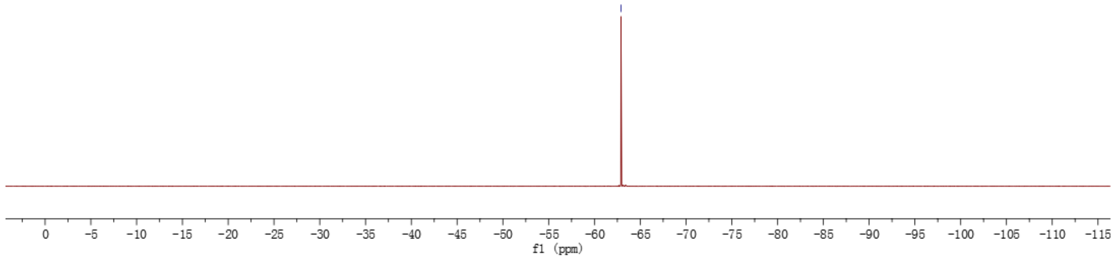

Parameter	Value
1 Origin	Bruker BioSpin GmbH
2 Solvent	CDCl3
3 Temperature	298.1
4 Number of Scans	2
5 Spectrometer Frequency	400.13
6 Nucleus	1H

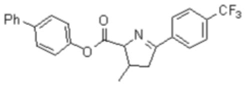

3z

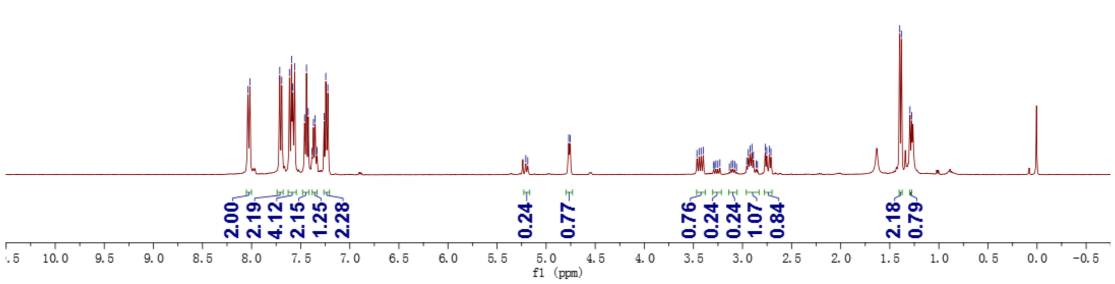

Parameter	Value
1 Origin	Bruker BioSpin GmbH
2 Solvent	CDCl3
3 Temperature	298.2
4 Number of Scans	1024
5 Spectrometer Frequency	100.62
6 Nucleus	13C

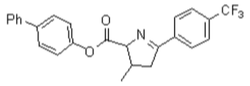

3z

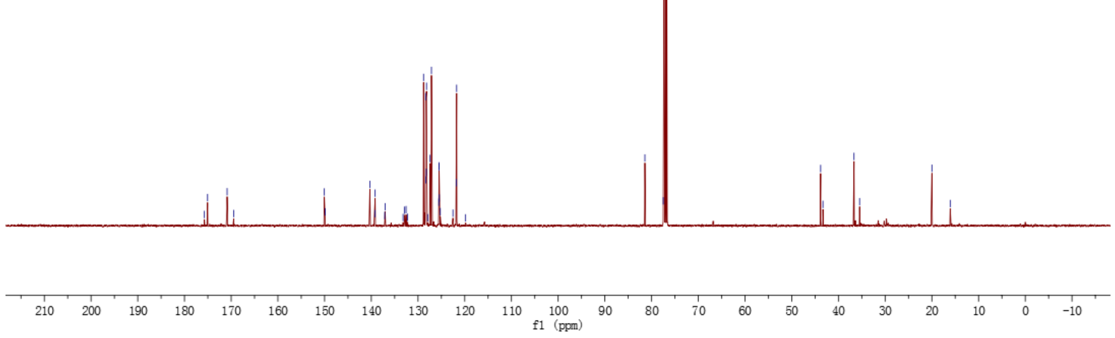

Parameter	Value
1 Origin	Bruker BioSpin GmbH
2 Solvent	CDCl3
3 Temperature	298.2
4 Number of Scans	2
5 Spectrometer Frequency	376.52
6 Nucleus	19F

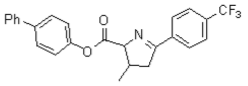

3z

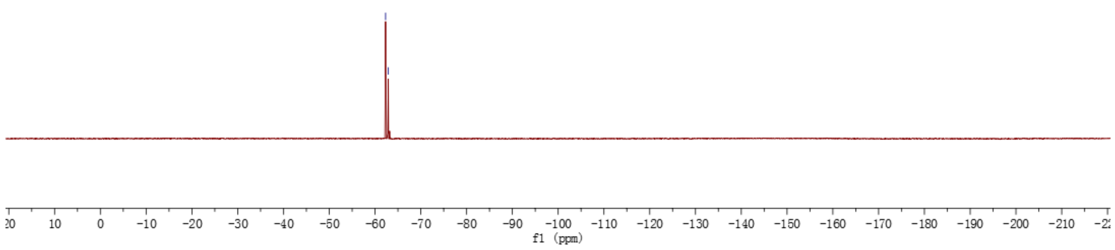

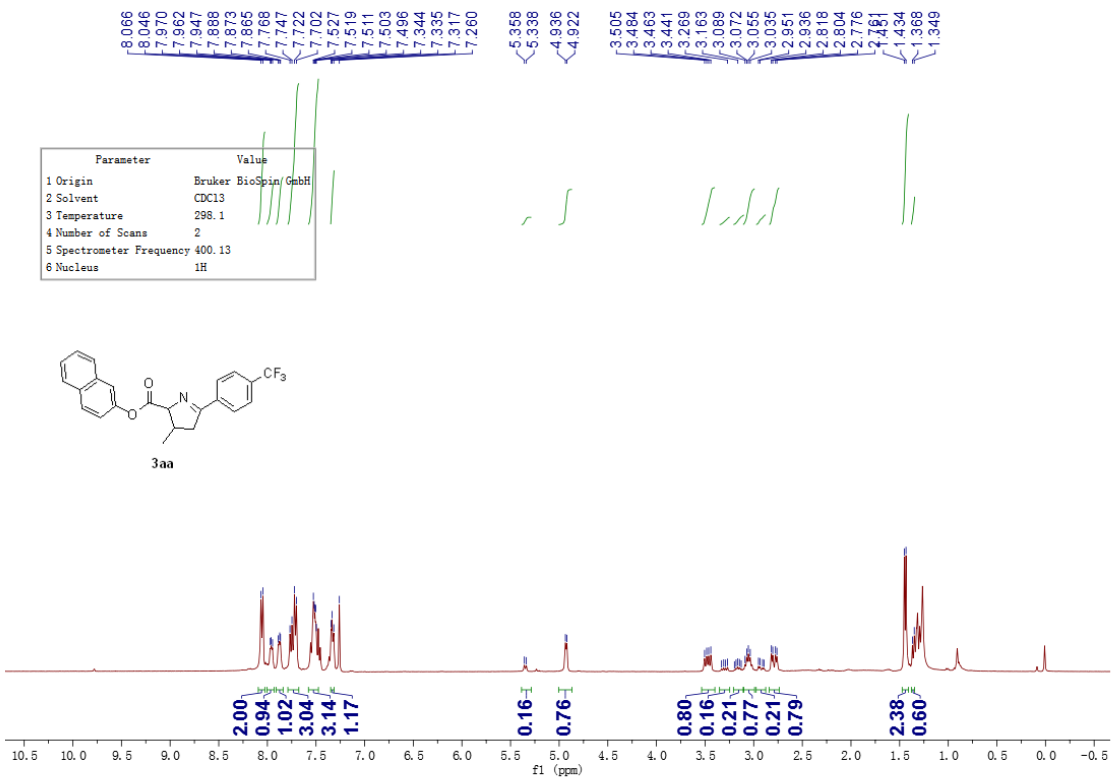
8.066
8.046
7.970
7.962
7.947
7.888
7.873
7.865
7.768
7.747
7.722
7.702
7.527
7.519
7.511
7.503
7.496
7.344
7.335
7.317
7.260
5.358
5.338
4.936
4.922
3.505
3.484
3.463
3.441
3.269
3.163
3.089
3.072
3.055
3.035
2.951
2.936
2.818
2.804
2.776
2.761
1.451
1.434
1.368
1.349
Parameter Value
1 Origin Bruker BioSpin GmbH
2 Solvent CDC13
3 Temperature 298.1
4 Number of Scans 2
5 Spectrometer Frequency 400.13
6 Nucleus 1H
CF3
O
N
O
3aa
2.00
0.94
1.02
3.04
3.14
1.17
0.16
0.76
0.80
0.16
0.21
0.77
0.21
0.79
2.38
0.60
10.5 10.0 9.5 9.0 8.5 8.0 7.5 7.0 6.5 6.0 5.5 5.0 4.5 4.0 3.5 3.0 2.5 2.0 1.5 1.0 0.5 0.0 -0.5
f1 (ppm)

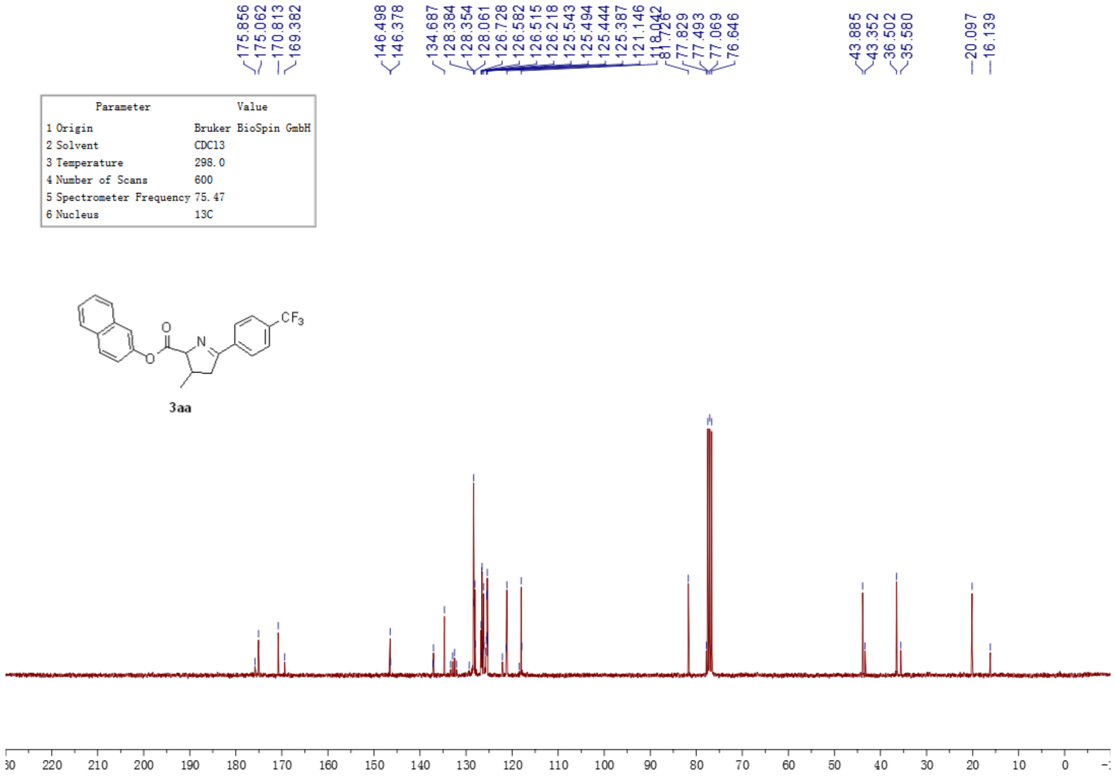
175.856
175.062
170.813
169.382
146.498
146.378
134.687
128.384
128.354
128.061
126.728
126.582
126.515
126.218
125.543
125.494
125.444
125.387
121.146
118.042
81.726
77.829
77.493
77.069
76.646
43.885
43.352
36.502
35.580
20.097
16.139
Parameter Value
1 Origin Bruker BioSpin GmbH
2 Solvent CDC13
3 Temperature 298.0
4 Number of Scans 600
5 Spectrometer Frequency 75.47
6 Nucleus 13C
CF3
O
N
O
3aa
220 210 200 190 180 170 160 150 140 130 120 110 100 90 80 70 60 50 40 30 20 10 0

Parameter	Value
1 Origin	Bruker BioSpin GmbH
2 Solvent	CDCl3
3 Temperature	298.2
4 Number of Scans	2
5 Spectrometer Frequency	376.52
6 Nucleus	19F

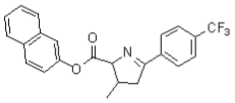

3aa

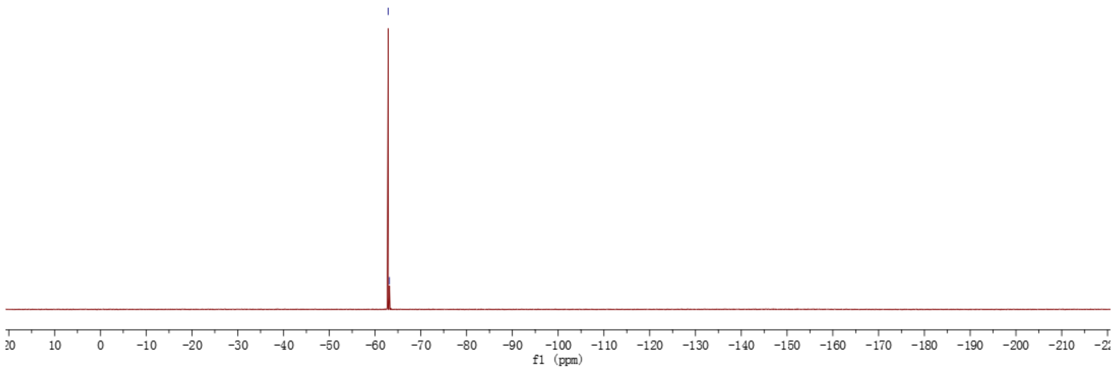

Parameter	Value
1 Origin	Bruker BioSpin GmbH
2 Solvent	CDCl3
3 Temperature	298.2
4 Number of Scans	2
5 Spectrometer Frequency	400.15
6 Nucleus	1H

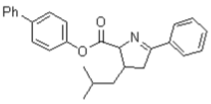

3ab

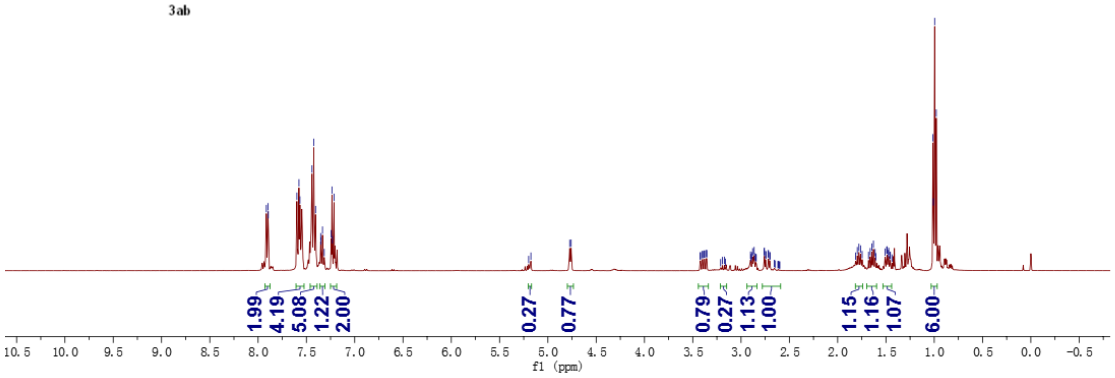

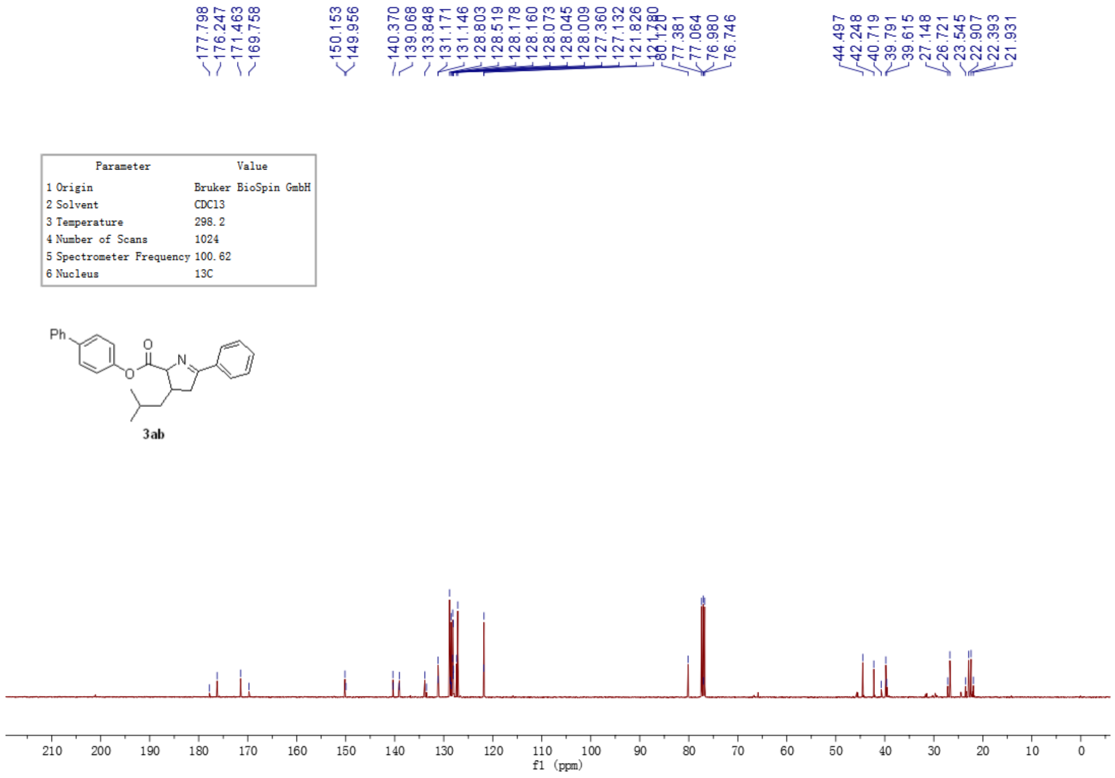

Parameter	Value
1 Origin	Bruker BioSpin GmbH
2 Solvent	CDCl3
3 Temperature	298.2
4 Number of Scans	1024
5 Spectrometer Frequency	100.62
6 Nucleus	13C

3ab

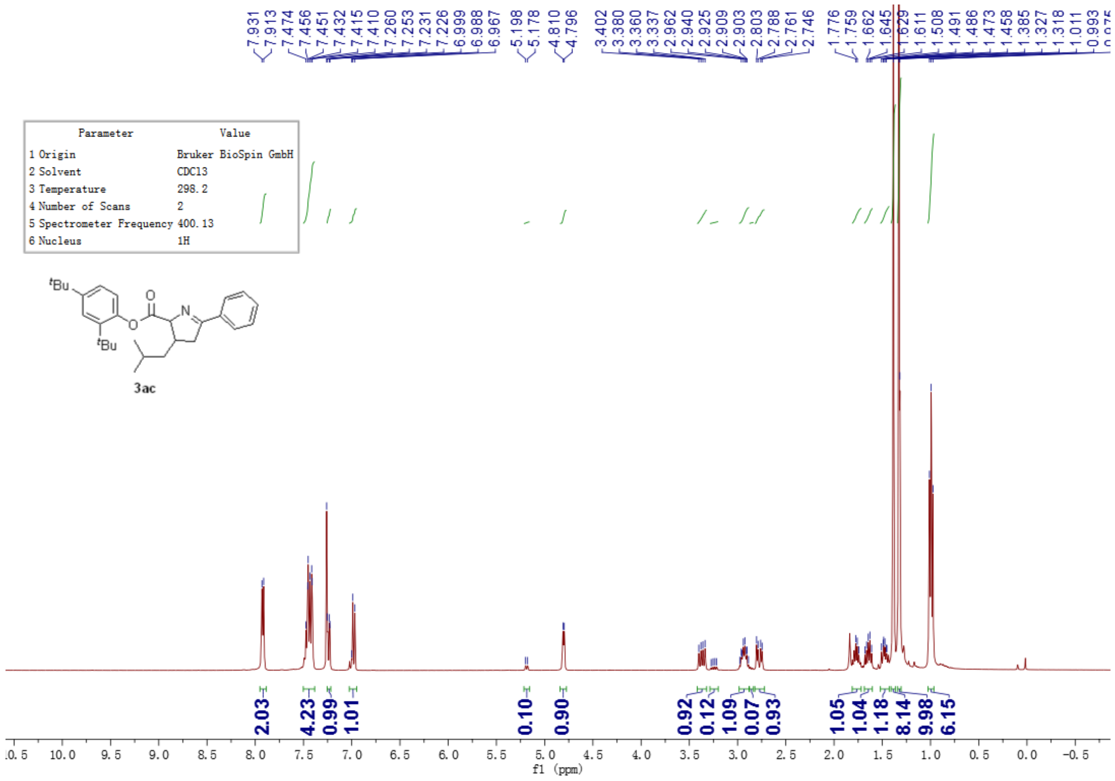

Parameter	Value
1 Origin	Bruker BioSpin GmbH
2 Solvent	CDCl3
3 Temperature	298.2
4 Number of Scans	2
5 Spectrometer Frequency	400.13
6 Nucleus	1H

3ac

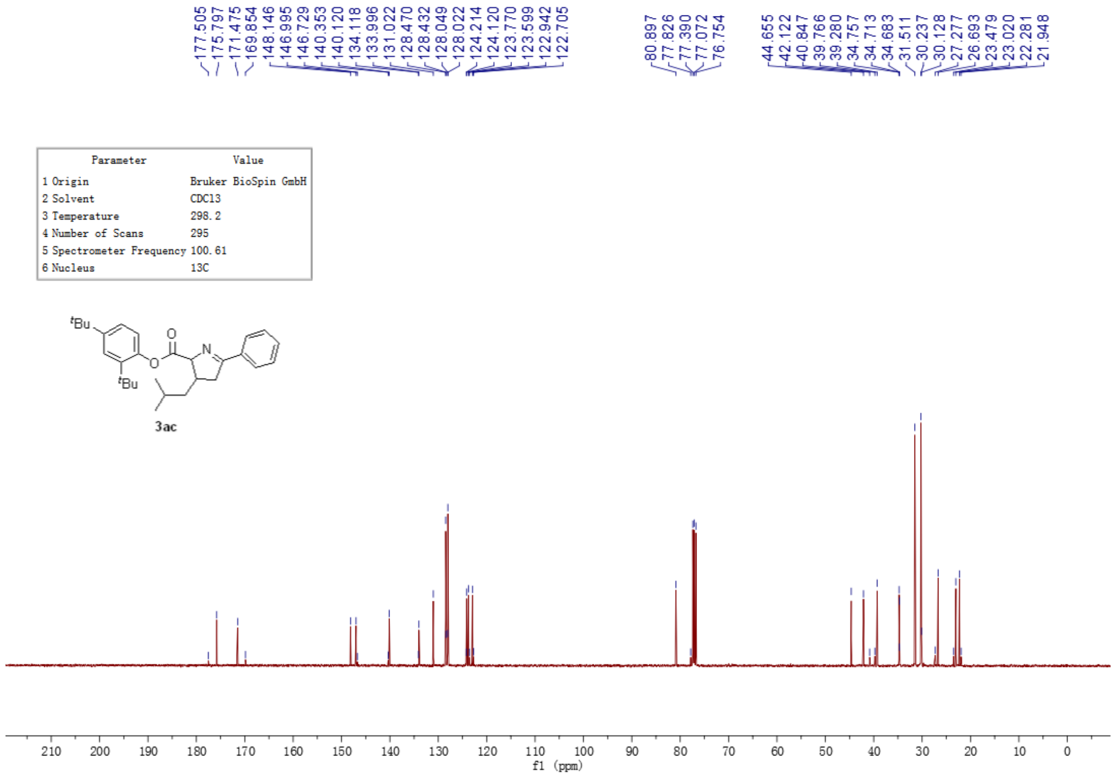
Parameter Value
1 Origin Bruker BioSpin GmbH
2 Solvent CDCl3
3 Temperature 298.2
4 Number of Scans 295
5 Spectrometer Frequency 100.61
6 Nucleus 13C
3ac
f1 (ppm)

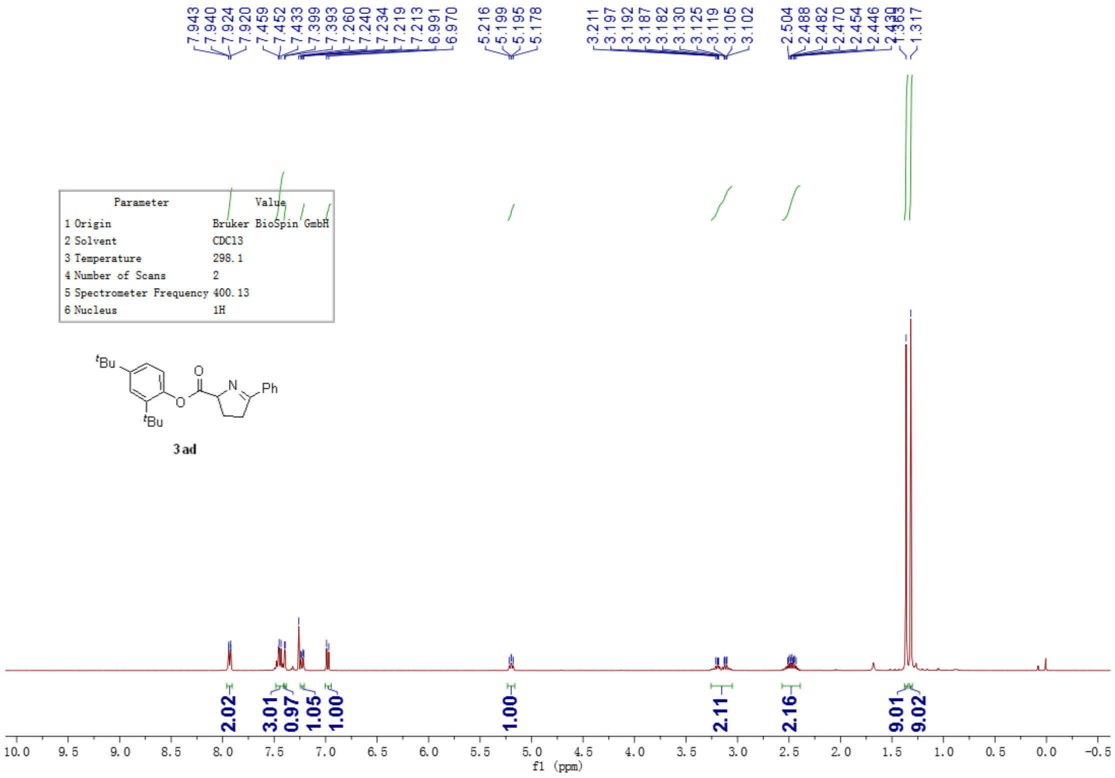
Parameter Value
1 Origin Bruker BioSpin GmbH
2 Solvent CDCl3
3 Temperature 298.1
4 Number of Scans 2
5 Spectrometer Frequency 400.13
6 Nucleus 1H
3ad
f1 (ppm)

Parameter	Value
1 Origin	Bruker BioSpin GmbH
2 Solvent	CDCl3
3 Temperature	296.5
4 Number of Scans	80
5 Spectrometer Frequency	100.62
6 Nucleus	13C

3ad

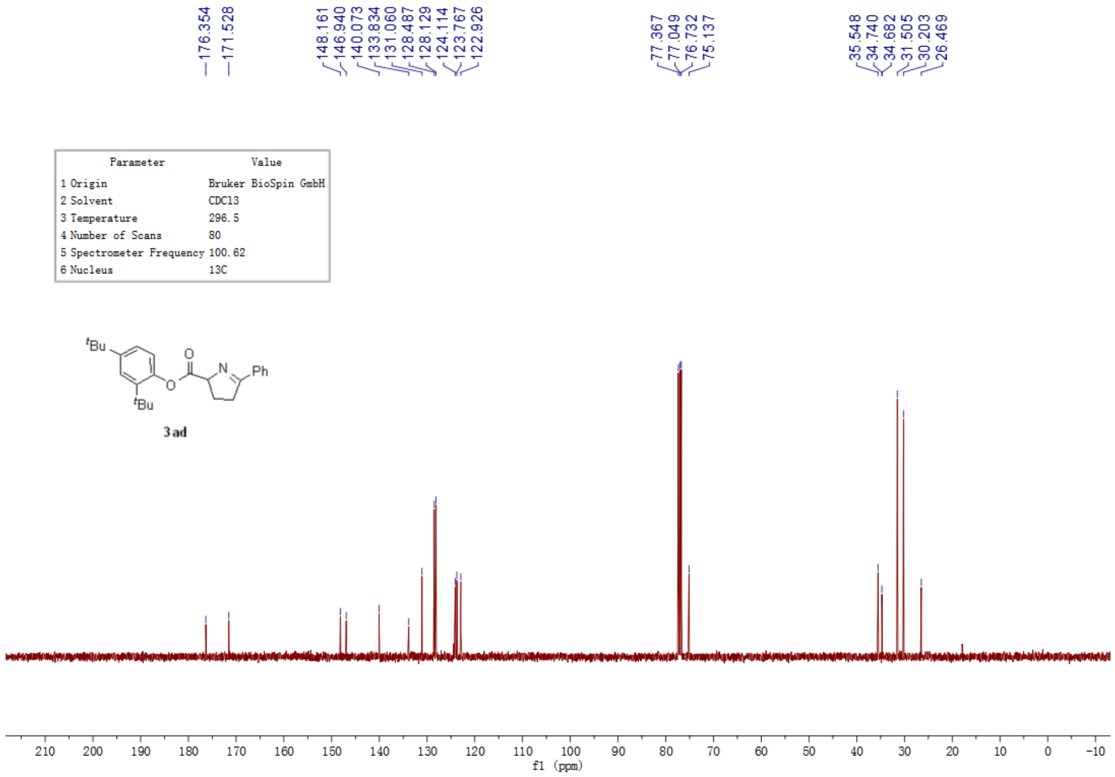

Parameter	Value
1 Origin	Bruker BioSpin GmbH
2 Solvent	CDCl3
3 Temperature	298.2
4 Number of Scans	2
5 Spectrometer Frequency	400.13
6 Nucleus	1H

3ae

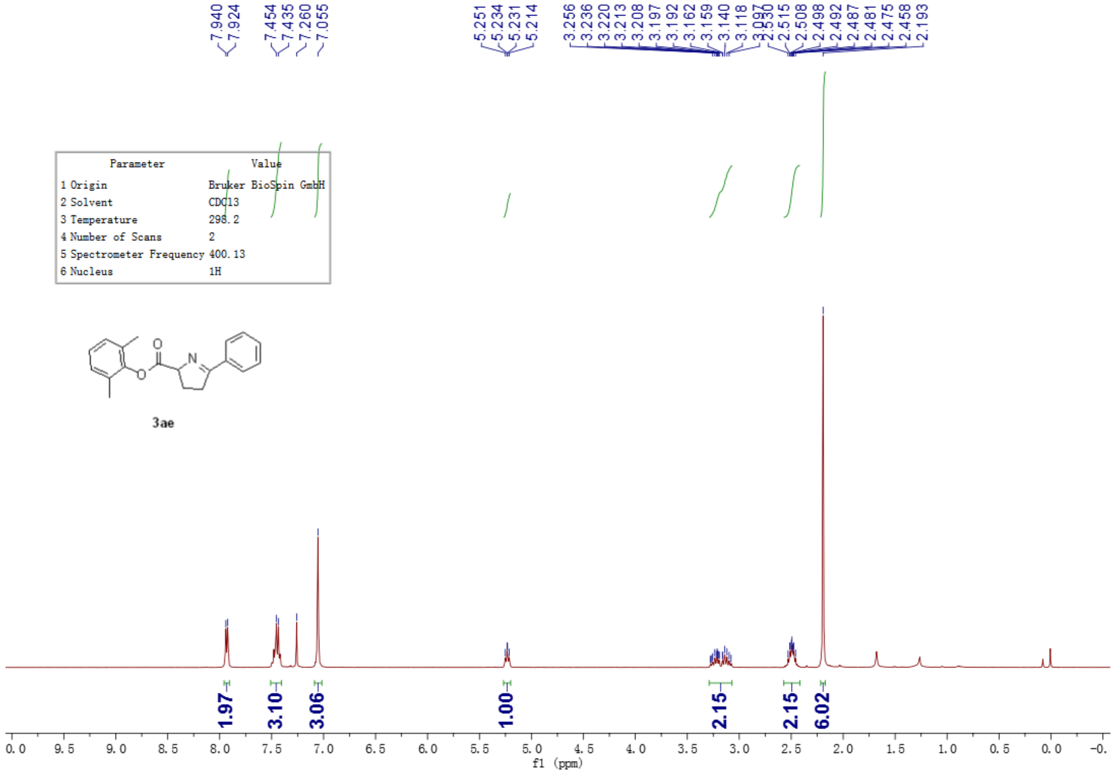

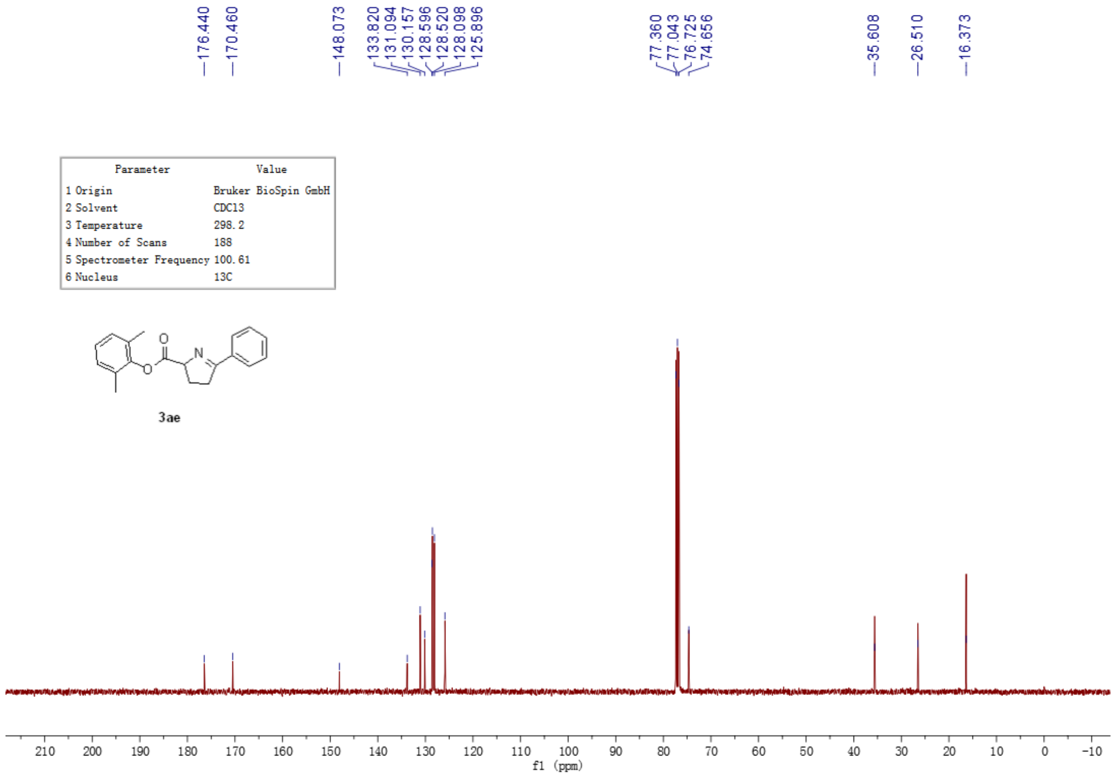
176.440
170.460
148.073
133.820
131.094
130.157
128.596
128.520
128.098
125.896
77.360
77.043
76.725
74.656
35.608
26.510
16.373
Parameter Value
1 Origin Bruker BioSpin GmbH
2 Solvent CDCl3
3 Temperature 298.2
4 Number of Scans 188
5 Spectrometer Frequency 100.61
6 Nucleus 13C
O
N
O
3ae
f1 (ppm)

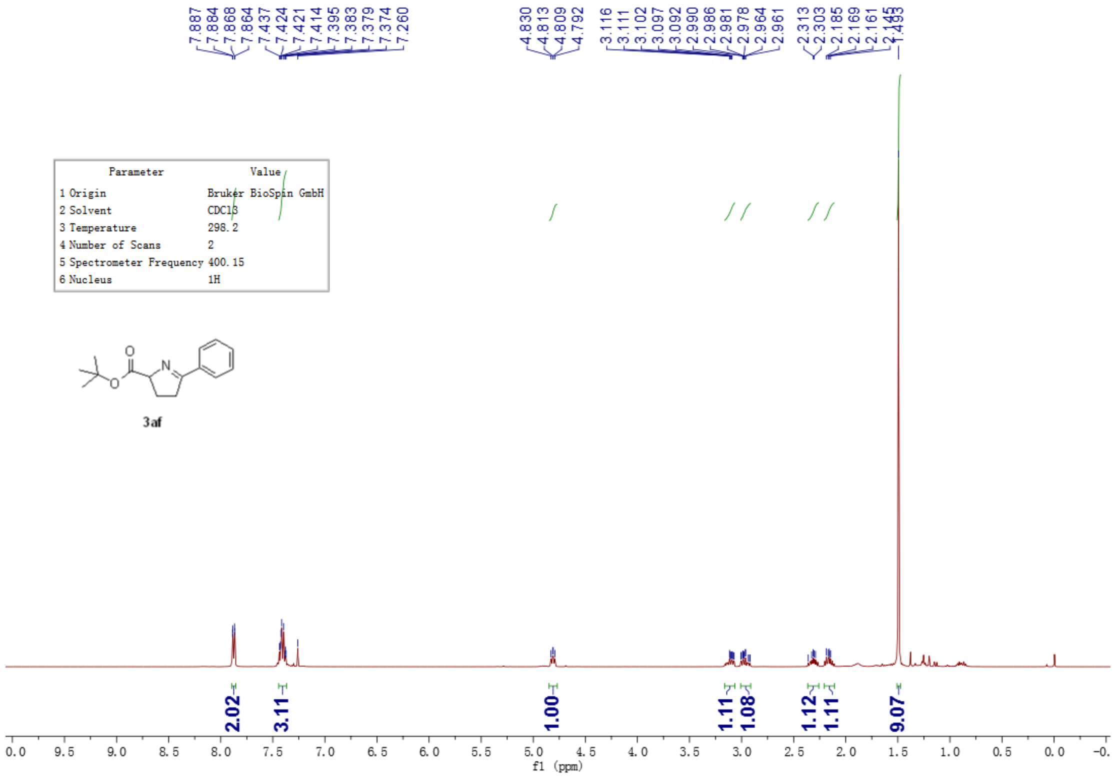
7.887
7.884
7.868
7.864
7.437
7.424
7.421
7.414
7.395
7.383
7.379
7.374
7.260
4.830
4.813
4.809
4.792
3.116
3.111
3.102
3.097
3.092
2.990
2.986
2.981
2.978
2.964
2.961
2.313
2.303
2.185
2.169
2.161
2.145
1.493
Parameter Value
1 Origin Bruker BioSpin GmbH
2 Solvent CDCl3
3 Temperature 298.2
4 Number of Scans 2
5 Spectrometer Frequency 400.15
6 Nucleus 1H
O
N
O
3af
2.02
3.11
1.00
1.11
1.08
1.12
1.11
9.07
f1 (ppm)

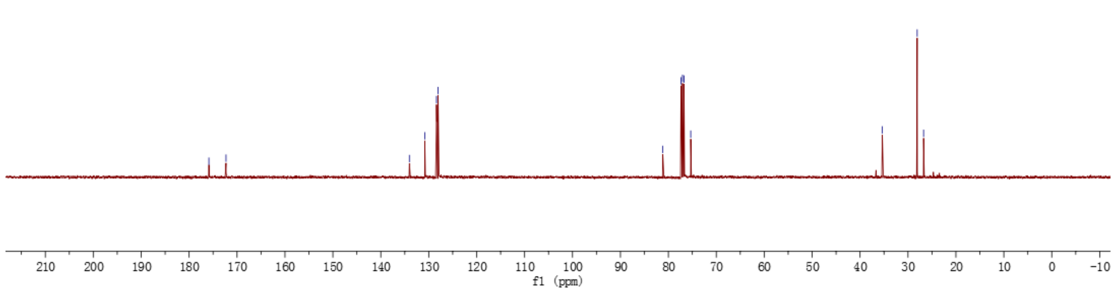

	Parameter	Value
1	Origin	Bruker BioSpin GmbH
2	Solvent	CDCl3
3	Temperature	298.1
4	Number of Scans	80
5	Spectrometer Frequency	100.62
6	Nucleus	13C

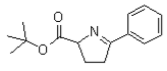

3af

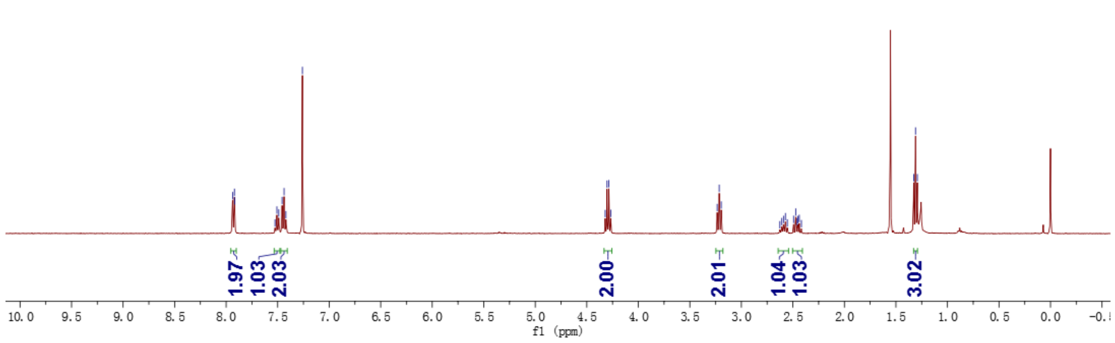

	Parameter	Value
1	Origin	Bruker BioSpin GmbH
2	Solvent	CDCl3
3	Temperature	298.2
4	Number of Scans	2
5	Spectrometer Frequency	400.15
6	Nucleus	1H

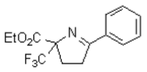

3ag

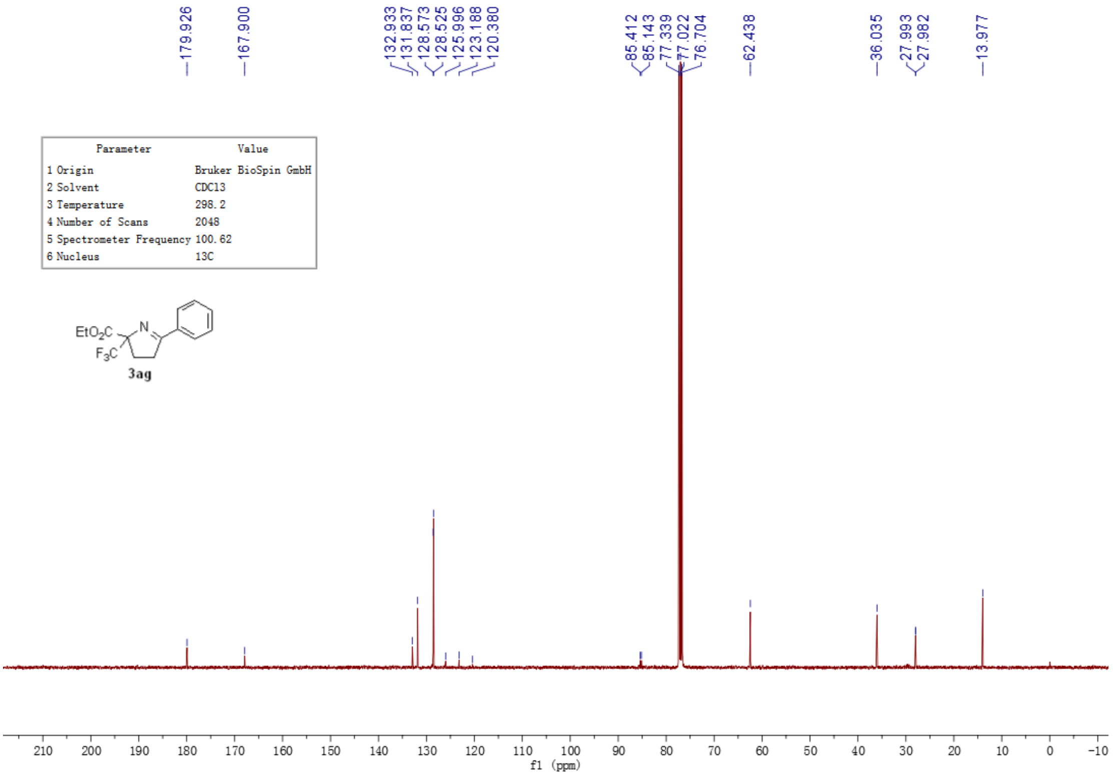

Parameter	Value
1 Origin	Bruker BioSpin GmbH
2 Solvent	CDCl3
3 Temperature	298.2
4 Number of Scans	2048
5 Spectrometer Frequency	100.62
6 Nucleus	13C

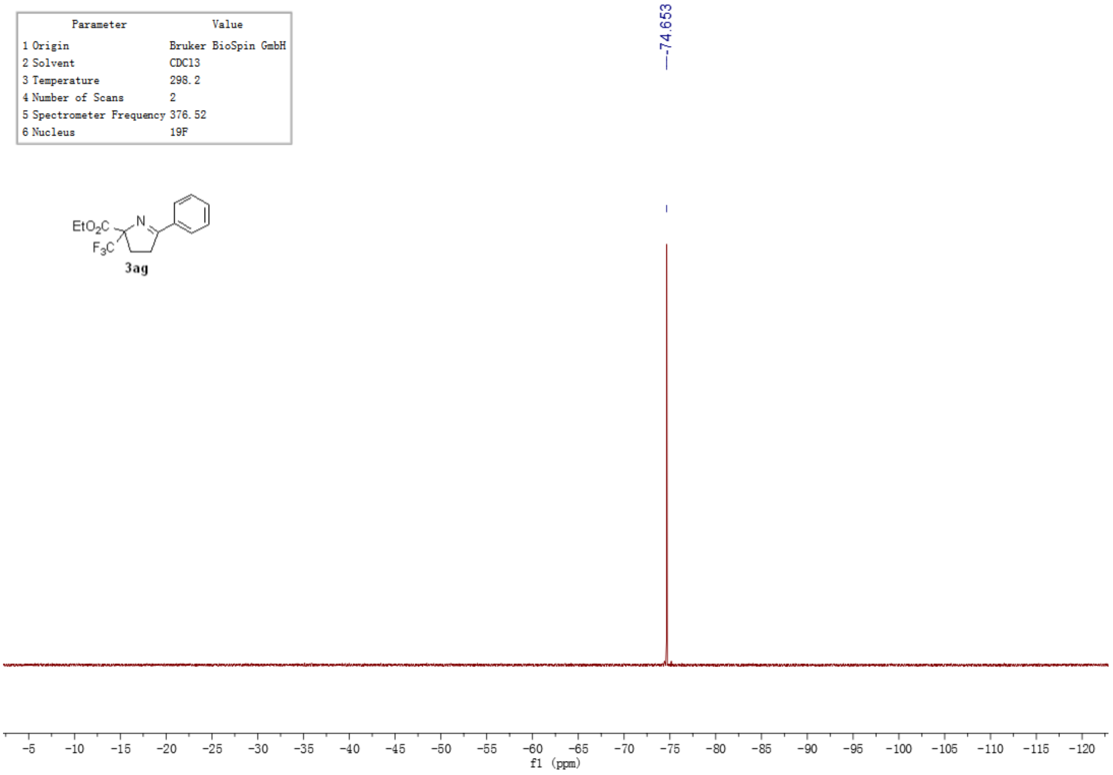

Parameter	Value
1 Origin	Bruker BioSpin GmbH
2 Solvent	CDCl3
3 Temperature	298.2
4 Number of Scans	2
5 Spectrometer Frequency	376.52
6 Nucleus	19F

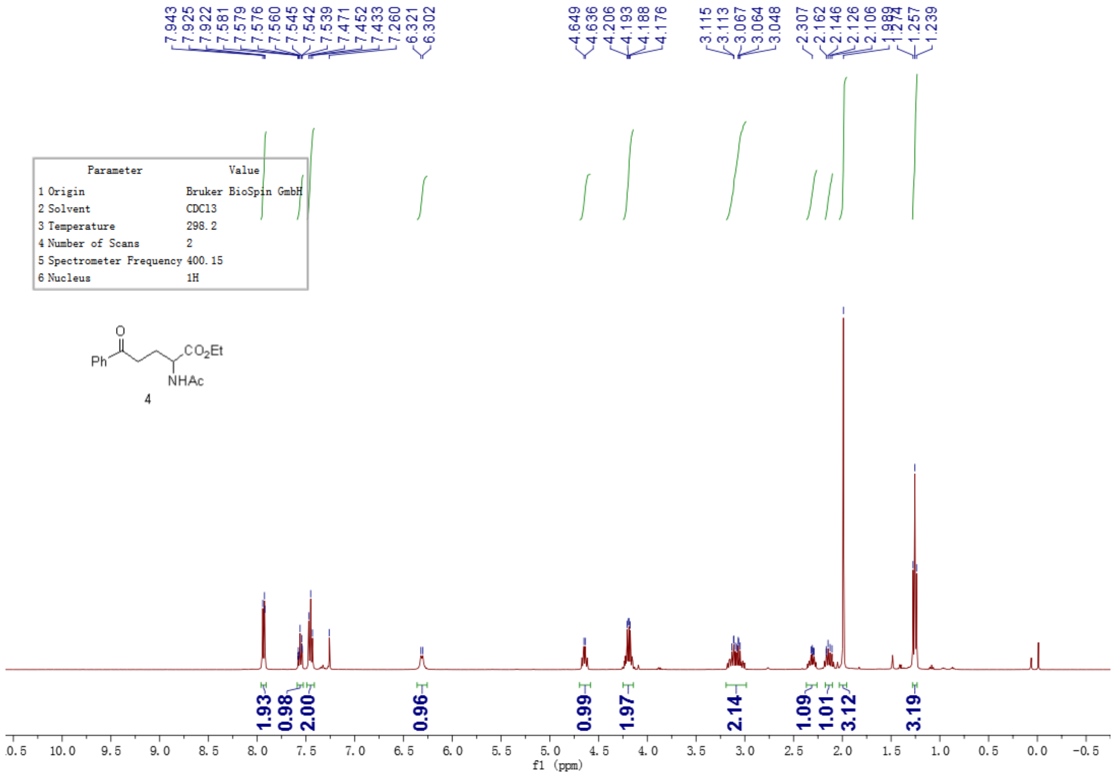
7.943
7.925
7.922
7.581
7.579
7.576
7.560
7.545
7.542
7.539
7.471
7.452
7.433
7.260
6.321
6.302
4.649
4.636
4.206
4.193
4.188
4.176
3.115
3.113
3.067
3.064
3.048
2.307
2.162
2.146
2.126
2.106
1.989
1.274
1.257
1.239
Parameter
Value
1 Origin
Bruker BioSpin GmbH
2 Solvent
CDC13
3 Temperature
298.2
4 Number of Scans
2
5 Spectrometer Frequency
400.15
6 Nucleus
1H
Ph
O
CO_2Et
NHAc
4
1.93
0.98
2.00
0.96
0.99
1.97
2.14
1.09
1.01
3.12
3.19
f1 (ppm)

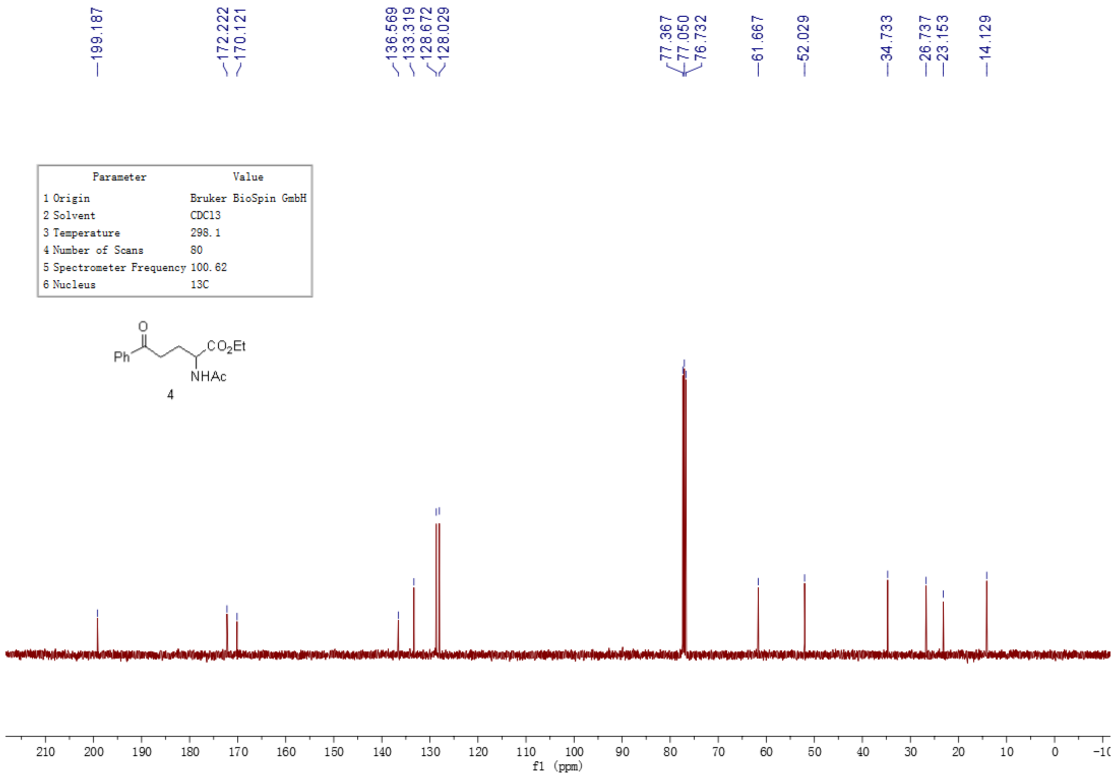
199.187
172.222
170.121
136.569
133.319
128.672
128.029
77.367
77.050
76.732
61.667
52.029
34.733
26.737
23.153
14.129
Parameter
Value
1 Origin
Bruker BioSpin GmbH
2 Solvent
CDC13
3 Temperature
298.1
4 Number of Scans
80
5 Spectrometer Frequency
100.62
6 Nucleus
13C
Ph
O
CO_2Et
NHAc
4
f1 (ppm)

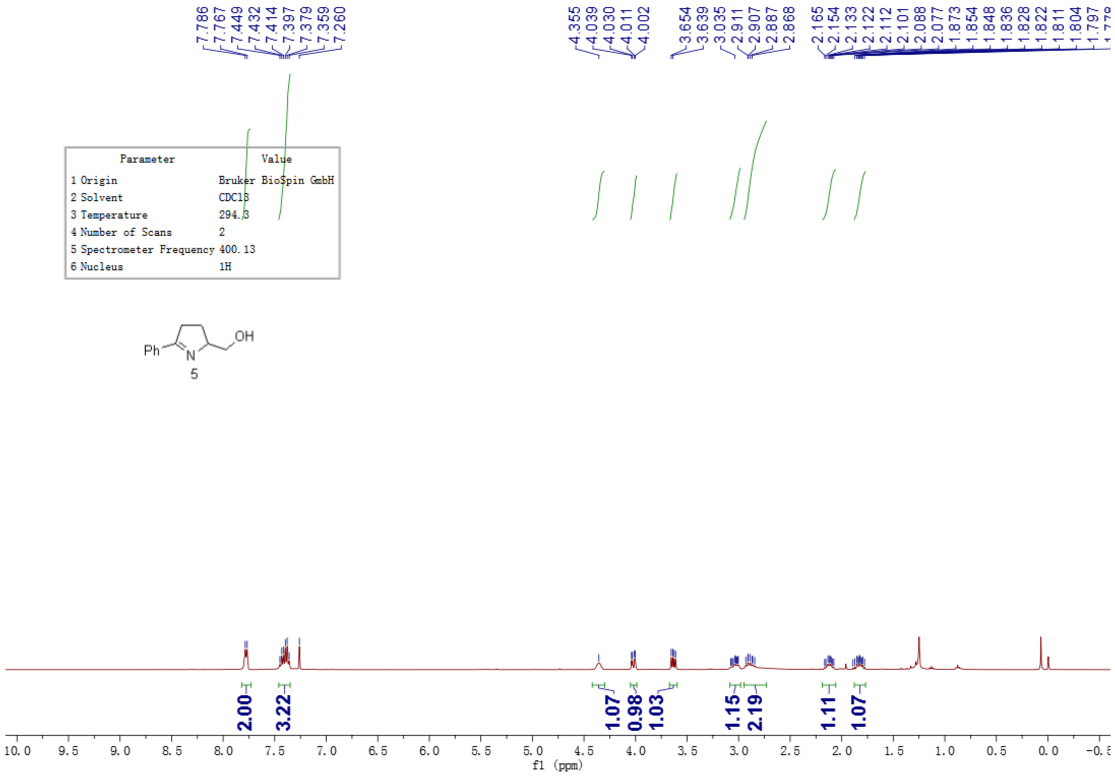
7.786
7.767
7.449
7.432
7.414
7.397
7.379
7.359
7.260
4.355
4.039
4.030
4.011
4.002
3.654
3.639
3.035
2.911
2.907
2.887
2.868
2.165
2.154
2.133
2.122
2.112
2.101
2.088
2.077
1.873
1.854
1.848
1.836
1.828
1.822
1.811
1.804
1.797
Parameter Value
1 Origin Bruker BioSpin GmbH
2 Solvent CDCl3
3 Temperature 294.3
4 Number of Scans 2
5 Spectrometer Frequency 400.13
6 Nucleus 1H
Ph
N
OH
5
2.00
3.22
1.07
0.98
1.03
1.15
2.19
1.11
1.07
f1 (ppm)

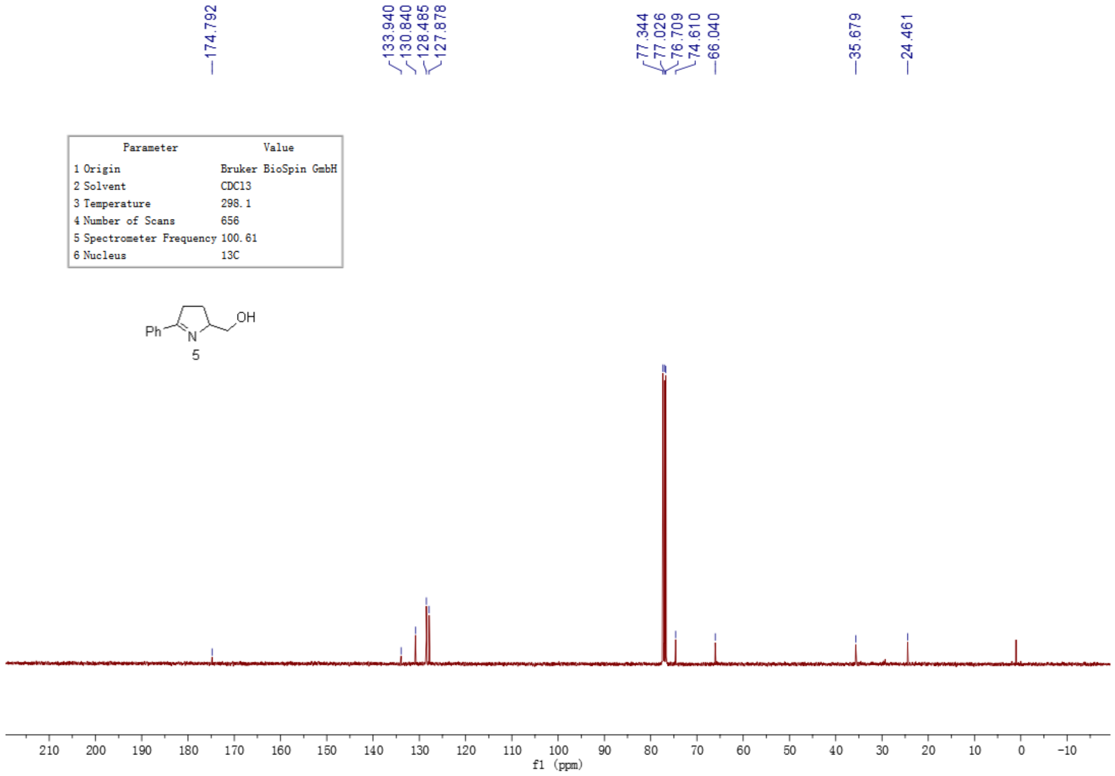
174.792
133.940
130.840
128.485
127.878
77.344
77.026
76.709
74.610
66.040
35.679
24.461
Parameter Value
1 Origin Bruker BioSpin GmbH
2 Solvent CDCl3
3 Temperature 298.1
4 Number of Scans 656
5 Spectrometer Frequency 100.61
6 Nucleus 13C
Ph
N
OH
5
f1 (ppm)

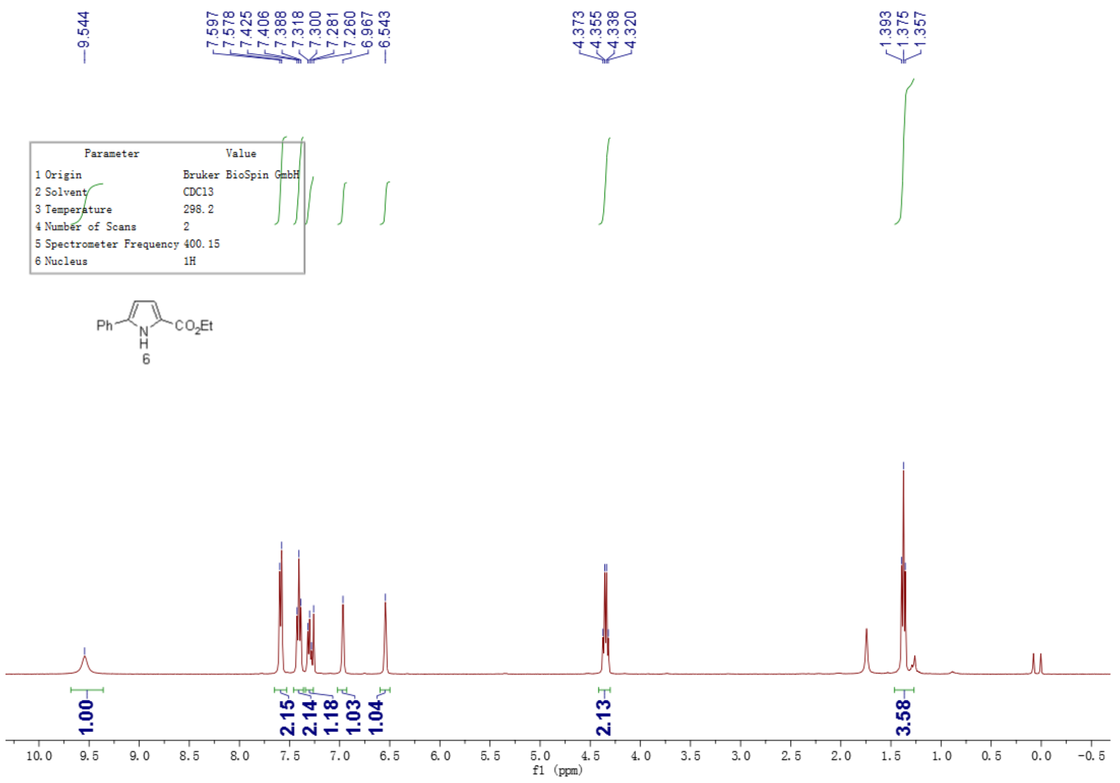

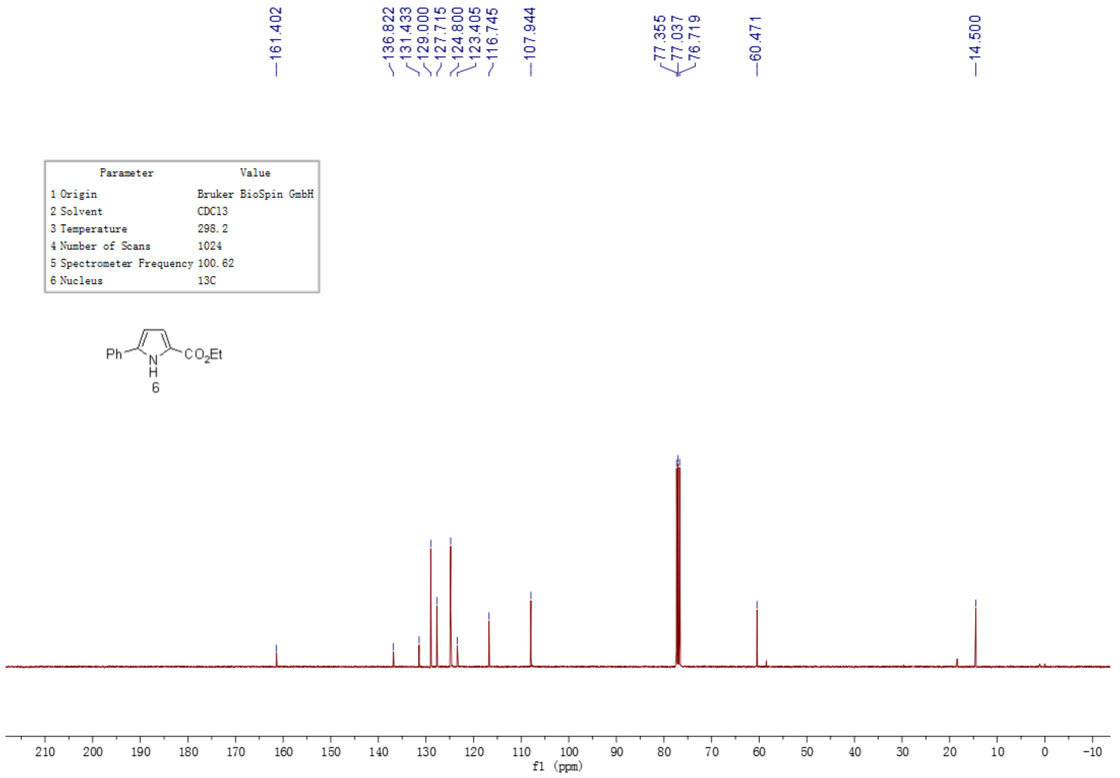

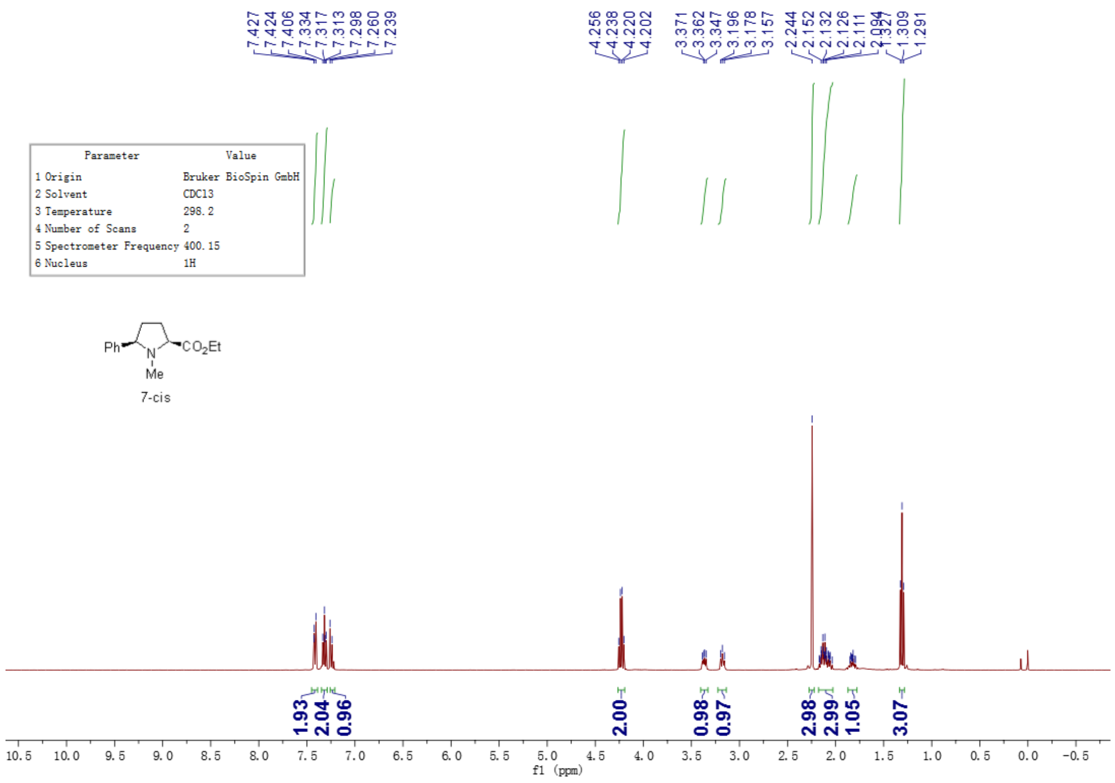

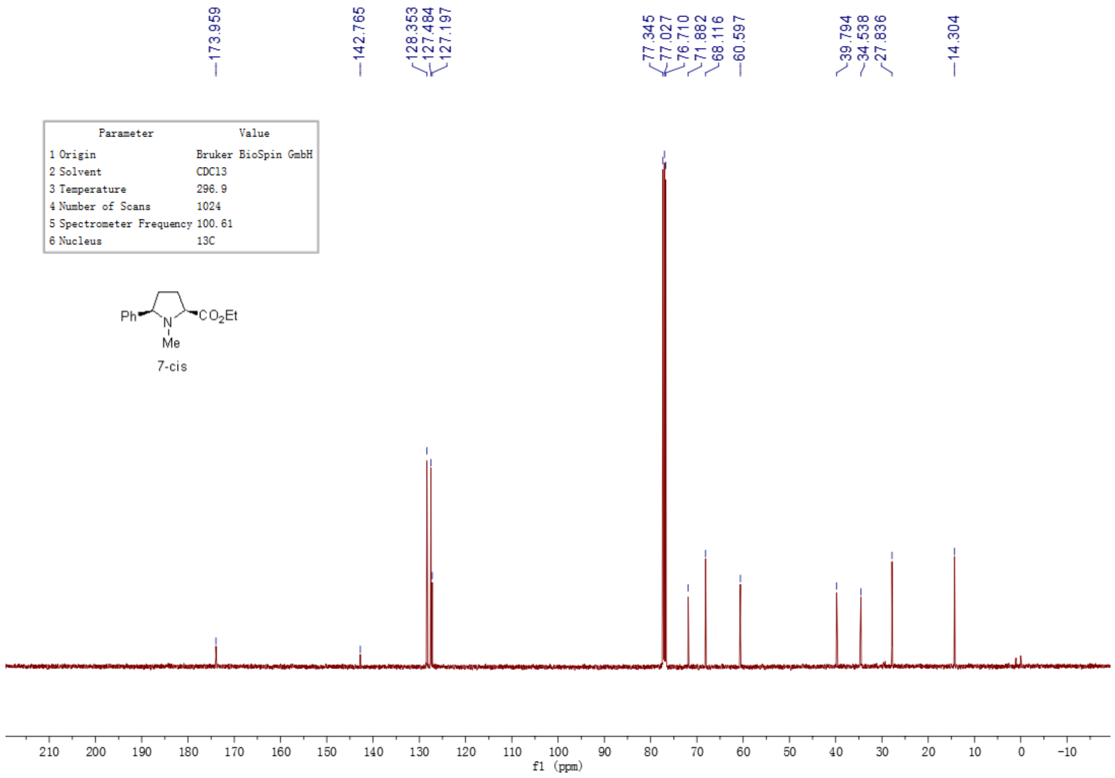

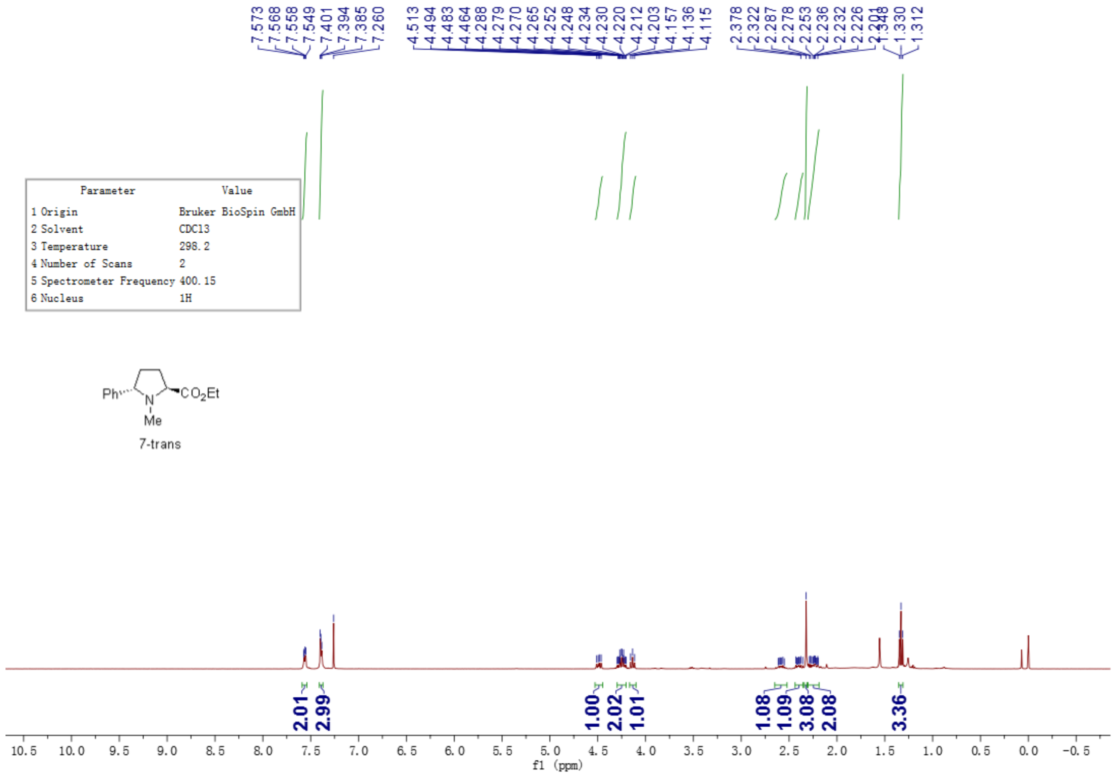

7.573
7.568
7.558
7.549
7.401
7.394
7.385
7.260
4.513
4.494
4.483
4.464
4.288
4.279
4.270
4.265
4.252
4.248
4.234
4.230
4.220
4.212
4.203
4.157
4.136
4.115
2.378
2.322
2.287
2.278
2.253
2.236
2.232
2.226
1.348
1.330
1.312
Parameter Value
1 Origin Bruker BioSpin GmbH
2 Solvent CDCl3
3 Temperature 298.2
4 Number of Scans 2
5 Spectrometer Frequency 400.15
6 Nucleus 1H
Ph
N
CO2Et
Me
7-trans
2.01
2.99
1.00
2.02
1.01
1.08
1.09
3.08
2.08
3.36
f1 (ppm)

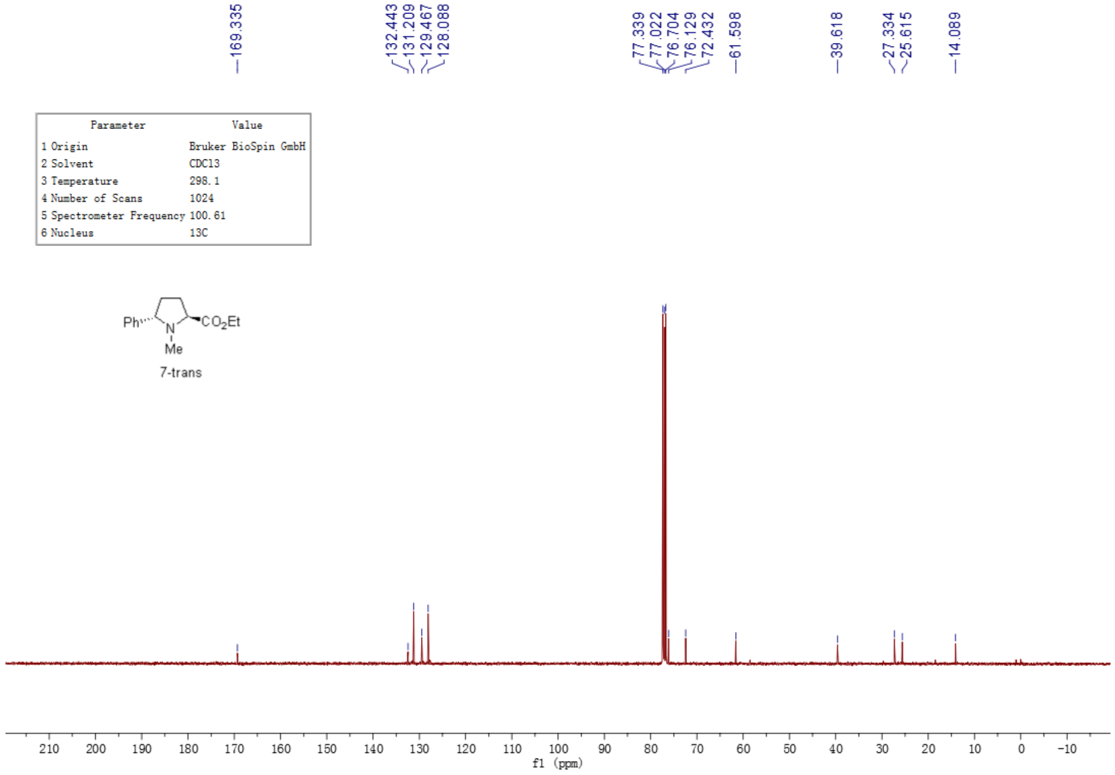

169.335
132.443
131.209
129.467
128.088
77.339
77.022
76.704
76.129
72.432
61.598
39.618
27.334
25.615
14.089
Parameter Value
1 Origin Bruker BioSpin GmbH
2 Solvent CDCl3
3 Temperature 298.1
4 Number of Scans 1024
5 Spectrometer Frequency 100.61
6 Nucleus 13C
Ph
N
CO2Et
Me
7-trans
f1 (ppm)
